# Development of treatment-decision algorithms for children evaluated for pulmonary tuberculosis: an individual participant data meta-analysis

**DOI:** 10.1016/S2352-4642(23)00004-4

**Published:** 2023-05

**Authors:** Kenneth S Gunasekera, Olivier Marcy, Johanna Muñoz, Elisa Lopez-Varela, Moorine P Sekadde, Molly F Franke, Maryline Bonnet, Shakil Ahmed, Farhana Amanullah, Aliya Anwar, Orvalho Augusto, Rafaela Baroni Aurilio, Sayera Banu, Iraj Batool, Annemieke Brands, Kevin P Cain, Lucía Carratalá-Castro, Maxine Caws, Eleanor S Click, Lisa M Cranmer, Alberto L García-Basteiro, Anneke C Hesseling, Julie Huynh, Senjuti Kabir, Leonid Lecca, Anna Mandalakas, Farai Mavhunga, Aye Aye Myint, Kyaw Myo, Dorah Nampijja, Mark P Nicol, Patrick Orikiriza, Megan Palmer, Clemax Couto Sant'Anna, Sara Ahmed Siddiqui, Jonathan P Smith, Rinn Song, Nguyen Thuy Thuong Thuong, Vibol Ung, Marieke M van der Zalm, Sabine Verkuijl, Kerri Viney, Elisabetta G Walters, Joshua L Warren, Heather J Zar, Ben J Marais, Stephen M Graham, Thomas P A Debray, Ted Cohen, James A Seddon

**Affiliations:** a Department of Epidemiology of Microbial Diseases, Yale School of Public Health, New Haven, CT, USA; b Department of Health Policy and Management, Yale School of Public Health, New Haven, CT, USA; c Department of Biostatistics, Yale School of Public Health, New Haven, CT, USA; d Inserm UMR1219, Institut de Recherche pour le Développement EMR 271, GHiGS, University of Bordeaux, Bordeaux, France; e Julius Center for Health Sciences and Primary Care, University Medical Center Utrecht, Utrecht University, Utrecht, Netherlands; f ISGlobal, Hospital Clínic - Universitat de Barcelona, Barcelona, Spain; g Centro de Investigação em Saúde de Manhiça, Maputo, Mozambique; h National Tuberculosis and Leprosy Program, Kampala, Uganda; i Department of Global Health and Social Medicine, Harvard Medical School, Boston, MA, USA; j Department of Pediatrics, Harvard Medical School, Boston, MA, USA; k University of Montpellier, TransVIHMI, Institut de Recherche pour le Développement, Inserm, Montpellier, France; l Epicentre, Mbarara, Uganda; m Department of Paediatrics, Dhaka Medical College Hospital, Dhaka, Bangladesh; n Indus Hospital & Health Network, Karachi, Pakistan; o The Aga Khan University Hospital, Karachi, Pakistan; p Instituto de Puericultura e Pediatria Martagao Gesteira, Universidade Federal do Rio de Janeiro, Rio de Janeiro, Brazil; q Faculty of Medicine, Universidade Federal do Rio de Janeiro, Rio de Janeiro, Brazil; r Programme on Emerging Infections, Infectious Disease Division, icddr,b, Dhaka, Bangladesh; s Global Tuberculosis Programme, WHO, Geneva, Switzerland; t US Centers for Disease Control and Prevention, Atlanta, GA, USA; u Department of Clinical Sciences, Liverpool School of Tropical Medicine, Liverpool, UK; v Birat Nepal Medical Trust, Lazmipat, Kathmandu, Nepal; w Department of Pediatrics, Emory School of Medicine, Atlanta, GA, USA; x Department of Epidemiology, Emory Rollins School of Public Health, Atlanta, GA, USA; y Children's Healthcare of Atlanta, Atlanta, GA, USA; z Centro de Investigación Biomédica en Red de Enfermedades Infecciosas, Barcelona, Spain; aa Desmond Tutu TB Centre, Department of Paediatrics and Child Health, Faculty of Medicine and Health Sciences, Stellenbosch University, Tygerberg, South Africa; ab Oxford University Clinical Research Unit, Centre for Tropical Diseases, Ho Chi Minh City, Viet Nam; ac Nuffield Department of Medicine, Centre for Tropical Medicine and Global Health, University of Oxford, Oxford, UK; ad Oxford Vaccine Group, Department of Paediatrics, University of Oxford, Oxford, UK; ae Socios En Salud Surcursal Perú, Lima, Perú; af Global TB Program, Baylor College of Medicine and Texas Children's Hospital, Houston, TX, USA; ag Clinical Infectious Disease Group, German Center for Infectious Research, Clinical TB Unit, Research Center Borstel, Borstel, Germany; ah Department of Paediatrics, University of Medicine, Mandalay, Myanmar; ai Department of Paediatrics, University of Medicine, Magway, Myanmar; aj Department of Paediatrics, Mbarara University of Science and Technology, Mbarara, Uganda; ak Division of Infection and Immunity, Department of Biomedical Sciences, University of Western Australia, Perth, WA, Australia; al Department of Microbiology, Division of Basic Medical Sciences, School of Medicine, University of Global Health Equity, Kigali, Rwanda; am University of Health Sciences, Phnom Penh, Cambodia; an National Pediatric Hospital, Phnom Penh, Cambodia; ao School of Public Health, University of Sydney, Sydney, NSW, Australia; ap The Children's Hospital at Westmead Clinical School, Faculty of Medicine and Health, University of Sydney, Sydney, NSW, Australia; aq Directorate of Integrated Laboratory Medicine, Institute of Human Genetics, Newcastle upon Tyne NHS Foundation Trust, Newcastle upon Tyne, UK; ar Department of Paediatrics and Child Health, Red Cross Children's Hospital, and SA-MRC Unit on Child and Adolescent Health, University of Cape Town, Cape Town, South Africa; as Department of Paediatrics and Murdoch Children's Research Institute, Royal Children's Hospital, University of Melbourne, Melbourne, VIC, Australia; at Burnet Institute, Melbourne, VIC, Australia; au Department of Infectious Diseases, Imperial College London, London, UK

## Abstract

**Background:**

Many children with pulmonary tuberculosis remain undiagnosed and untreated with related high morbidity and mortality. Recent advances in childhood tuberculosis algorithm development have incorporated prediction modelling, but studies so far have been small and localised, with limited generalisability. We aimed to evaluate the performance of currently used diagnostic algorithms and to use prediction modelling to develop evidence-based algorithms to assist in tuberculosis treatment decision making for children presenting to primary health-care centres.

**Methods:**

For this meta-analysis, we identified individual participant data from a WHO public call for data on the management of tuberculosis in children and adolescents and referral from childhood tuberculosis experts. We included studies that prospectively recruited consecutive participants younger than 10 years attending health-care centres in countries with a high tuberculosis incidence for clinical evaluation of pulmonary tuberculosis. We collated individual participant data including clinical, bacteriological, and radiological information and a standardised reference classification of pulmonary tuberculosis. Using this dataset, we first retrospectively evaluated the performance of several existing treatment-decision algorithms. We then used the data to develop two multivariable prediction models that included features used in clinical evaluation of pulmonary tuberculosis—one with chest x-ray features and one without—and we investigated each model's generalisability using internal–external cross-validation. The parameter coefficient estimates of the two models were scaled into two scoring systems to classify tuberculosis with a prespecified sensitivity target. The two scoring systems were used to develop two pragmatic, treatment-decision algorithms for use in primary health-care settings.

**Findings:**

Of 4718 children from 13 studies from 12 countries, 1811 (38·4%) were classified as having pulmonary tuberculosis: 541 (29·9%) bacteriologically confirmed and 1270 (70·1%) unconfirmed. Existing treatment-decision algorithms had highly variable diagnostic performance. The scoring system derived from the prediction model that included clinical features and features from chest x-ray had a combined sensitivity of 0·86 [95% CI 0·68–0·94] and specificity of 0·37 [0·15–0·66] against a composite reference standard. The scoring system derived from the model that included only clinical features had a combined sensitivity of 0·84 [95% CI 0·66–0·93] and specificity of 0·30 [0·13-0·56] against a composite reference standard. The scoring system from each model was placed after triage steps, including assessment of illness acuity and risk of poor tuberculosis-related outcomes, to develop treatment-decision algorithms.

**Interpretation:**

We adopted an evidence-based approach to develop pragmatic algorithms to guide tuberculosis treatment decisions in children, irrespective of the resources locally available. This approach will empower health workers in primary health-care settings with high tuberculosis incidence and limited resources to initiate tuberculosis treatment in children to improve access to care and reduce tuberculosis-related mortality. These algorithms have been included in the operational handbook accompanying the latest WHO guidelines on the management of tuberculosis in children and adolescents. Future prospective evaluation of algorithms, including those developed in this work, is necessary to investigate clinical performance.

**Funding:**

WHO, US National Institutes of Health.

## Introduction

Tuberculosis is a leading cause of mortality among children worldwide,^1^ accounting for about 2·5% of the 6 million deaths in children younger than 5 years each year.^2^ Modelling suggests that more than 96% of tuberculosis deaths in children younger than 15 years occurred in those not receiving tuberculosis treatment.^3^ WHO estimates that fewer than 50% of the 1·1 million children younger than 15 years who develop tuberculosis are diagnosed; the proportion is even lower, about 30%, among children younger than 5 years.^1^ Thus, efforts to improve diagnosis, and thereby improve access to tuberculosis treatment, are important to reduce tuberculosis morbidity and deaths in children.

Confirmation of pulmonary tuberculosis in children is challenging because respiratory specimens tend to be paucibacillary.^4^ Furthermore, collecting respiratory specimens from young children is invasive and requires resources that are generally concentrated in higher-level health-care centres. Thus, careful symptom review, clinical examination, chest x-ray, and history of *Mycobacterium tuberculosis* exposure can inform treatment decisions in clinical care. However, paediatric clinical expertise to make a diagnosis is often insufficient at primary health-care centres. This limits treatment access and leads to either delays in treatment initiation or no treatment initiation, both of which are associated with poor outcomes, including mortality.^5^, ^6^ Facilitation of appropriate diagnostic assessment with rapid treatment initiation at primary health-care settings where children initially present could contribute to reductions in tuberculosis-related morbidity and mortality.

**Research in context**

**Evidence before the study**

Treatment-decision algorithms relate information gained in the evaluation of children into an assessment of tuberculosis disease risk and empower health-care workers to make appropriate treatment decisions. Studies in primary health-care centres have shown that use of treatment-decision algorithms can improve childhood pulmonary tuberculosis case detection and treatment initiation in settings with a high incidence of tuberculosis. We searched PubMed using the terms (“child*” OR “paediatr*” OR “pediatr*”) AND (“tuberculosis” OR “TB”) AND (“treatment-decision” OR “algorithm” OR “diagnos*”) to identify primary research studies on childhood pulmonary tuberculosis treatment-decision algorithm performance evaluation or development published in any language before June 29, 2022. We additionally consulted several experts in childhood pulmonary tuberculosis diagnosis and management, and we referred to existing, published reviews of treatment-decision algorithms. With respect to performance, several studies have retrospectively estimated the performance of treatment-decision algorithms in a single geographical setting; a subset of these studies have also compared the performance of multiple algorithms using data from a single geographical setting. With respect to development, many existing algorithms have been developed without explicit analysis of data from children with presumptive pulmonary tuberculosis, often developed from expert consensus. Gunasekera and colleagues used model-based approaches to analyse diagnostic evaluations data (eg, clinical history, physical examination, chest radiograph, and results from rapid molecular and culture testing for *Mycobacterium tuberculosis*) collected from children with presumptive pulmonary tuberculosis in a single geographical setting to inform the development of a diagnostic algorithm, whereas Marcy and colleagues and Fourie and colleagues analysed data from multiple geographical settings. However, these studies were relatively small with limited assessment of generalisability.

**Added value of this study**

Following a WHO call for data, we identified and collated individual participant data from 13 prospective diagnostic studies from 12 countries including 4718 children with presumptive pulmonary tuberculosis from geographically diverse settings with a high incidence of tuberculosis. We evaluated the performance of existing treatment-decision algorithms and developed multivariable logistic regression models to quantify the contribution of individual features to discriminate tuberculosis from non-tuberculosis. A panel of child tuberculosis experts provided input into performance targets and advised on how to incorporate scores derived from these models into pragmatic treatment-decision algorithms to assist in the evaluation of children presenting with presumptive pulmonary tuberculosis in primary health-care centres.

**Implications of all the available evidence**

Our findings suggest that evidence-based, pragmatic treatment-decision algorithms can be developed to make sensitive and clinically appropriate decisions to treat a child with pulmonary tuberculosis. Although the specificity does not reach optimal targets for childhood tuberculosis diagnosis, pragmatic treatment-decision algorithms provide clinically relevant guidance that can empower health workers to start children on tuberculosis treatment in the primary health-care setting and could contribute to reducing the case-detection gap in childhood tuberculosis. External, prospective evaluation of these novel algorithms in diverse settings is required, including assessment of their accuracy, feasibility, acceptability, impact, and cost-effectiveness. This work led to a new interim WHO recommendation to support the use of treatment-decision algorithms in the evaluation of children with presumptive tuberculosis in the 2022 updated consolidated guidelines on the management of tuberculosis in children. Two algorithms developed from this work have been included in the WHO operational handbook accompanying these guidelines.

Treatment-decision algorithms aim to standardise clinical assessment and decision making. Algorithms relate information gained in the evaluation of children into an assessment of tuberculosis disease risk and empower health-care workers to make appropriate treatment decisions. Adoption of an algorithmic approach to treatment decision making has been shown to improve childhood tuberculosis case detection and treatment access at primary health-care settings.^7^, ^8^ However, these algorithms were developed using consensus expert opinion rather than analysis of data.

Recent approaches for algorithm generation have used data from cross-sectional childhood tuberculosis diagnostic studies to quantify the contribution of clinical characteristics to the risk of tuberculosis disease.^9^, ^10^, ^11^ Evidence-based approaches are objective and offer the potential for validation; however, existing studies have been small and not generalisable. We assembled individual participant data from children investigated for presumptive pulmonary tuberculosis. We then aimed to evaluate the performance of currently used diagnostic algorithms and to develop evidence-based algorithms to assist in tuberculosis treatment decision making for children younger than 10 years presenting to primary health-care settings. This work was conducted to inform the 2022 WHO guidelines for the management of tuberculosis in children and adolescents and the accompanying WHO operational handbook.^12^, ^13^

## Methods

### Establishment of individual participant data

We identified potential sources of individual participant data through responses to a WHO public call for data on the management of tuberculosis in children and adolescents in July, 2020,^14^ and through referral from childhood tuberculosis experts. Studies were eligible for inclusion if they prospectively recruited consecutive participants younger than 10 years attending health-care centres in countries with a high tuberculosis incidence for clinical evaluation of pulmonary tuberculosis and if they provided standardised reference classifications of pulmonary tuberculosis. We used an age cutoff of younger than 10 years to be consistent with the WHO definition of a child and to acknowledge that tuberculosis in children aged 10 years or older presents more similarly to adult tuberculosis and that adult diagnostic approaches are commonly used in this age group. We preferred for studies to have used the US National Institutes of Health (NIH) clinical case definitions of intrathoracic tuberculosis in children for diagnostic studies,^15^ which classifies tuberculosis as follows: confirmed tuberculosis as culture-confirmed or Xpert MTB/RIF-confirmed *M tuberculosis* from respiratory specimens; unconfirmed tuberculosis as having symptoms, chest x-ray findings, immune tests of *M tuberculosis* sensitisation suggestive of tuberculosis, and follow-up to assess response to tuberculosis treatment (or without resolution of symptoms in the absence of tuberculosis treatment); and unlikely tuberculosis as not meeting criteria for either confirmed or unconfirmed tuberculosis. To ensure greater geographical representation, we also accepted data from high-quality studies that provided reference classifications using a previous NIH clinical case definition (in which the categories of probable and possible tuberculosis were combined into the unconfirmed tuberculosis category) and those that classified children using similar, prespecified definitions of confirmed, unconfirmed, and unlikely tuberculosis. Quality assessment was performed using a modified version of the Newcastle-Ottawa scale for cohort studies.^16^

After identification of eligible studies, we requested individual participant data including details from the clinical history, physical examination, chest x-ray, and results from rapid molecular and culture testing for *M tuberculosis* performed on respiratory specimens collected at study entry (appendix pp 3–6). All data assembly and analysis were carried out using R software (version 4.1.1). To account for the uncertainty associated with incomplete data, we used multilevel multiple imputation by chained equations (MICE) implemented in the mice package to generate 100 imputed datasets (appendix p 7).^17^ This study was approved by the Stellenbosch University (Cape Town, South Africa) Health Research Ethics Committee (reference number X21/02/003) and the Yale University (New Haven, CT, USA) Institutional Review Board (reference number 2000028046). All collaborating investigators confirmed institutional ethical approval for their original data collection.

### Evaluation of existing treatment-decision algorithms

We identified existing treatment-decision algorithms and scores (henceforth referred to as algorithms) to guide the evaluation of children with presumptive pulmonary tuberculosis through consultation with members of the WHO Guideline Development Group on the management of tuberculosis in children and adolescents. We defined a composite reference standard that includes confirmed and unconfirmed pulmonary tuberculosis to evaluate the performance of these algorithms. We carried out a sensitivity analysis of performance using a reference standard of confirmed pulmonary tuberculosis only (excluding children with unconfirmed tuberculosis). We used the reitsma function from the R package mada to pool study-level sensitivity and specificity estimates with 95% CIs using a bivariate random-effects meta-analysis (appendix p 8).^18^, ^19^

### Prediction model development and validation

We developed a multivariable logistic regression model to predict pulmonary tuberculosis using the composite reference standard in accordance with the Transparent Reporting of a Multivariable Prediction Model for Individual Prognosis or Diagnosis standards.^20^ We prespecified our model to include predictors from clinical and chest x-ray features commonly considered in the evaluation of presumptive childhood pulmonary tuberculosis in primary health-care settings with less than 50% missingness in our individual participant dataset. We also built a model without chest x-ray data to inform predictions in health-care centres without access to radiology services.

We adopted an internal–external cross-validation framework to estimate model parameters and assess generalisability.^21^ Briefly, this leave-one-study-out approach built the prediction model on *n* – 1 studies (*n* being the total number of studies included in the individual participant dataset) and validated using the remaining study, and is repeated for each hold-out study. Given that model performance was expected to vary across each of the hold-out studies, the regression coefficients of the *n* prediction models were subsequently meta-analysed to produce a single, summary prediction model. This approach was implemented in the metapred function of package metamisc, which accomplished meta-analysis via linear models.^21^, ^22^ To account for missing data, we generated a prediction model as described previously from each of the 100 imputed datasets and then used Rubin's rules to pool the regression coefficients and SEs to generate a final, single prediction model and compute odds ratios (ORs) with 95% CIs.^23^ Using the leave-one-study-out approach, we estimated the *c* statistic (also known as the area under the receiver operating characteristic curve) to assess the model's ability to distinguish between children with tuberculosis and unlikely tuberculosis, the calibration intercept, and the observed-to-expected (O:E) ratio, to assess whether there were studies in which the model over-predicted or under-predicted tuberculosis.

### Algorithm development

To generate clinically and programmatically implementable algorithms, we first converted the coefficient estimates of the parameters from each prediction model into a respective scoring system. We then placed the scoring system after several triage steps to guide health-care workers on its appropriate use, leading to two complete treatment-decision algorithms. We describe these steps in additional detail as follows.

We scaled the coefficient estimates for the parameters of the final prediction models (developed from all *n* studies) to estimate scores for each parameter such that a combined score of more than 10 corresponded to classification of tuberculosis at fixed sensitivities of 90%, 85%, 80%, 75%, and 70% (appendix p 9). To estimate the sensitivity and specificity of the scoring system in classifying tuberculosis using the composite reference standard, study-level sensitivities and specificities were pooled using the bivariate normal model of Reitsma and colleagues (implemented in the mada package) accounting for uncertainty introduced by imputation of missing data.^18^, ^19^ As a sensitivity analysis, we evaluated the performance of the score against a reference standard of confirmed pulmonary tuberculosis only.

We worked with staff from the WHO Global TB Programme to identify a group of experts in childhood tuberculosis (henceforth referred to as the expert group; appendix p 10) to advise on selection of a sensitivity performance target to develop the scoring system and development of triage steps before the scoring system to guide its appropriate use at primary health-care centres.

### Role of the funding source

The funders of the study had no role in study design, data collection, data analysis, data interpretation, writing of the report, or the decision to submit.

## Results

18 studies were identified as having potentially appropriate data, largely sourced from diagnostic evaluation studies (appendix p 11). The study investigators for two studies were unable to provide data in the necessary timeline, and an additional three studies did not meet the inclusion criteria. From the 13 included studies carried out in 12 countries, 4718 individual participant data records from children younger than 10 years with presumptive pulmonary tuberculosis were available (table 1). The data were predominantly collected at secondary, tertiary, or referral health-care centres; additional study-level details and relevant references are available in the appendix (pp 12–24). Although each study was required to include children with presumptive pulmonary tuberculosis, studies differed slightly with respect to inclusion criteria, variable definitions, and reference classification of tuberculosis (appendix pp 25–39). Of all 4718 children, 541 (11·5%) were classified as having confirmed tuberculosis, 1270 (26·9%) as having unconfirmed tuberculosis, and 2818 (59·7%) unlikely tuberculosis (appendix pp 40–65). Many demographic and clinical characteristics were similar between children belonging to these groups (appendix pp 66–67). All contributing studies had quality assessment scores of 4 out of 5 or 5 out of 5 (table 1; appendix p 68).

**Table 1:** Study-level descriptions of data included in the individual participant dataset

	**Sample size**	**Country**	**Age <2 years**	**Age <5 years**	**HIV**	**Severely acutely malnourished**	**Confirmed pulmonary tuberculosis**	**Unconfirmed pulmonary tuberculosis**	**Unlikely pulmonary tuberculosis**	Newcastle-Ottawa Scale*
Aurilio et al (2020)	50	Brazil	21 (42·0%)	31 (62·0%)	6 (12·0%)	0	9 (18·0%)	11 (22·0%)	24 (48·0%)	5
Giang et al (2015)	113	Viet Nam	86 (76·1%)	106 (93·8%)	0	8 (7·1%)	20 (17·7%)	77 (68·1%)	16 (14·2%)	5
Hamid et al (2019)	445	Pakistan	41 (9·2%)	175 (39·3%)	0	26 (5·8%)	0	29 (6·5%)	416 (93·5%)	5
Kabir et al (2020)	402	Bangladesh	219 (54·5%)	296 (73·6%)	0	93 (23·1%)	63 (15·7%)	36 (9·0%)	303 (75·4%)	4
López-Varela et al (2015)	789	Mozambique	549 (69·6%)	789 (100·0%)	104 (13·2%)	68 (8·6%)	13 (1·6%)	128 (16·2%)	648 (82·1%)	4
Marcy et al (2019)	338	Burkina Faso, Cambodia, Cameroon, and Viet Nam	78 (23·1%)	142 (42·0%)	338 (100%)	64 (18·9%)	41 (12·1%)	155 (45·9%)	142 (42·0%)	5
Myo et al (2018)	223	Myanmar	72 (32·3%)	150 (67·3%)	27 (12·1%)	46 (20·6%)	27 (12·1%)	84 (37·7%)	112 (50·2%)	5
Orikiriza et al (2018)	338	Uganda	124 (36·7%)	222 (65·7%)	101 (29·9%)	41 (12·1%)	12 (3·6%)	145 (42·9%)	167 (49·4%)	5
Orikiriza et al (2022)	217	Uganda	157 (72·4%)	196 (90·%)	70 (32·%)	108 (50·%)	12 (6·%)	58 (26·7%)	125 (57·6%)	4
Song et al (2021)	300	Kenya	146 (48·7%)	300 (100·0%)	73 (24·3%)	8 (2·7%)	31 (10·3%)	65 (22·3%)	170 (56·7%)	4
Valencia et al (2017)	142	Mozambique	59 (41·5%)	95 (66·9%)	70 (49·3%)	27 (19·0%)	5 (3·5%)	28 (19·7%)	109 (76·8%)	5
Walters et al (2017)	595	South Africa	389 (65·4%)	548 (92·1%)	70 (11·8%)	18 (3·0%)	119 (20·0%)	180 (30·3%)	283 (47·6%)	5
Zar et al (2019)	766	South Africa	362 (47·3%)	603 (78·7%)	137 (17·9%)	32 (4·2%)	189 (24·7%)	274 (35·8%)	303 (39·6%)	5
Total individual participant data	4718	..	2303 (48·8%)	3653 (77·4%)	996 (21·1%)	539 (11·4%)	541 (11·4%)	1270 (26·9%)	2818 (59·7%)	..

Additional study-level details and relevant references are available in the appendix (pp 12–24).

* Modified version for cohort studies; the highest score is 5, which indicates lower risk of bias.

**Figure 1:**
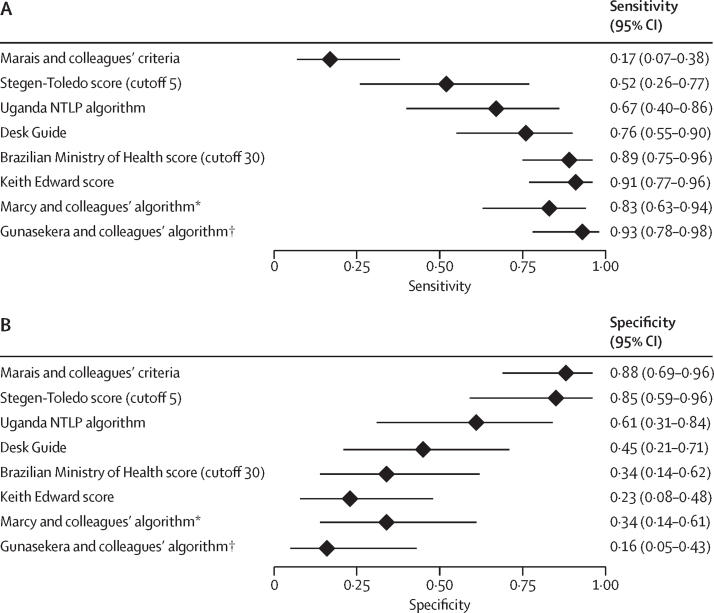
Performance of existing treatment-decision algorithms at classifying tuberculosis Retrospective estimates of the pooled sensitivity (A) and specificity (B) of eight algorithms to guide decisions to treat children with presumptive pulmonary tuberculosis, had they been used to evaluate the children for whom we have individual participant data records. The reference classification of pulmonary tuberculosis included bacteriologically confirmed pulmonary tuberculosis and unconfirmed pulmonary tuberculosis. Modifications were made to the algorithms to maximise the use of the available individual participant dataset. NTLP=National TB and Leprosy Program. *Performance estimates from Marcy and colleagues. The algorithm was derived from only HIV-positive children in the individual participant dataset that excludes data from the cohort comprising HIV-positive children from Burkina Faso, Cambodia, Cameroon, and Viet Nam (from which the algorithm was developed). †Performance estimates by Gunasekera and colleagues. The algorithm was derived from only HIV-negative children in the individual participant dataset that excludes data from the South Africa population (from which the algorithm was developed).

We evaluated the performance of eight existing treatment-decision algorithms. One of these algorithms was evaluated only on data from children living with HIV, and another evaluated only on data from children without HIV. Given that some algorithms considered variables that were not available in our individual participant dataset, we elected to estimate their performance using only the data that were available, without considering the contribution of those missing variables. References to each algorithm and details on which variables were not considered in this analysis are available in the appendix (pp 69–76). The sensitivities varied from 0·17 (95% CI 0·07–0·38) to 0·93 (0·78–0·98), with specificities varying from 0·88 (0·69–0·96) to 0·16 (0·05–0·43) when evaluated against the composite reference standard (figure 1; appendix pp 77–84). A sensitivity analysis evaluating performance to discriminate confirmed tuberculosis from unlikely tuberculosis showed marginally higher sensitivities and similar specificities to the performance in the entire dataset (appendix p 85).

ORs and 95% CIs of the predictors included in the model are shown in table 2. For the prediction model including chest x-ray features, the pooled *c* statistic was 0·71 (95% CI 0·66 to 0·76), the calibration intercept was –0·18 (–0·76 to 0·41), and the O:E ratio was 0·90 (0·64 to 1·26). Additional internal–external cross-validation *c* statistic, calibration intercept, and O:E ratio estimates are included in the appendix (pp 86–87). For the prediction model without chest x-ray features, the pooled *c* statistic was 0·65 (95% CI 0·59 to 0·71), the calibration intercept was –0·14 (–0·74 to 0·46), and the O:E ratio was 0·92 (0·60 to 1·43), with additional estimates included in the appendix (pp 88–90).

**Table 2:** Estimates from logistic regression prediction model to classify pulmonary tuberculosis using variables from initial evaluation

	**Coefficient (95% CI)**	**Odds ratio (95% CI)**	p value*
Intercept	−1·92 (−2·58 to −1·25)	..	..
Cough duration ≥2 weeks†	0·17 (−0·09 to 0·43)	1·19 (0·91 to 1·54)	0·86
Fever duration ≥2 weeks‡	0·45 (0·16 to 0·74)	1·57 (1·18 to 2·09)	0·25
Lethargy	0·25 (0·02 to 0·48)	1·28 (1·02 to 1·62)	0·66
Weight loss	0·22 (−0·03 to 0·48)	1·25 (0·97 to 1·62)	0·75
History of documented tuberculosis exposure	1·43 (0·87 to 2·00)	4·20 (2·39 to 7·38)	<0·0001
Haemoptysis	0·34 (−0·37 to 1·05)	1·40 (0·69 to 2·86)	0·78
Night sweats	0·20 (0·02 to 0·38)	1·22 (1·02 to 1·47)	0·71
Peripheral lymphadenopathy	0·35 (0·13 to 0·57)	1·42 (1·14 to 1·77)	0·35
Temperature ≥38°C	0·00 (−0·25 to 0·26)	1·00 (0·78 to 1·30)	>0·999
Tachycardia	0·15 (−0·13 to 0·42)	1·16 (0·88 to 1·53)	0·90
Tachypnoea	−0·05 (−0·27 to 0·16)	0·95 (0·77 to 1·18)	0·98
Cavities on chest x-ray	0·47 (−0·11 to 1·05)	1·60 (0·90 to 2·85)	0·53
Intrathoracic lymphadenopathy on chest x-ray	1·46 (1·00 to 1·92)	4·32 (2·73 to 6·85)	<0·0001
Opacities on chest x-ray	0·43 (0·02 to 0·84)	1·54 (1·02 to 2·32)	0·45
Miliary infiltrate on chest x-ray	1·27 (0·57 to 1·97)	3·56 (1·76 to 7·19)	0·0002
Pleural effusion on chest x-ray	0·64 (0·20 to 1·09)	1·90 (1·22 to 2·96)	0·13

The estimate provided for each predictor is computed against a reference that reflects the absence of that feature.

* Calculated using Rubin's rules for multiple imputed data.

† Absence is no cough or cough lasting less than 2 weeks.

‡ Absence is no fever or fever lasting less than 2 weeks.

The appendix shows the scores derived from the model prediction coefficients that correspond to classification of all tuberculosis with respective sensitivities of 90%, 85%, 80%, 75%, and 70% (pp 91–92), and the study-level and summary performance of these scores in classifying tuberculosis (pp 93–97).

**Figure 2:**
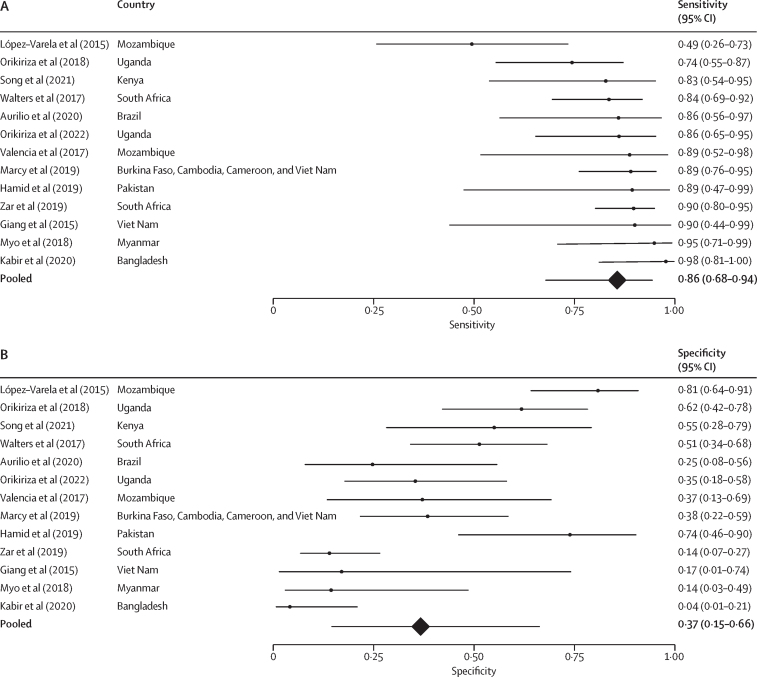
Forest plot depicting performance of scaled scores from prediction model to classify tuberculosis with 85% sensitivity Study-level and pooled estimates of the (A) sensitivity and (B) specificity of classifying tuberculosis (composite reference standard: bacteriologically confirmed pulmonary tuberculosis and unconfirmed pulmonary tuberculosis) of the scores derived from the prediction model developed from the individual participant dataset to classify tuberculosis with 85% sensitivity.

To balance the consequences of untreated tuberculosis versus the consequences of overtreatment, the expert group recommended a sensitivity threshold of 85% in classifying tuberculosis using the composite reference standard, resulting in the development of a score with a sensitivity of 0·86 (95% CI 0·68–0·94) and a specificity of 0·37 (0·15–0·66; figure 2). An analysis of the performance in classifying confirmed tuberculosis versus unlikely tuberculosis showed a sensitivity of 0·88 (95% CI 0·71–0·95) and specificity of 0·37 (0·15–0·67; appendix p 98). Under a sensitivity threshold of 85%, the model that included only features from the baseline clinical evaluation (without chest x-ray findings) had a sensitivity of 0·84 (95% CI 0·66–0·93) and specificity of 0·30 (0·13–0·56) in classifying tuberculosis (appendix pp 99–100).

To adapt the scores into treatment-decision algorithms to be used at primary health-care centres, the expert group recommended the following triage steps before tuberculosis classification using the score: identifying children with clinical symptoms and signs requiring urgent referral to higher levels of health care, and stratifying children by risk of mortality and progression of tuberculosis. Children at higher risk were defined by the expert group as those who belonged to any of the following categories: younger than 2 years, severely malnourished, or living with HIV. These children would be evaluated using the score at the time of the initial evaluation. Children not meeting this definition would be treated for the most likely non-tuberculosis condition and complete re-evaluation in 1–2 weeks; those with persistent or worsening symptoms at follow-up would be evaluated using the score. The expert group additionally recommended to pursue bacteriological testing, whenever available, on respiratory or stool specimens with rapid molecular diagnostics for all children and urine lateral flow assays for HIV-positive children, to align with existing WHO recommendations.^24^

**Figure 3:**
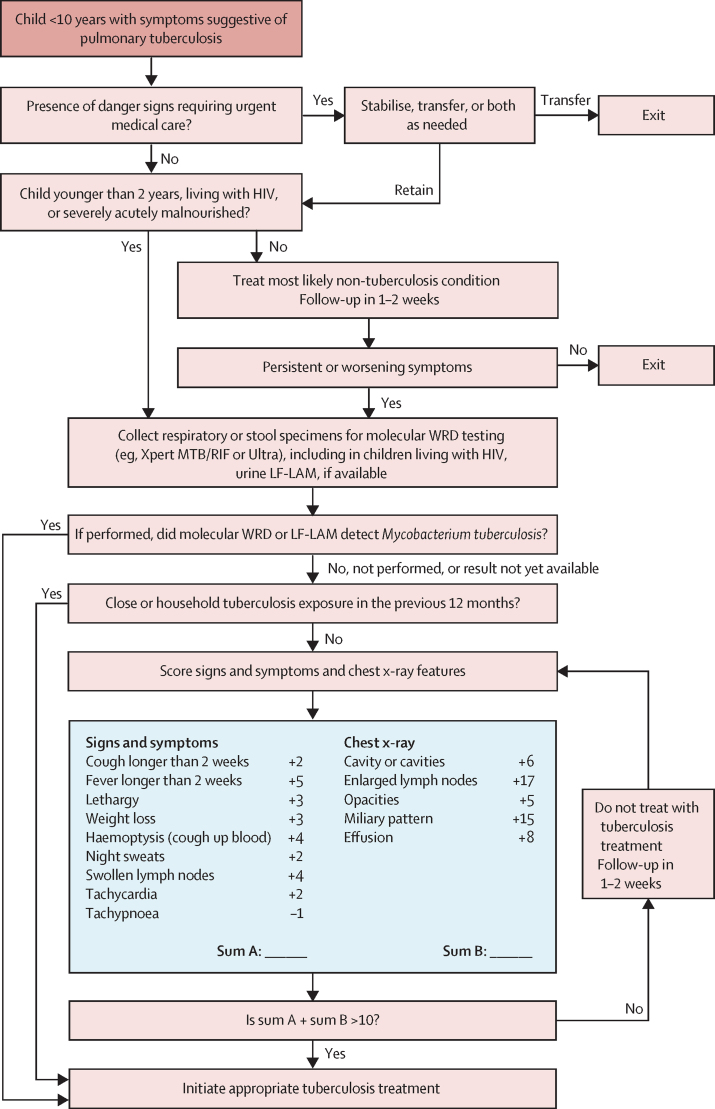
Treatment-decision algorithm including chest x-ray features derived from the prediction model Tuberculosis treatment-decision algorithm for use among children younger than 10 years with symptoms suggestive of pulmonary tuberculosis, reproduced from the operational handbook accompanying the WHO consolidated guidelines on the management of tuberculosis in children and adolescents.^12^, ^13^ Selection steps before entering the scoring system reflect recommendations from the WHO expert panel to enrich the probability of tuberculosis among the population of children proceeding through the algorithm to the model such that the probability would more closely reflect the preselected population producing the data from which the prediction model was built, while balancing the consequences of untreated tuberculosis in children at high risk. Scores associated with features from clinical history and physical exam and chest x-ray translate to risk of tuberculosis and are scaled from the prediction model developed from the individual participant dataset. Guidance on the practical use of this algorithm is outlined in the WHO operational handbook.^13^ LF-LAM=lateral flow urine lipoarabinomannan assay. WRD=WHO-recommended rapid diagnostic test.

The expert group recommendations resulted in the development of a treatment-decision algorithm (figure 3), in which children younger than 10 years with presumptive pulmonary tuberculosis are triaged by risk of tuberculosis-related morbidity and mortality before being evaluated for the presence of clinical and chest x-ray features to assign a score corresponding to tuberculosis risk. A total score of more than 10 results in classification of tuberculosis with a sensitivity of 85%. A known exposure to tuberculosis alone has a score of more than 10, which provides sufficient evidence to justify sensitive treatment decision making on its own; thus, this feature was placed above the other scored elements in the algorithm. The same parameters were used to construct the treatment-decision algorithm from the model without chest x-ray features (appendix p 101).

## Discussion

We assembled a large individual participant dataset from nearly 5000 children from geographically diverse, high tuberculosis incidence settings to evaluate existing treatment-decision algorithms and develop new evidence-based treatment-decision algorithms to guide evaluation of children with presumptive pulmonary tuberculosis. As far as we are aware, this work describes the largest analysis to date of the best currently available individual participant data to provide practical guidance to health-care workers in primary health-care settings to identify which clinical features, with or without chest x-ray assessment, indicate whether initiation of tuberculosis treatment is warranted.

Previous work in childhood tuberculosis algorithm development has been from smaller studies with limited investigation into generalisability. This analysis leverages the clustered nature of the individual participant dataset using an internal–external cross-validation framework to allow for more generalisable model parameter estimation and investigation into model validity. Furthermore, the algorithms were developed closely with members of the WHO and experts in childhood tuberculosis to prioritise sensitive treatment decision making to address the global burden of child mortality associated with untreated childhood tuberculosis. The newly developed algorithms were incorporated into the WHO operational handbook to support implementation of the new consolidated guidelines.^12^, ^13^

Of the clinical features, only reported exposure to tuberculosis was independently sufficient to meet the threshold for treatment initiation. This was true even in the model-based score without chest x-ray features, suggesting that none of the common clinical features could independently inform highly sensitive and specific treatment decisions. While results from tuberculin skin testing were used by studies to inform classification of tuberculosis and to improve imputation of missing data, we did not include these in the algorithms given operational limitations in using tuberculin skin testing at scale in high-burden settings. Of the chest x-ray features included in the algorithm, the presence of intrathoracic lymphadenopathy and a miliary pattern, respectively, were independently sufficient to start treatment. It is worth noting that inclusion of chest x-ray features only increased the specificity of the score slightly as compared with the score developed from the model with clinical features only. Chest x-ray has additional utility in guiding childhood pulmonary tuberculosis treatment duration for severe versus non-severe disease,^25^ in monitoring tuberculosis treatment response (including associated complications and sequelae), and in the diagnosis of other non-tuberculosis intrathoracic pathology.

The decision to prioritise sensitivity in our algorithm development is crucial to initiate appropriate treatment in more children with tuberculosis; however, many children might be incorrectly treated for tuberculosis given the resulting specificity. No test or algorithm meets the WHO target sensitivity and specificity for a confirmatory diagnostic test for childhood pulmonary tuberculosis.^26^ Thus, the expert group advised to develop an algorithm with a minimum sensitivity target of 85% as an acceptable balance between sensitivity and the resulting specificity. Given the estimated specificity of 37% for the scored part of the algorithm, it is likely that children without tuberculosis will be started on 4–6 months of treatment for tuberculosis and exposed to risk of adverse drug events. However, given the severe consequences of a missed tuberculosis diagnosis and the low rates of severe adverse drug events in this age group,^27^ this trade-off of improved sensitivity for poorer specificity is reasonable. Additionally, it is noteworthy that current WHO recommendations for preventive treatment include at a minimum 3–4 months of treatment. It is also true that overtreatment for tuberculosis might result in delayed diagnosis of non-tuberculosis disease. Future study of the relative cost of false positive versus false negative classification at varying prevalence of tuberculosis could inform sensitivity threshold selection in subsequent algorithm development.

We note that the model-based scoring component of the algorithm demonstrates considerable study-level heterogeneity in sensitivity and specificity. Although this individual participant dataset is the largest of its size compiled to date, there were not enough studies to quantitatively describe the features that drive the observed heterogeneity. Given that we used data made available to WHO following a public call rather than conducting a systematic review, it is possible that some diagnostic studies might have been excluded. The inclusion of more data from existing, ongoing, and future studies could allow meta-regression to describe study-level sources of heterogeneity. Heterogeneity might have been driven in part by varied tuberculosis prevalence in the cohorts included as well as heterogeneities in disease presentation.

Several existing algorithms evaluated have similar performance to ours. Although we are unable to formally compare our newly developed algorithm with existing algorithms, the similar performance suggests that there might be several algorithms that a public health programme could consider to suit specific settings, available resources, and other implementation considerations. Future analysis of available data, including those obtained through a systematic review, might provide the opportunity to revise and calibrate the model and further interrogate possible sources of heterogeneity. This could lead to investigation and development of algorithms that might perform better among different subgroups of children, including those at higher risk for tuberculosis-associated mortality.

We considered it important to evaluate existing treatment-decision algorithms and develop new algorithms using a composite standard rather than solely a microbiological standard, given the high proportion of children treated for tuberculosis without bacteriological confirmation, even in the best resourced settings, which reflects the paucibacillary nature of disease in most young children. However, this reference standard remains imperfect, and misclassification might occur.^28^ The underlying composition of the unconfirmed tuberculosis group might represent a heterogeneous group in which some children have tuberculosis, and some have other causes for their observed symptoms and signs. Additionally, it is possible that inclusion of unconfirmed pulmonary tuberculosis biased the estimation of the prediction model parameters, especially those used to classify the unconfirmed group. Although this is a limitation of our study, the similar performance estimates of the score developed in the primary analysis using both the composite and confirmed tuberculosis reference standards suggest that this might not be a major issue.

Given that our algorithms are intended to guide decisions to treat children in primary health-care centres, it is a limitation that our individual participant dataset was derived from primarily tertiary and referral health centres. We are not aware of studies that provide this quality of diagnostic evaluation data from presumptive childhood tuberculosis in primary health-care centres. However, in several studies, children presenting at primary health-care settings were directly referred for study evaluation, providing some degree of reassurance as to the generalisability of results. The pre-test probability of tuberculosis is likely to be substantially lower among children attending primary health-care centres and the clinical presentation might be different as compared with tertiary and referral centres from which the data were obtained due to differences in tuberculosis prevalence. These are important given that many children with tuberculosis first present to primary health-care centres.^29^ We believe that the risk stratification and delayed entry of lower risk children with presumptive tuberculosis (who should be able to tolerate the delay) is a practical attempt to safely raise the pre-test probability when implementing the algorithm in primary health-care centres.

It should be noted that although these performance estimates relate to the scored component of the algorithm, the overall sensitivity and specificity of the whole algorithm, which includes the triage steps, remain unknown and should be evaluated prospectively. As low-risk children are made to wait before being evaluated with the scored part of the algorithm, symptoms in some with diagnoses other than tuberculosis will resolve, probably improving specificity. Prospective evaluation of the entire algorithms in primary health-care settings will be crucial to determining their utility in improving case finding and reducing the mortality associated with untreated tuberculosis. Prospective studies of algorithm acceptability and feasibility are also indicated.

There are inherent limitations to developing a prediction model using data from multiple cohorts for a disease with an imperfect diagnostic gold standard. Study-level inclusion criteria varied, which affects the baseline tuberculosis prevalence and applicability of the score prediction estimates. Additionally, prediction variable definitions varied among the included studies—eg, history of weight loss was variably defined as caregiver-reported history of weight loss, objective weight loss, or deviation from previous growth trajectory. This heterogeneity is also true for the study-level reference classifications, especially for unconfirmed tuberculosis. Some studies used a previous version of the NIH reference classification, which included probable and possible tuberculosis categories that we reclassified as unconfirmed tuberculosis, despite the limitations of using this approach.^30^ Furthermore, studies contributing chest x-ray data included interpretations of managing health-care providers or expert radiologists, depending on the study setting. These might contribute to heterogeneities in estimating the association between the predictors and the outcome of tuberculosis. Notably, a high degree of missingness in the individual participant dataset limited the variables available to evaluate existing algorithms and include in algorithm development. Protocol standardisation for childhood tuberculosis diagnostic evaluation will reduce heterogeneity in variable definition and assist future attempts to consolidate data for algorithm development and evaluation. Finally, we note that using a prespecified prediction model, as we did, might lead to overfitting.^31^ Despite a reasonable summary O:E ratio for our model, the heterogeneity in study-level O:E ratio demonstrated in our internal–external cross-validation suggests that overfitting might be an issue. As more data become available, future investigation into the causes driving heterogeneity as well as other methods of prediction model feature selection might inform more nuanced use of this algorithm within specific contexts and populations.

Pragmatic treatment-decision algorithms can lead to better detection of tuberculosis in children, with improved access to early treatment and reduced tuberculosis morbidity and mortality. Although we developed these algorithms using a thorough modelling analysis of a large, high-quality individual participant dataset, the disappointing specificity of the scoring component suggests that improved diagnostic tools, such as computer-assisted interpretation of chest x-ray and biomarkers specific to tuberculosis, will be necessary to meet sensitivity and specificity targets. As these diagnostic tools become available, their data might be incorporated into treatment-decision algorithms to improve the specificity of the algorithms while maintaining high sensitivity.

Treatment-decision algorithms are now conditionally recommended by WHO in the evaluation of children with presumptive tuberculosis, which could lead to improved diagnostic capacity and treatment initiation at primary health-care centres where childhood tuberculosis expertise might be lacking. This work represents a paradigm shift in pragmatic and evidence-based approaches using advanced analytical methods to develop algorithms that draw on the best globally available data. This approach can be further improved and interrogated as additional data and diagnostic tools become available.

## Data sharing

Data are available upon written request to the corresponding author.

## Declaration of interests

We declare no competing interests.
