# Supplementary material for: Development of treatment-decision algorithms for children evaluated for pulmonary tuberculosis: an individual participant data meta-analysis
Source: Lancet Child Adolesc Health. 2023 May;7(5):336–46. doi: 10.1016/S2352-4642(23)00004-4 (PMC10127218; doi:10.1016/S2352-4642(23)00004-4)
Supplement: Supplementary appendix [file mmc1.pdf]

THE LANCET
Child & Adolescent Health

Supplementary appendix

This appendix formed part of the original submission and has been peer reviewed. We post it as supplied by the authors.

Supplement to: Gunasekera KS, Marcy O, Muñoz J, et al. Development of treatment-decision algorithms for children evaluated for pulmonary tuberculosis: an individual participant data meta-analysis. *Lancet Child Adolesc Health* 2023; published online March 13. https://doi.org/10.1016/S2352-4642(23)00004-4.

Supplement to **Development of treatment-decision algorithms for children being evaluated for pulmonary tuberculosis: an individual participant data meta-analysis**

Contents

Abbreviations and shorthand 2

Appendix A: Data requested 3

Appendix B: Information about multiple imputation by chained equations 7

Appendix C: Estimation of algorithm performance accounting for multiply imputed data 8

Appendix D: Develop a score from models produced on multiply imputed data 9

Appendix E: Composition of group of experts advising algorithm development 10

Appendix F: Studies involved and data contributed to IPD. 11

Appendix G: Study information 12

Appendix H: Modifications to IPD received 25

Appendix I: Missingness in IPD received 38

Appendix J: Generate additional variables 39

Appendix K: Descriptions of study-level IPD 40

Appendix L: Descriptions of entire IPD 66

Appendix M: Study quality assessment 68

Appendix N: Existing algorithms and modifications to make maximal use of IPD 69

Appendix O: Performance of existing algorithms against reference classification of all TB 77

Appendix P: Performance of existing algorithms against reference classification of bacteriologically-confirmed TB 85

Appendix Q: Discrimination and calibration of prediction model to classify TB 86

Appendix R: Logistic regression model, calibration, and discrimination of prediction model developed from IPD without CXR features 88

Appendix S: Prediction model fit and scaled scores at different sensitivity thresholds 91

Appendix T: Performance of scores from prediction model at different sensitivity thresholds 93

Appendix U: Performance of scores to classify TB with 85% sensitivity using confirmed TB reference standard 98

Appendix V: Performance of scores to classify TB with 85% sensitivity (without chest X-ray features) 99

Appendix W: Inspire algorithm without CXR features 101

Appendix X: TRIPOD Checklist 102

Appendix Y: Supplementary references 104

Abbreviations and shorthand

AUC – area under the receiver-operator curve
BCG - Bacille Calmette-Guérin vaccine
BD – Bangladesh
BMI – body mass index
BR – Brazil
CI – confidence interval
c-Statistic – concordance statistic
CXR – chest X-ray
EPTB – extrapulmonary tuberculosis
ES – expectorated sputum
GA – gastric aspirate
ART –Antiretroviral therapy
HIV – human immunodeficiency virus
IPD – individual participant data
IS – induced sputum
KE – Kenya
LF-LAM – lateral flow urine lipoarabinomannan assay
MICE – multiple imputation by chained equations
MM – Myanmar
MoH – Ministry of Health
Mtb – Mycobacterium tuberculosis
Multi – (PAANTHER) Multi-country study (includes Burkina Faso, Cameroon, Vietnam, and Cambodia)
mWRD – molecular WHO-recommended rapid diagnostic test
MZ – Mozambique
NTLP – National TB and Leprosy Program
O:E – observed: expected ratio
OR – odds ratio
PAANTHER - Pediatric Asian African Network for Tuberculosis and HIV Research
PK – Pakistan
PPD – purified protein derivative
PTB – pulmonary tuberculosis
SAM – severely acutely malnourished
TB – tuberculosis
TRIPOD - transparent reporting of a multivariable prediction model for individual prognosis or diagnosis
TST – tuberculin skin test
UG – Uganda
VN – Vietnam
WFAZ – weight-for-age Z-score
WFL – weight-for-length
WHO – World Health Organization
Xpert – Xpert MTB/RIF
Xpert Ultra – Xpert MTB/RIF Ultra
ZA – South Africa

Appendix A: Data requested

Table S1. Data requested from studies and suggested format

FIELD	VARIABLE	DESCRIPTION	FORMAT	CODE	LABEL
studyID	Study ID	Data source cohort	int	1	Aurilio/2020/BR
				2	Song/2021/KE
				3	LopezVarela/2015/MZ
				4	Valencia/2017/MZ
				5	Myo/2018/MM
				6	Marcy/2016/Multi
				7	Hamid/2019/PK
				8	Walters/2017/ZA
				9	Orikiriza/2018/UG
				10	Orikiriza/2021/UG
				11	Giang/2015/VN
				12	Kabir/2020/BD
				13	Zar/2019/ZA
age	Age (months)	Age (months) at enrolment	num	###	NA = unknown
sex	Sex	Participant sex	int	0	Female
				1	Male
				NA	Unknown
weight	Weight (kg)	Weight (kg) at initial evaluation	num	###	NA = unknown
height	Height (cm)	Height/length (cm) at initial evaluation	num	###	NA = unknown
bcg_evidence	BCG vaccination	Evidence of BCG vaccination (BCG scar or BCG recorded in immunization record) at initial evaluation	int	0	No evidence of BCG vaccination
				1	Evidence of BCG vaccination
				NA	Unknown
HIV_status	HIV status	Participant HIV status	int	0	HIV-negative
				1	HIV-positive
				NA	Unknown
cough_less2wk_gr2wk_gr3wk_gr4wk	Cough duration	Duration of cough at initial evaluation	int	0	No cough
				1	Cough 0-13 days
				2	Cough 14-20 days
				3	Cough 21-27 days
				4	Cough ≥28 days
				NA	Unknown
cough_greater_2wk	Cough duration	Presence of cough ≥2 weeks at initial evaluation	int	0	Cough ≥2 weeks not present
				1	Cough ≥2 weeks present
				NA	Unknown
fever_less2wk_gr2wk_gr3wk_gr4wk	Fever duration	Duration of fever at initial evaluation	int	0	No fever
				1	Fever 0-13 days
				2	Fever 14-20 days
				3	Fever 21-27 days
				4	Fever ≥28 days
				NA	Unknown
fever_greater_1wk	Fever duration	Presence of fever ≥1 week at initial evaluation	int	0	Fever ≥1 week not present
				1	Fever ≥1 week present
				NA	Unknown
lethargy	Lethargy	Presenting history of unusual lethargy or lack of playfulness at initial evaluation	int	0	No lethargy
				1	Lethargy

				NA	Unknown
weight_loss	Weight loss	Presenting history of poor growth over the preceding 3 months AND not responding to nutritional rehabilitation (or antiretroviral therapy if HIV infected)	int	0	No weight loss
				1	Weight loss
				NA	Unknown
significant_tbc	Documented TB exposure	Documented exposure to MTB at initial evaluation in previous 12 months	int	0	No documented TB exposure in previous 12 months
				1	Documented TB exposure in previous 12 months
				NA	Unknown
night_sweats	Night sweats	Presenting history of night sweats at initial evaluation	int	0	No night sweats
				1	Night sweats
				NA	Unknown
hemoptysis	Haemoptysis	Presenting history of haemoptysis at initial evaluation	int	0	No haemoptysis
				1	Haemoptysis
				NA	Unknown
temp	Temperature (C)	Recorded temperature at initial evaluation	num	###	NA=unknown
heart_rate	Heart rate (per min)	Heart rate (per minute) at initial evaluation	num	###	NA=unknown
respiratory_rate	Respiratory rate (per min)	Respiratory rate (per minute) at initial evaluation	num	###	NA=unknown
peripheral_lad	Peripheral lymphadenopathy	Peripheral lymphadenopathy (at cervical, submandibular, and/or axillary nodes) at initial evaluation	int	0	No peripheral lymphadenopathy
				1	Peripheral lymphadenopathy
				NA	Unknown
first_xpert_result	First Xpert MTB/RIF	Result from first Xpert MTB/RIF (not Ultra) performed on ES/IS (or GA for young children) collected at initial evaluation	int	0	Xpert negative for Mtb
				1	Xpert positive for Mtb
				NA	Unknown/not performed
CXRfinal_result	CXR consistent with TB	Result of CXR performed at initial evaluation as assessed by reader performing clinical evaluation/making TB-treatment decision or by reader to inform research classification of TB if former not available	int	0	CXR not consistent with TB
				1	CXR consistent with TB
				NA	Unknown/not assessed

CXRopacity_result	Opacities on CXR	Opacities (e.g., alveolar consolidation and/or bronchopneumonia) on CXR performed at initial evaluation as assessed by reader performing clinical evaluation/making TB-treatment decision or by reader to inform research classification of TB if former not available	int	0	Opacities not present on CXR
				1	Opacities present on CXR
				NA	Unknown/not assessed
CXRcavity_result	Cavities on CXR	Cavities on CXR performed at initial evaluation as assessed by reader performing clinical evaluation/making TB-treatment decision or by reader to inform research classification of TB if former not available	int	0	Cavities not present on CXR
				1	Cavities present on CXR
				NA	Unknown/not assessed
CXRmili_result	Miliary infiltrate on CXR	Miliary infiltrate on CXR performed at initial evaluation as assessed by reader performing clinical evaluation/making TB-treatment decision or by reader to inform research classification of TB if former not available	int	0	Miliary infiltrate not present on CXR
				1	Miliary infiltrate present on CXR
				NA	Unknown/not assessed
CXRnodes_result	Intrathoracic lymphadenopathy on CXR	Intrathoracic lymphadenopathy (e.g., perihilar nodes, paratracheal nodes, mediastinal nodes) on CXR performed at initial evaluation as assessed by reader performing clinical evaluation/making TB-treatment decision or by reader to inform research classification of TB if former not available	int	0	Intrathoracic lymphadenopathy not present on CXR
				1	Intrathoracic lymphadenopathy present on CXR
				NA	Unknown/not assessed
CXReffusion_result	Pleural effusion on CXR	Pleural effusion on CXR performed at initial evaluation as assessed by reader performing clinical evaluation/making TB-treatment decision or by reader to inform research classification of TB if former not available	int	0	Pleural effusion not present on CXR
				1	Pleural effusion present on CXR
				NA	Unknown/not assessed
TST_result	Tuberculin skin test	Tuberculin skin test positive at initial evaluation	num	0	TST negative
				1	TST positive
				NA	Unknown/not performed
TB_classification	TB classification	Final classification of TB	int	0	Unlikely TB

				1	Bacteriologically-confirmed TB
				2	Unconfirmed TB
				NA	Unknown

Appendix B: Information about multiple imputation by chained equations

Imputation of missing data was carried out using the multiple imputation by chained equations (MICE) methods implemented in the R package, *mice*.[1] MICE is a fully conditionally specified modeling approach that first imputes the mean for missing data in each variable, then uses regression modeling to re-impute missing data in each variable by conditioning on the remaining variables, and finally iteratively updates imputations using the newly imputed data.

All IPD specified in Table S1 were included in the imputation models. We included a cluster-specific, normally distributed random effects term in the imputation model for each variable whenever possible to allow for study-level heterogeneities in the baseline distribution for each imputed variable. For continuous variables and categorical variables, we used a two-level predictive mean matching model implemented using the "2l.pmm" method in the R package, *miceadds*.[2] For binary variables, we used a two-level logistic model implemented using the "2l.bin" method. Few binary variables gave a singular fit warnings using two-level methods; for these variables, we reflexed to using a one-level logistic model implemented using the "logreg" method. MICE was run using the "mice" function with 20 iterations to generate 100 imputed datasets. The methods used to impute each variable are specified as follows:

2l.pmm: cough_less2wk_gr2wk_gr3wk_gr4wk, fever_less2wk_gr2wk_gr3wk_gr4wk, weight, temp, heart_rate, respiratory_rate, height, TB_classification
2l.bin: cough_greater_2wk, fever_greater_1wk, lethargy, weight_loss, night_sweats, peripheral_lad, significant_tbc, bcg_evidence, HIV_status, CXRfinal_result, CXRnodes_result, CXRopacity_result, CXReffusion_result, TST_result, CXRcavity_result, first_xpert_result
logreg: sex, haemoptysis, CXRmili_result

Additional imputation specifications are as follows:

Cluster variable: studyID
No imputation method specified (fully complete data): studyID, age

Convergence of imputation was visually assessed by trace plots of the mean and standard deviation of each variable over the imputation iterations.

Appendix C: Estimation of algorithm performance accounting for multiply imputed data

For each algorithm, sensitivity and specificity estimates were computed at the study-level and pooled using a bivariate model as implemented in the "reitsma" function in the *mada* package in R.[3] To account for the uncertainty associated with missing data, we imputed 100 datasets as specified in Appendix B. Study-level and pooled estimates of sensitivity and specificity were computed for each of the 100 datasets. We pooled the sensitivity and specificity estimates computed at the level of each imputed dataset using Rubin's rules to yield a point estimate and 95% confidence intervals for the study-level and for the overall pooled estimates computed by the "reitsma" function.[4]

Appendix D: Develop a score from models produced on multiply imputed data

A general form of a multivariate logistic regression equation is given as follows:

$$logit(p) = \beta_0 + \beta_1 * x_1 + \beta_2 * x_2 + \cdots + \beta_n * x_n$$

Where p is the probability of tuberculosis, $x_{1 \ldots n}$ refers to the predictors and $\beta_{1 \ldots n}$ refers to the coefficients describing the relationship between the predictor and the logit-transformed probability. We fit the prediction model to the data, and we identified the probability corresponding to classification of tuberculosis with a given sensitivity compared to the reference standard. For example, let us specify an interest in classifying tuberculosis with a sensitivity of at least 85%. We obtained a threshold probability by subtracting the intercept from the logit-transformed probability corresponding to diagnosis with at least an 85% sensitivity. We scaled the threshold probability to 10 by multiplying by a scaling factor, and we multiplied the coefficients for each predictor by that scaling factor to obtain the score for that predictor. Thus, the score for each individual meeting entry criteria was obtained by summing the scaled coefficients for each factor present in the patient, and a total score of >10 constituted a diagnosis of tuberculosis with a sensitivity of 85% using this treatment-decision algorithm.

Given that we generated multiple imputed datasets, we had to take additional steps to determine the probability threshold and pooled coefficient estimates. We used the “metapred” function in package *metamisc* to fit a logistic regression model on each imputed dataset, resulting in 100 logistic regression models.[5] Note that the model generated by “metapred” is a pooled model of models with the same specifications fit at the study-level. A pooled estimate of each parameter coefficient was obtained by taking the mean of each of the 100 coefficient estimates for each parameter. A pooled probability threshold was determined by taking the mean of the probability threshold corresponding to classification of tuberculosis with a given sensitivity compared to the reference standard of each model. The pooled probability threshold and pooled coefficient estimates were used to produce the scores as described above.

Appendix E: Composition of group of experts advising algorithm development

- *Farhana Amanullah*: Indus Hospital & Health Network, Karachi, Pakistan
- *Annemieke Brands*: Global Tuberculosis Programme, World Health Organization, Geneva, Switzerland
- *Stephen Graham*: Centre for International Child Health, University of Melbourne Department of Paediatrics and Murdoch Children's Research Institute, Royal Children's Hospital, Melbourne, Australia and International Union Against Tuberculosis and Lung Disease (The Union), Paris, France
- *Kenneth Gunasekera*: Department of Epidemiology of Microbial Diseases, Yale School of Public Health, New Haven, United States of America
- *Alexander Kay*: Global TB Program, Baylor College of Medicine and Texas Children's Hospital, Houston, United States of America
- *Anna Mandalakas*: Global TB Program, Baylor College of Medicine and Texas Children's Hospital, Houston, United States of America
- *Ben Marais*: The Children's Hospital at Westmead Clinical School, Faculty of Medicine and Health, University of Sydney, Sydney Australia
- *Olivier Marcy*: University of Bordeaux, Inserm, Institut de Recherche pour le Développement, Bordeaux, France
- *James Seddon*: Desmond Tutu Tuberculosis Centre, Department of Paediatrics and Child Health, Faculty of Medicine and Health Sciences, Stellenbosch University, Tygerberg, South Africa and Department of Infectious Diseases, Imperial College London, London, United Kingdon
- *Moorine Sekkade*: National Tuberculosis and Leprosy Program, Kampala, Uganda
- *Sabine Verkuijl*: Global Tuberculosis Programme, World Health Organization, Geneva, Switzerland
- Kerri Viney Global Tuberculosis Programme, World Health Organization, Geneva, Switzerland and School of Public Health, University of Sydney, Sydney, Australia
- *Bryan Vonasek*: Department of Pediatrics, University of Wisconsin School of Medicine and Public Health, Madison, United States of America

Appendix F: Studies involved and data contributed to IPD.

Figure S1. Flow-diagram demonstrating how the eighteen studies that were identified as having potentially appropriate data for this analysis led to inclusion of 4,718 IPD records from children <10 years with presumptive pulmonary TB (1,811 [38·3%] were found to have either bacteriologically-confirmed TB or unconfirmed TB). *Data from three studies were not considered in this analysis for reflecting a population of children on treatment for TB (n=1) and active case finding populations (n=2). TB – tuberculosis, IPD – individual participant data, PTB – pulmonary tuberculosis.

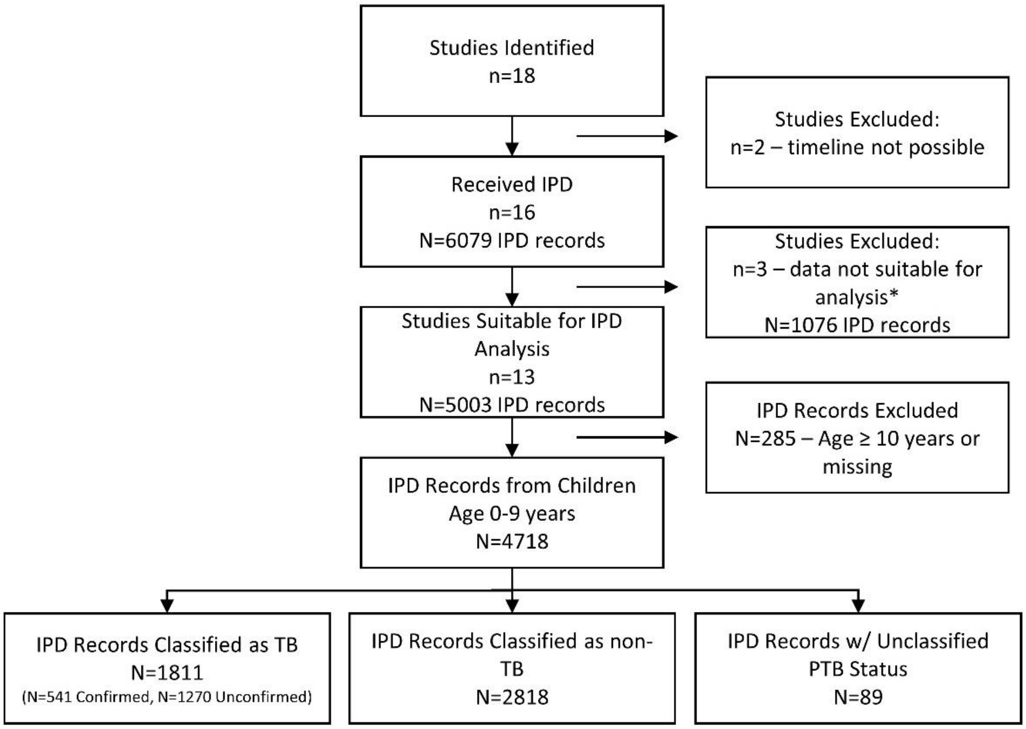

Appendix G: Study information

Table S2. Study information for Kabir/2020/BD

**Geographic setting**	Dhaka, Bangladesh		
**Representing co-authors (in no particular order)**	Sayera Banu, Senjuti Kabir, Shakil Ahmed		
**Healthcare setting**	**Name**	**Healthcare level**	**Recruitment setting**
	Dhaka Medical College Hospital	Tertiary	Inpatient
	Sir Salimullah Medical College and Mitford Hospital	Tertiary	Inpatient
	Shaheed Suhrawardy Medical College and Hospital	Tertiary	Inpatient
	icddr,b Dhaka Hospital	Tertiary	Inpatient
**Enrolment duration**	22 January 2018 – 04 April 2019		
**Purpose for data collection**	Evaluate the performance of Xpert MTB/RIF Ultra assay on stool specimen for the diagnosis of childhood pulmonary TB		
**Study design**	Cross-sectional study with follow-up of children diagnosed both bacteriologically and clinically at every month over phone started on antituberculosis therapy of 6 months		
**Inclusion criteria**	Children aged 0-<15 years with symptoms suggestive of pulmonary TB based on any of the following: persistent non-remitting cough for >14 days weeks not responding to antibiotics, persistent documented fever for >14 days, document weight loss or failure to gain weight over the preceding 3 months, or fatigue/reduced playfulness/decreased activity		
**Exclusion criteria**	Children with serious co-morbid condition (e.g., in intensive care unit, co-morbid heart condition, etc.); physician unable to collect respiratory specimen; children started on anti-tuberculosis treatment; children suspected clinically to have intestinal TB		
**Standardized form to guide evaluation?**	YES		
**Standardized form to guide CXR evaluation?**	NO: local treating physicians evaluated CXR		
**Standardized follow-up for all children?**	NO: only children diagnosed w/ TB and initiated on anti-tuberculosis treatment were followed-up over phone to assess symptom resolution. Moreover, five children with “trace call” results on Xpert Ultra using stool were not advised for anti-TB treatment by treating physicians and were followed-up over phone at six months to assess if they had developed TB.		
**Reference classification of TB**	Confirmed PTB – bacteriologically positive on culture/Xpert/Xpert Ultra on either induced sputum or stool specimens;<br>Unconfirmed PTB – bacteriologically negative but diagnosed by managing clinical team based on symptoms, CXR, TST, and contact history;<br>Unlikely PTB – not meeting criteria for confirmed TB or unconfirmed TB;<br>Classifications made by study team (separate from managing clinical team) based on data from the initial evaluation		
**No. screened/No. enrolled**	454/447		
**Ethics review**	Protocol no.PR#17072, Approved from Institutional Review Board, icddr,b constitutes of two Committee Research Review Committee on RRC on 18 July 2017 and Ethical Review Committee on 28 August 2017		
**Reference**	[6]		

Table S3. Study information for Aurilio/2020/BR

**Geographic setting**	Rio de Janeiro, Brazil		
**Representing co-authors (in no particular order)**	Clemax Couto Sant'Anna, Rafaela Baroni Aurilio		
**Healthcare setting**	**Name**	**Healthcare level**	**Recruitment setting**
	Instituto de Puericultura e Pediatria Martagao Gesteira	Tertiary	Inpatient/Outpatient
	Hospital Raphael de Paula Souza	Tertiary	Inpatient/Outpatient
	Hospital Universitário Antonio Pedro	Tertiary	Inpatient/Outpatient
**Enrolment duration**	17 April 2014 – 27 July 2020		
**Purpose for data collection**	Evaluate Xpert MTB/RIF as diagnostic test for PTB in children		
**Study design**	Prospective cohort study with baseline assessment and follow-up of all children at 60 days. Assessment of treatment outcome at 6 months or upon completion of treatment for those started on anti-TB treatment.		
**Inclusion criteria**	Children aged 0-19 years with symptoms of respiratory infection for ≥14 days and abnormal CR		
**Exclusion criteria**	Inappropriate samples for Xpert		
**Standardized form to guide evaluation?**	YES		
**Standardized form to guide CXR evaluation?**	YES		
**Standardized follow-up for all children?**	YES		
**Reference classification of TB**	Graham 2015: Confirmed PTB, Unconfirmed PTB, Unlikely PTB;[7] Retrospective classifications made by study team (separate from managing clinical team) at the 2-month follow-up visit		
**No. screened/No. enrolled**	50/50 (among those children <10 years old)		
**Ethics review**	Instituto de Puericultura e Pediatria Martagão Gesteira (24/02/2015, number 961·452 and 07/11/2017 number 2·369·814) and Hospital Universitário Antônio Pedro (28/07/2015 number 1·160·695)		
**Reference**	[8]		

Table S4. Study information for Song/2021/KE

**Geographic setting**	Kisumu County, Kenya		
**Representing co-authors (in no particular order)**	Jonathan P. Smith, Eleanor S. Click, Rinn Song, Kevin P. Cain		
**Healthcare setting**	**Name**	**Healthcare level**	**Recruitment setting**
	Jaramogi Oginga Odinga Teaching and Referral Hospital	Tertiary	Inpatient/Outpatient
	Additional patients from unspecified secondary inpatient/outpatient, and contact tracing		
**Enrolment duration**	October 2013 – August 2015		
**Purpose for data collection**	Determine the performance of a wide panel of specimen types and microbiological tests in children evaluated for TB		
**Study design**	Prospective cohort study with baseline assessment and follow-up of all children at 2 weeks, 2 months, and 6 months.		
**Inclusion criteria**	Children aged <5 years, who weighed >2·5 kg with 1) cervical lymphadenopathy despite antibiotics or 2) parenchymal abnormality on chest radiograph in addition to at least one of the following symptoms: 1) cough despite antibiotics, 2) malnutrition despite treatment for malnutrition, and 3) fever despite antibiotics or antimalarials.		
**Exclusion criteria**	Currently on anti-tuberculosis treatment or isoniazid preventive therapy or history of anti-tuberculosis treatment or isoniazid preventive therapy in the 6 months prior to enrolment.		
**Standardized form to guide evaluation?**	YES		
**Standardized form to guide CXR evaluation?**	YES		
**Standardized follow-up for all children?**	YES		
**Reference classification of TB**	Graham 2015: Confirmed PTB, Unconfirmed PTB, Unlikely PTB;[7]<br>Retrospective classifications made by study team (separate from managing clinical team) using information from all visits up to the 2-month follow-up		
**No. screened/No. enrolled**	2564/300		
**Ethics review**	US Centers for Disease Control and Prevention (#6334)<br>Kenya Medical Research Institute (#2343)<br>Jaramogi Oginga Odinga Teaching and Referral Hospital<br>Children's Hospital Boston/Harvard Medical School (relied on the review and oversight of the Centers for Disease Control and Prevention institutional review board)		
**Reference**	[9]		

Table S5. Study information for LopezVarela/2015/MZ

<table>
<tr><td>Geographic setting</td><td colspan="3">Manhiça District, Mozambique</td></tr>
<tr><td>Representing co-authors (in no particular order)</td><td colspan="3">Elisa Lopez-Varela, Orvalho Augusto</td></tr>
<tr><td rowspan="3">Healthcare setting</td><td>Name</td><td>Healthcare level</td><td>Recruitment setting</td></tr>
<tr><td>Manhiça District Hospital (and attached health centre)</td><td>Secondary</td><td>Inpatient/Outpatient</td></tr>
<tr><td colspan="3">*1483 patients from Manhiça District Hospital and Manhiça Health Research Centre Health and Demographic Surveillance System peripheral health centres (Palmeira, Maragra, Ilha, Josina, Taninga) and 180 contacts through contact tracing</td></tr>
<tr><td>Enrolment duration</td><td colspan="3">2011 - 2012</td></tr>
<tr><td>Purpose for data collection</td><td colspan="3">Estimate the annual minimum incidence of TB in children <3 in the Manhiça District</td></tr>
<tr><td>Study design</td><td colspan="3">Prospective cohort study with baseline assessment and follow-up of all children within 6 months of enrolment. Persistently symptomatic children had additional evaluation and testing.</td></tr>
<tr><td>Inclusion criteria</td><td colspan="3">Children aged 0-3 years with symptoms suggestive of PTB or EPTB or who are close contacts of notified TB cases. Symptoms suggestive of PTB included one or more of the following: cough ≥14 days not responding to appropriate antibiotics, fever ≥14 days after excluding malaria/pneumonia, chronic or acute malnutrition or failure to gain weight for more than 2 months, unexplained wheeze ≥14 months not responding to treatment, lower respiratory tract infection ≥14 days not responding to antibiotics after 72 hours, contact with TB case in previous 12 months)</td></tr>
<tr><td>Exclusion criteria</td><td colspan="3">Children aged >3 who reside outside the study area or with diagnosis of TB at pre-enrolment</td></tr>
<tr><td>Standardized form to guide evaluation?</td><td colspan="3">YES</td></tr>
<tr><td>Standardized form to guide CXR evaluation?</td><td colspan="3">YES</td></tr>
<tr><td>Standardized follow-up for all children?</td><td colspan="3">NO</td></tr>
<tr><td>Reference classification of TB</td><td colspan="3">Graham 2012: Confirmed PTB, Probable PTB, Possible PTB, PTB Unlikely, MTB infection;[10]
Classification made by the managing clinical team using information from the baseline visit and all available follow-up visits</td></tr>
<tr><td>No. screened/No. enrolled</td><td colspan="3">1663/789</td></tr>
<tr><td>Ethics review</td><td colspan="3">The study protocol was approved by the Mozambican National Bioethics Committee and the Hospital Clinic of Barcelona Ethics Review Committee.</td></tr>
<tr><td>Reference</td><td colspan="3">[11]</td></tr>
</table>

Table S6. Study information for Valencia/2017/MZ

**Geographic setting**	Manhiça District, Mozambique		
**Representing co-authors (in no particular order)**	Alberto L. García-Basteiro, Lucía Carratalá-Castro		
**Healthcare setting**	**Name**	**Healthcare level**	**Recruitment setting**
	Manhiça District Hospital (and attached health centre)	Secondary	Inpatient/Outpatient
	*Manhiça Health Care Centre and Manhiça District Hospital and 9 peripheral health care centre (Maluana, Munguine, Taninga, Maragra, Malavela, Palmeiras, Chibututuine, Calanga, Chibucutzo)		
**Enrolment duration**	20 August 2013 – 20 August 2014		
**Purpose for data collection**	Improve the quality of TB surveillance indicators using newly introduced Xpert MTB/RIF		
**Study design**	Cross-sectional study with baseline assessment. Digital CXR only if clinician ordered. Two-week follow-up for children not initially started on TB treatment. Follow-up of all children started on TB treatment at months 2 and 6.		
**Inclusion criteria**	Children and adults with symptoms suggestive of PTB or EPTB or who are close contacts of notified TB cases. Symptoms suggestive of PTB include cough ≥2 weeks, night sweats, weight loss, fever, and/or haemoptysis.		
**Exclusion criteria**	Children diagnosed with TB prior to enrolment.		
**Standardized form to guide evaluation?**	YES		
**Standardized form to guide CXR evaluation?**	YES		
**Standardized follow-up for all children?**	NO		
**Reference classification of TB**	Confirmed PTB – Bacteriologically positive on culture/Xpert on any respiratory specimen;<br>Unconfirmed PTB - bacteriologically negative but diagnosed by managing clinical team based on symptoms, CXR, TST, and contact history;<br>Unlikely PTB - not meeting criteria for confirmed TB or unconfirmed TB;<br>Classification made by the managing clinical team using information from the initial visit and information gathered on follow-up at 2-weeks for those not started on TB treatment and after first few weeks of follow-up for those started on TB treatment		
**No. screened/No. enrolled**	NA/142		
**Ethics review**	The study was approved by CISM local bioethics committee (CIBS) and the National Bioethics Committee (CNBS). Ref. 199/CNBS13		
**Reference**	[12]		

Table S7. Study information for Myo/2018/MM

<table>
<tr><td>Geographic setting</td><td colspan="3">Mandalay, Myanmar</td></tr>
<tr><td>Representing co-authors (in no particular order)</td><td colspan="3">Aye Aye Myint, Kyaw Myo</td></tr>
<tr><td rowspan="2">Healthcare setting</td><td>Name</td><td>Healthcare level</td><td>Recruitment setting</td></tr>
<tr><td>Children Hospital, Mandalay</td><td>Tertiary</td><td>Inpatient and Casualty/Emergency</td></tr>
<tr><td>Enrolment duration</td><td colspan="3">01 January 2015 – 21 March 2017</td></tr>
<tr><td>Purpose for data collection</td><td colspan="3">Evaluate Xpert MTB/RIF as diagnostic test for PTB in children</td></tr>
<tr><td>Study design</td><td colspan="3">Prospective cohort study with baseline assessment and 8-week follow-up.</td></tr>
<tr><td>Inclusion criteria</td><td colspan="3">Children aged 0-12 years with cough ≥14 days and one of the following: fever >7 days, weight loss or failure to thrive, unexplained loss of appetite, or lethargy.</td></tr>
<tr><td>Exclusion criteria</td><td colspan="3">Receipt of anti-tuberculosis treatment for >72 hours before specimen collection.</td></tr>
<tr><td>Standardized form to guide evaluation?</td><td colspan="3">YES</td></tr>
<tr><td>Standardized form to guide CXR evaluation?</td><td colspan="3">YES</td></tr>
<tr><td>Standardized follow-up for all children?</td><td colspan="3">YES</td></tr>
<tr><td>Reference classification of TB</td><td colspan="3">Graham 2015: Confirmed PTB, Unconfirmed PTB, Unlikely PTB;
Retrospective classifications made by study team (separate from managing clinical team) at the 2-month follow-up visit</td></tr>
<tr><td>No. screened/No. enrolled</td><td colspan="3">259/255</td></tr>
<tr><td>Ethics review</td><td colspan="3">Research Ethics Committee, University of Medicine, Mandalay</td></tr>
<tr><td>Reference</td><td colspan="3">[13]</td></tr>
</table>

Table S8. Study information for Marcy/2019/Multi

**Geographic setting**	Bobo Dioulasso, Burkina Faso<br>Phnom Penh, Cambodia<br>Siem Reap, Cambodia<br>Yaounde, Cameroon<br>Ho Chi Minh City, Vietnam		
**Representing co-authors (in no particular order)**	Olivier Marcy, Vibol Ung		
**Healthcare setting**	**Name**	**Healthcare level**	**Recruitment setting**
	Pediatric Department, Centre Hospitalier Universitaire Souro Sanou, (Bobo Dioulasso, Burkina Faso)	Tertiary	Inpatient/Outpatient
	National Pediatric Hospital (Phnom Penh, Cambodia)	Tertiary	Inpatient/Outpatient
	Angkor Hospital for Children (Siem Reap, Cambodia)	Tertiary	Inpatient/Outpatient
	Centre Hospitalier de la Caisse d'Essos (Yaounde, Cameroon)	Secondary	Inpatient/Outpatient
	Centre Mère et Enfant de la Fondation Chantal Biya (Yaounde, Cameroon)	Secondary	Inpatient/Outpatient
	Pediatric Department, Pham Ngoc Thach Hospital (Ho Chi Minh City, Vietnam)	Tertiary	Inpatient/Outpatient
	Infectious Diseases Department, Pediatric Hospital No. 1 (Nhi Dong 1) (Ho Chi Minh City, Vietnam)	Tertiary	Inpatient/Outpatient
	Infectious Diseases Department, Pediatric Hospital No. 2 (Nhi Dong 2) (Ho Chi Minh City, Vietnam)	Tertiary	Inpatient/Outpatient
**Enrolment duration**	April 2011 – December 2014		
**Purpose for data collection**	Evaluate Xpert MTB/RIF performed on stool for Mtb and to assess response to antituberculosis treatment for children living with HIV		
**Study design**	Prospective cohort study with baseline assessment and follow-up of all children at months 1,2,3, and 6.		
**Inclusion criteria**	Children aged 0-12 years with HIV-1 infection (irrespective of HAART) and one or more of the following: cough >14 days, fever > 14 days, failure to thrive (deviation from previous growth trajectory in previous 3 months or weight-for-age Z-score <-2), failure to improve on broad spectrum antibiotics for pulmonary infection, or CXR suggestive of PTB		
**Exclusion criteria**	History of any anti-tuberculosis treatment in the 2 years prior to enrolment.		
**Standardized form to guide evaluation?**	YES		
**Standardized form to guide CXR evaluation?**	YES		
**Standardized follow-up for all children?**	YES		
**Reference classification of TB**	Graham 2015: Confirmed PTB, Unconfirmed PTB, Unlikely PTB;<br>Retrospective classifications made by an algorithm following the Graham 2015 classification using information from all visits up to the 6-month follow-up visit		
**No. screened/No. enrolled**	NA/438		
**Ethics review**	Ethics Committee for Research in Health (Burkina Faso);<br>National Ethics Comity for Health and Research (Phnom Penh, Cambodia);<br>National Ethics Committee (Cameroon);<br>Division of Health Operations Research Ministry of Public Health (Cameroon);<br>Pham Ngoc Thach Hospital Institutional Review Board (Vietnam);<br>Ho Chi Minh City Department of Health (Vietnam);<br>Ho Chi Minh City People's Committee (Vietnam).		
**Reference**	[14]		

Table S9. Study information for Hamid/2019/PK

**Geographic setting**	Karachi, Pakistan		
**Representing co-authors (in no particular order)**	Iraj Batool, Aliya Anwar, Sara Ahmed Siddiqui, Farhana Amanullah		
**Healthcare setting**	**Name**	**Healthcare level**	**Recruitment setting**
	**The** Indus Hospital Ghauri Clinic	Tertiary/Referral	Outpatient
	Participants were referred from the TB screening/ contact tracing program as well as from community general physicians, and family physicians/pediatricians/ surgeons of the Indus hospital.		
**Enrolment duration**	01 January 2019 – 06 April 2020		
**Purpose for data collection**	**To** identify gaps in child TB care delivery and improve Pediatric TB Program implementation		
**Study design**	Cross-sectional study with baseline assessment and 1-month follow-up		
**Inclusion criteria**	Children aged 0-10 years with any of the following: 2 symptoms of TB (cough ≥14 days, fever, weight loss, lethargy, loss of appetite, night sweats), a known TB exposure within the past 2 years with ≥1 symptom suggestive of TB, swollen lymph node for >14 days, previous history of TB and ≥1 symptom suggestive of TB		
**Exclusion criteria**	N/A		
**Standardized form to guide evaluation?**	YES		
**Standardized form to guide CXR evaluation?**	YES		
**Standardized follow-up for all children?**	YES		
**Reference classification of TB**	Confirmed PTB – bacteriologically positive on culture/Xpert/ on any respiratory specimen (including stool);<br>Unconfirmed PTB – bacteriologically negative or not tested, but clinically diagnosed based on symptoms, CXR, +/-TST, and contact history;<br>Unlikely PTB – not meeting criteria for confirmed TB or unconfirmed TB;<br>Retrospective classification made by managing clinical teams at the 1-month follow-up visit		
**No. screened/No. enrolled**	NA/447		
**Ethics review**	Data collection was approved by the Indus Hospital Research Center IRB.		
**Reference**	[15]		

Table S10. Study information for Zar/2019/ZA

**Geographic setting**	Cape Town, South Africa<br>Gqeberha South Africa		
**Representing co-authors (in no particular order)**	Heather J. Zar, Mark P. Nicol		
**Healthcare setting**	**Name**	**Healthcare level**	**Recruitment setting**
	Red Cross War Memorial Children's Hospital	Tertiary/Referral	Inpatient/Outpatient and Emergency/Casualty
	Dora Nginza Provincial Hospital	Tertiary/Referral	Inpatient/Outpatient and Emergency/Casualty
**Enrolment duration**	01 February 2010 – 31 January 2017		
**Purpose for data collection**	Novel tuberculosis diagnostics in HIV-infected and HIV-uninfected children.		
**Study design**	Prospective cohort study with baseline assessment and follow-up of all children at months 1, 2, and 6.		
**Inclusion criteria**	Children aged 0-15 years with clinical suspicion of PTB based on cough and one of (household TB contact within preceding 3 months, weight loss of failure to gain weight for preceding 3 months, positive TST, or chest radiograph suggestive of PTB) or clinical suspicion of EPTB		
**Exclusion criteria**	Children who had received treatment for tuberculosis or TB prophylaxis for >72 hours prior to enrolment; patients living outside the catchment area; patients for whom adequate clinical samples could not be obtained; or patients for whom informed consent or permission for HIV testing could not be obtained		
**Standardized form to guide evaluation?**	YES		
**Standardized form to guide CXR evaluation?**	YES		
**Standardized follow-up for all children?**	YES		
**Reference classification of TB**	Graham 2015: Confirmed PTB, Unconfirmed PTB, Unlikely PTB;<br>Retrospective classifications made by study team (separate from managing clinical team) using data from all visits up to the 3-month follow-up visit		
**No. screened/No. enrolled**	4548/1346		
**Ethics review**	University of Cape Town Human Research Ethics Committee (HREC), 2008, Ref no. 045/2008.		
**Reference**	[16]		

Table S11. Study information for Walters/2017/ZA

**Geographic setting**	Cape Town, South Africa		
**Representing co-authors (in no particular order)**	Anneke C. Hesseling, Marieke M. van der Zalm, Megan Palmer, Elisabetta G. Walters		
**Healthcare setting**	**Name**	**Healthcare level**	**Recruitment setting**
	Tygerberg Hospital	Tertiary/Referral	Inpatient/Outpatient
	Karl Bremer Hospital	Secondary	Inpatient/Outpatient
**Enrolment duration**	March 2012 – November 2017		
**Purpose for data collection**	Evaluate feasible strategies to improve and promote microbiological testing of children with PTB and characterise treatment response.		
**Study design**	Prospective cohort study with baseline assessment and follow-up of all children at months 1, 2, and 6.		
**Inclusion criteria**	Children 0-12 with any of the following: cough ≥2 weeks, unexplained fever ≥1 week, poor growth/weight loss over the preceding 3 months, or cough <1 week with a known TB exposure in the previous 12 months, a positive TST, or a CXR suggestive of PTB		
**Exclusion criteria**	Children who had received treatment for tuberculosis for >1 day or were being evaluated for EPTB without being evaluated for PTB.		
**Standardized form to guide evaluation?**	YES		
**Standardized form to guide CXR evaluation?**	YES		
**Standardized follow-up for all children?**	YES		
**Reference classification of TB**	Graham 2015: Confirmed PTB, Unconfirmed PTB, Unlikely PTB; Retrospective classifications made by study team (separate from managing clinical team) using information from all visits up to the 2-month follow-up visit		
**No. screened/No. enrolled**	NA/620		
**Ethics review**	Health Research Ethics Committee of Stellenbosch University Faculty of Health Sciences No. N11/09/282		
**Reference**	[17]		

Table S12. Study information for Orikiriza/2018/UG

<table>
<tr><td>**Geographic setting**</td><td colspan="3">Mbarara, Uganda</td></tr>
<tr><td>**Representing co-authors (in no particular order)**</td><td colspan="3">Maryline Bonnet, Patrick Orikiriza</td></tr>
<tr><td rowspan="2">**Healthcare setting**</td><td>**Name**</td><td>**Healthcare level**</td><td>**Recruitment setting**</td></tr>
<tr><td>Mbarara Regional Referral Hospital</td><td>Tertiary/Referral</td><td>Inpatient/Outpatient (includes children referred from TB contact screening)</td></tr>
<tr><td>**Enrolment duration**</td><td colspan="3">12 April 2012 – 14 January 2014</td></tr>
<tr><td>**Purpose for data collection**</td><td colspan="3">Evaluate the performance of Xpert MTB/RIF on induced sputum and to assess treatment outcome and safety of pediatric TB drug dosages.</td></tr>
<tr><td>**Study design**</td><td colspan="3">Prospective cohort study with baseline assessment and follow-up at 3 months for children not started on TB treatment and follow-up at 12 months for children started on TB treatment.</td></tr>
<tr><td>**Inclusion criteria**</td><td colspan="3">Children aged 0-14 with any of: weight loss/failure to thrive/growth faltering over preceding 3 months, non-remittent cough or wheeze >14 days, night sweats in preceding 14 days, unexplained fever for ≥7 days, chest pain within the preceding 2 weeks, unexplained fatigue/weakness/apathy/lethargy in previous 2 weeks, or abnormal CXR suggestive of TB</td></tr>
<tr><td>**Exclusion criteria**</td><td colspan="3">Children who had received >3 days of treatment for tuberculosis or had completed treatment within the past 6 months or with poor access to follow-up evaluation.</td></tr>
<tr><td>**Standardized form to guide evaluation?**</td><td colspan="3">YES</td></tr>
<tr><td>**Standardized form to guide CXR evaluation?**</td><td colspan="3">YES</td></tr>
<tr><td>**Standardized follow-up for all children?**</td><td colspan="3">YES</td></tr>
<tr><td>**No. screened/No. enrolled**</td><td colspan="3">467/392</td></tr>
<tr><td>**Reference classification of TB**</td><td colspan="3">Graham 2012: Confirmed PTB, Probable PTB, Possible PTB, PTB Unlikely; Retrospective classification made by blinded, independent endpoint review committee at 3-month visit for children not started on TB treatment and 6-month visit for children started on TB treatment</td></tr>
<tr><td>**Ethics review**</td><td colspan="3">MUST Research Ethics Committee (MUST-REC), Uganda National Council for Science and Technology (UNCST), Comité de Protection des Personnes (CPP), Iles de France XI France.</td></tr>
<tr><td>**Reference**</td><td colspan="3">[18]</td></tr>
</table>

Table S13. Study information for Orikiriza/2022/UG

**Geographic setting**	Mbarara, Uganda		
**Representing co-authors (in no particular order)**	Maryline Bonnet, Dorah Nampijja		
**Healthcare setting**	**Name**	**Healthcare level**	**Recruitment setting**
	Mbarara Regional Referral Hospital	Tertiary/Referral	Inpatient
**Enrolment duration**	September 2015 – March 2018		
**Purpose for data collection**	Evaluate the performance of Xpert MTB/RIF on stool and urine AlereLAM among children with increased risk of disseminated or severe TB.		
**Study design**	Prospective cohort study with baseline assessment and follow-up at weeks 1, 2, 8, and 24 for all children.		
**Inclusion criteria**	Children aged 0-1 year or HIV-infected or with severe malnutrition and either 1) at least two of the following: cough >2 weeks, fever >1 week, severe malnutrition, >2 lethargy >2 weeks, known exposure to TB within preceding 2 years, or 2) any sign suggestive of TB meningitis or disseminated/miliary TB		
**Exclusion criteria**	Children who received anti-tuberculosis treatment		
**Standardized form to guide evaluation?**	YES		
**Standardized form to guide CXR evaluation?**	YES		
**Standardized follow-up for all children?**	YES		
**Reference classification of TB**	Graham 2015: Confirmed PTB, Unconfirmed PTB, Unlikely PTB;<br>Automated diagnostic algorithm for retrospective classification using information from all visits up to the 6-month follow-up visit, review by independent endpoint committee for cases not classified by the algorithm		
**No. screened/No. enrolled**	238/219		
**Ethics review**	MUST Research Ethics Committee (MUST-REC), Uganda National Council for Science and Technology (UNCST), Comité de Protection des Personnes (CPP), Iles de France XI France.		
**Reference**	[19]		

Table S14. Study information for Giang/2015/VN

**Geographic setting**	Ho Chi Minh City, Vietnam		
**Representing co-authors (in no particular order)**	Julie Huynh, Maxine Caws, Nguyen Thuy Thuong Thuong		
**Healthcare setting**	**Name**	**Healthcare level**	**Recruitment setting**
	Pham Ngoc Thach Hospital	Tertiary/Referral	Inpatient
**Enrolment duration**	01 April 2013 – 01 October 2013		
**Purpose for data collection**	Evaluate the performance of Xpert MTB/RIF for the diagnosis of TB in HIV-uninfected children.		
**Study design**	Prospective cohort study with baseline assessment and unspecified minimum follow-up (consistent with routine clinical practice).		
**Inclusion criteria**	HIV-uninfected children aged 0-15 years with 1 or more of: persistent unexplained fever, cough >2 weeks, night sweats, weight loss, failure to thrive, reduced playfulness/lethargy, and/or any of the following for infants <60 days: neonatal pneumonia, unexplained hepatomegaly, or sepsis-like illness		
**Exclusion criteria**	Children who received anti-tuberculosis treatment prior to specimen collection for Mtb confirmation or children living with HIV.		
**Standardized form to guide evaluation?**	YES		
**Standardized form to guide CXR evaluation?**	NO		
**Standardized follow-up for all children?**	YES		
**Reference classification of TB**	Graham 2012: Confirmed PTB, Probable PTB, Possible PTB, PTB Unlikely; Retrospective classification made by the managing clinical team at 2-month visit		
**No. screened/No. enrolled**	154/150		
**Ethics review**	Pham Ngoc Thach Hospital Institutional review Board (IRB), the Oxford Tropical Ethics Committee (OxTREC) and the Health services of Ho Chi Minh City.		
**Reference**	[20]		

Appendix H: Modifications to IPD received

Table S15. Modifications to IPD from Kabir/2020/BD

VARIABLE	DESCRIPTION	CODE	LABEL	MODIFICATION
**HIV status**	Participant HIV status	0	HIV-negative	HIV status was not collected as a part of this study. In consultation with study authors, we assumed that all children in this study were HIV-negative given low HIV prevalence
		1	HIV-positive	
		NA	Unknown	
**Documented TB exposure**	Known exposure to MTB at initial evaluation in previous 12 months	0	No documented TB exposure in previous 12 months	An exposure was defined as having a family member living with the child who was diagnosed with and received treatment for TB in the previous 12 months.
		1	Documented TB exposure in previous 12 months	
		NA	Unknown	
**First Xpert MTB/RIF**	Result from first Xpert MTB/RIF (not Ultra) performed on ES/IS (or GA for young children) collected at initial evaluation	0	Xpert negative for Mtb	Result from first Xpert performed on induced sputum specimens.
		1	Xpert positive for Mtb	
		NA	Unknown/not performed	
**CXR consistent with TB**	Result of CXR performed at initial evaluation as assessed by reader performing clinical evaluation/making TB-treatment decision or by reader to inform research classification of TB if former not available	0	CXR not consistent with TB	CXR assessment made by the managing clinical team.
		1	CXR consistent with TB	
		NA	Unknown/not assessed	
**TB classification**	Final classification of TB	0	Unlikely TB	See note on reference classification in study description table above.
		1	Bacteriologically-confirmed TB	
		2	Unconfirmed TB	
		NA	Unknown	

Table S16. Modifications to IPD from Aurilio/2020/BR

VARIABLE	DESCRIPTION	CODE	LABEL	MODIFICATION
**Documented TB exposure**	Known exposure to MTB at initial evaluation in previous 12 months	0	No documented TB exposure in previous 12 months	An exposure was defined as a mother, household member, or someone spending ~4 hours a day with the child having documented or reported positive Xpert or TB culture [or receiving treatment for TB] in the previous 12 months).
		1	Documented TB exposure in previous 12 months	
		NA	Unknown	
**First Xpert MTB/RIF**	Result from first Xpert MTB/RIF (not Ultra) performed on ES/IS (or GA for young children) collected at initial evaluation	0	Xpert negative for Mtb	Result from first Xpert: mostly GA/ES/IS, some pleural effusion, bronchoalveolar lavage, and tracheal aspirate.
		1	Xpert positive for Mtb	
		NA	Unknown/not performed	
**CXR consistent with TB**	Result of CXR performed at initial evaluation as assessed by reader performing clinical evaluation/making TB-treatment decision or by reader to inform research classification of TB if former not available	0	CXR not consistent with TB	All CXR assessments made by the study team.
		1	CXR consistent with TB	
		NA	Unknown/not assessed	
**Opacities on CXR**	Opacities (e.g., alveolar consolidation and/or bronchopneumonia) on CXR performed at initial evaluation as assessed by reader performing clinical evaluation/making TB-treatment decision or by reader to inform research classification of TB if former not available	0	Opacities not present on CXR	Received data corresponding to presence of alveolar opacification and bronchopneumonia; if either of these were positive, then the CXR was said to demonstrate opacities.
		1	Opacities present on CXR	
		NA	Unknown/not assessed	
**Intrathoracic lymphadenopathy on CXR**	Intrathoracic lymphadenopathy (e.g., perihilar nodes, paratracheal nodes, mediastinal nodes) on CXR performed at initial evaluation as assessed by reader performing clinical evaluation/making TB-treatment decision or by reader to inform research classification of TB if former not available	0	Intrathoracic lymphadenopathy not present on CXR	Received data corresponding to presence of perihilar lymphadenopathy, paratracheal lymphadenopathy, and calcified nodes; if any of these were positive, then the CXR was said to demonstrate nodes.
		1	Intrathoracic lymphadenopathy present on CXR	
		NA	Unknown/not assessed	
**TB classification**	Final classification of TB	0	Unlikely TB	See note on reference classification in study description table above.
		1	Bacteriologically-confirmed TB	
		2	Unconfirmed TB	
		NA	Unknown	

Table S17. Modifications to IPD from Song/2021/KE

VARIABLE	DESCRIPTION	CODE	LABEL	MODIFICATION
**Documented TB exposure**	Known exposure to MTB at initial evaluation in previous 12 months	0	No documented TB exposure in previous 12 months	Tuberculosis exposure was defined as caregiver-reported household contact with someone with TB within 24 months prior to enrollment.
		1	Documented TB exposure in previous 12 months	
		NA	Unknown	
**First Xpert MTB/RIF**	Result from first Xpert MTB/RIF (not Ultra) performed on ES/IS (or GA for young children) collected at initial evaluation	0	Xpert negative for Mtb	Result from first Xpert: all GA specimens.
		1	Xpert positive for Mtb	
		NA	Unknown/not performed	
**CXR consistent with TB**	Result of CXR performed at initial evaluation as assessed by reader performing clinical evaluation/making TB-treatment decision or by reader to inform research classification of TB if former not available	0	CXR not consistent with TB	CXR assessment made by expert readers
		1	CXR consistent with TB	
		NA	Unknown/not assessed	
**TB classification**	Final classification of TB	0	Unlikely TB	See note on reference classification in study description table above.
		1	Bacteriologically-confirmed TB	
		2	Unconfirmed TB	
		NA	Unknown	

Table S18. Modifications to IPD from LopezVarela/2015/MZ

VARIABLE	DESCRIPTION	CODE	LABEL	MODIFICATION
**Weight loss**	Presenting history of poor growth over the preceding 3 months AND not responding to nutritional rehabilitation (or antiretroviral therapy if HIV infected)	0	No weight loss	Used WAZ/HAZ for this definition.
		1	Weight loss	
		NA	Unknown	
**Documented TB exposure**	Known exposure to MTB at initial evaluation in previous 12 months	0	No documented TB exposure in previous 12 months	Exposure to TB was defined as contact with someone diagnosed as or being treated for TB. No time-limit, but given that all kids were under the age of 3, this would have included any exposure during lifetime. For those children identified through active case finding, the definition was contact with a smear-positive adult with PTB registered at the district National TB Program (NTP) in the previous 24 months.
		1	Documented TB exposure in previous 12 months	
		NA	Unknown	
**Peripheral lymphadenopathy**	Peripheral lymphadenopathy (at cervical, submandibular, and/or axillary nodes) at initial evaluation	0	No peripheral lymphadenopathy	Received data corresponding to presence of cervical lymphadenopathy and axillary lymphadenopathy; if either of these were positive, then the child was said to have peripheral lymphadenopathy. Not all children were assessed for this feature.
		1	Peripheral lymphadenopathy	
		NA	Unknown	
**First Xpert MTB/RIF**	Result from first Xpert MTB/RIF (not Ultra) performed on ES/IS (or GA for young children) collected at initial evaluation	0	Xpert negative for Mtb	Xpert was not performed as a part of this study. We assumed that a positive culture was equivalent to a positive Xpert result. Preferentially used the result from liquid culture or solid culture of the first GA specimen. If GA was not available, then we took the result of either liquid culture or solid culture from the first ES specimen.
		1	Xpert positive for Mtb	
		NA	Unknown/not performed	
**CXR consistent with TB**	Result of CXR performed at initial evaluation as assessed by reader performing clinical evaluation/making TB-treatment decision or by reader to inform research classification of TB if former not available	0	CXR not consistent with TB	All CXR assessments made by the study team, which was the same as the managing clinical team.
		1	CXR consistent with TB	
		NA	Unknown/not assessed	
**Opacities on CXR**	Opacities (e.g., alveolar consolidation and/or bronchopneumonia) on CXR performed at initial evaluation as assessed by reader performing clinical evaluation/making TB-treatment decision or by reader to inform research classification of TB if former not available	0	Opacities not present on CXR	Received data corresponding to presence of alveolar opacification; if this was positive, then the CXR was said to demonstrate opacities.
		1	Opacities present on CXR	
		NA	Unknown/not assessed	
**TB classification**	Final classification of TB	0	Unlikely TB	See note on reference classification in study description table above. 'Probable TB' and 'possible TB' were coded as 'unconfirmed TB,' 'MTB infection' was coded as 'unlikely TB.'
		1	Bacteriologically-confirmed TB	
		2	Unconfirmed TB	
		NA	Unknown	

Table S19. Modifications to IPD from Valencia/2017/MZ

VARIABLE	DESCRIPTION	CODE	LABEL	MODIFICATION
**Documented TB exposure**	Known exposure to MTB at initial evaluation in previous 12 months	0	No documented TB exposure in previous 12 months	An exposure was defined as a mother, household member, or someone spending ~4 hours a day with the child receiving treatment for TB in the previous 12 months.
		1	Documented TB exposure in previous 12 months	
		NA	Unknown	
**Peripheral lymphadenopathy**	Peripheral lymphadenopathy (at cervical, submandibular, and/or axillary nodes) at initial evaluation	0	No peripheral lymphadenopathy	Received data corresponding to presence of cervical lymphadenopathy; if if this was positive, then the child was said to have peripheral lymphadenopathy.
		1	Peripheral lymphadenopathy	
		NA	Unknown	
**First Xpert MTB/RIF**	Result from first Xpert MTB/RIF (not Ultra) performed on ES/IS (or GA for young children) collected at initial evaluation	0	Xpert negative for Mtb	Result from first Xpert: all either ES or IS specimens.
		1	Xpert positive for Mtb	
		NA	Unknown/not performed	
**CXR consistent with TB**	Result of CXR performed at initial evaluation as assessed by reader performing clinical evaluation/making TB-treatment decision or by reader to inform research classification of TB if former not available	0	CXR not consistent with TB	All CXR assessments made by the managing clinical team. CXR was only performed for children for whom the managing clinical team determined that CXR was necessary; thus, not all children had CXR performed.
		1	CXR consistent with TB	
		NA	Unknown/not assessed	
**Opacities on CXR**	Opacities (e.g., alveolar consolidation and/or bronchopneumonia) on CXR performed at initial evaluation as assessed by reader performing clinical evaluation/making TB-treatment decision or by reader to inform research classification of TB if former not available	0	Opacities not present on CXR	Received data corresponding to presence of alveolar opacification and bronchopneumonia; if either of these were positive, then the CXR was said to demonstrate opacities.
		1	Opacities present on CXR	
		NA	Unknown/not assessed	
**Intrathoracic lymphadenopathy on CXR**	Intrathoracic lymphadenopathy (e.g., perihilar nodes, paratracheal nodes, mediastinal nodes) on CXR performed at initial evaluation as assessed by reader performing clinical evaluation/making TB-treatment decision or by reader to inform research classification of TB if former not available	0	Intrathoracic lymphadenopathy not present on CXR	Received data corresponding to presence of perihilar lymphadenopathy, paratracheal lymphadenopathy, and calcified nodes; if any of these were positive, then the CXR was said to demonstrate nodes.
		1	Intrathoracic lymphadenopathy present on CXR	
		NA	Unknown/not assessed	
**TB classification**	Final classification of TB	0	Unlikely TB	See note on reference classification in study description table above.
		1	Bacteriologically-confirmed TB	
		2	Unconfirmed TB	
		NA	Unknown	

Table S20. Modifications to IPD from Myo/2018/MM

VARIABLE	DESCRIPTION	CODE	LABEL	MODIFICATION
**Documented TB exposure**	Known exposure to MTB at initial evaluation in previous 12 months	0	No documented TB exposure in previous 12 months	Defined as a documented or reported exposure to a case of tuberculosis (household or close contact) within the preceding 12 months
		1	Documented TB exposure in previous 12 months	
		NA	Unknown	
**First Xpert MTB/RIF**	Result from first Xpert MTB/RIF (not Ultra) performed on ES/IS (or GA for young children) collected at initial evaluation	0	Xpert negative for Mtb	Result from first Xpert: all GA specimens.
		1	Xpert positive for Mtb	
		NA	Unknown/not performed	
**CXR consistent with TB**	Result of CXR performed at initial evaluation as assessed by reader performing clinical evaluation/making TB-treatment decision or by reader to inform research classification of TB if former not available	0	CXR not consistent with TB	CXR assessment made by the study team.
		1	CXR consistent with TB	
		NA	Unknown/not assessed	
**TB classification**	Final classification of TB	0	Unlikely TB	See note on reference classification in study description table above.
		1	Bacteriologically-confirmed TB	
		2	Unconfirmed TB	
		NA	Unknown	

Table S21. Modifications to IPD from Marcy/2019/Multi

VARIABLE	DESCRIPTION	CODE	LABEL	MODIFICATION
**Fever duration**	Presence of fever 1 week at initial evaluation	0	Fever 1 week not present	Fever duration was not provided in granular enough detail to identify those with fever for greater than or equal to one week.
		1	Fever 1 week present	
		NA	Unknown	
**Lethargy**	Presenting history of unusual lethargy or lack of playfulness at initial evaluation	0	No lethargy	Positive if the patient experienced lethargy in the previous 4 weeks.
		1	Lethargy	
		NA	Unknown	
**Weight loss**	Presenting history of poor growth over the preceding 3 months AND not responding to nutritional rehabilitation (or antiretroviral therapy if HIV infected)	0	No weight loss	Positive if the patient experienced weight loss in the previous 4 weeks.
		1	Weight loss	
		NA	Unknown	
**Documented TB exposure**	Known exposure to MTB at initial evaluation in previous 12 months	0	No documented TB exposure in previous 12 months	Exposure defined as having a household contact with smear + TB in the previous 12 months.
		1	Documented TB exposure in previous 12 months	
		NA	Unknown	
**Night sweats**	Presenting history of night sweats at initial evaluation	0	No night sweats	Positive if the patient experienced night sweats in the previous 4 weeks.
		1	Night sweats	
		NA	Unknown	
**Haemoptysis**	Presenting history of haemoptysis at initial evaluation	0	No haemoptysis	Positive if the patient experienced haemoptysis in the previous 4 weeks.
		1	Haemoptysis	
		NA	Unknown	
**Peripheral lymphadenopathy**	Peripheral lymphadenopathy (at cervical, submandibular, and/or axillary nodes) at initial evaluation	0	No peripheral lymphadenopathy	Received data corresponding to presence of cervical lymphadenopathy, submandibular lymphadenopathy, and axillary lymphadenopathy; if any of these were positive, then the child was said to have peripheral lymphadenopathy.
		1	Peripheral lymphadenopathy	
		NA	Unknown	
**First Xpert MTB/RIF**	Result from first Xpert MTB/RIF (not Ultra) performed on ES/IS (or GA for young children) collected at initial evaluation	0	Xpert negative for Mtb	Result from first Xpert: mostly ES, with some IS and GA specimens.
		1	Xpert positive for Mtb	
		NA	Unknown/not performed	
**CXR consistent with TB**	Result of CXR performed at initial evaluation as assessed by reader performing clinical evaluation/making TB-treatment decision or by reader to inform research classification of TB if former not available	0	CXR not consistent with TB	All CXR assessments made by the managing clinical team.
		1	CXR consistent with TB	
		NA	Unknown/not assessed	
**Opacities on CXR**	Opacities (e.g., alveolar consolidation and/or bronchopneumonia) on CXR performed at initial evaluation as assessed by reader performing clinical evaluation/making TB-treatment decision or by reader to inform research classification of TB if former not available	0	Opacities not present on CXR	Received data corresponding to presence of alveolar opacification; if this was positive, then the CXR was said to demonstrate opacities.
		1	Opacities present on CXR	
		NA	Unknown/not assessed	
**Intrathoracic lymphadenopathy on CXR**	Intrathoracic lymphadenopathy (e.g., perihilar nodes, paratracheal nodes, mediastinal nodes) on CXR performed at initial evaluation as assessed by reader performing clinical evaluation/making TB-treatment decision or by reader to inform research classification of TB if former not available	0	Intrathoracic lymphadenopathy not present on CXR	Received data corresponding to presence of perihilar lymphadenopathy and paratracheal lymphadenopathy; if either of these were positive, then the CXR was said to demonstrate nodes.
		1	Intrathoracic lymphadenopathy present on CXR	
		NA	Unknown/not assessed	
**TB classification**	Final classification of TB	0	Unlikely TB	See note on reference classification in study description table above.
		1	Bacteriologically-confirmed TB	
		2	Unconfirmed TB	
		NA	Unknown	

Table S22. Modifications to IPD from Hamid/2019/PK

VARIABLE	DESCRIPTION	CODE	LABEL	MODIFICATION
**Age (months)**	Age (months) at enrolment	###	NA = unknown	Age was reported as years old; assumed to be at midpoint of year and converted to months.
**HIV status**	Participant HIV status	0	HIV-negative	HIV status was not collected as a part of this study. In consultation with study authors, we assumed that all children in this study were HIV-negative.
		1	HIV-positive	
		NA	Unknown	
**Weight loss**	Presenting history of poor growth over the preceding 3 months AND not responding to nutritional rehabilitation (or antiretroviral therapy if HIV infected)	0	No weight loss	Defined as subjective weight loss reported by parents/guardians.
		1	Weight loss	
		NA	Unknown	
**Documented TB exposure**	Known exposure to MTB at initial evaluation in previous 12 months	0	No documented TB exposure in previous 12 months	An exposure was defined as a mother, household member, or someone spending ~4 hours a day with the child having documented or reported positive Xpert or TB culture (or receiving treatment for TB) in the previous 24 months.
		1	Documented TB exposure in previous 12 months	
		NA	Unknown	
**First Xpert MTB/RIF**	Result from first Xpert MTB/RIF (not Ultra) performed on ES/IS (or GA for young children) collected at initial evaluation	0	Xpert negative for Mtb	Result from first Xpert: performed only on stool specimens. Not all children received Xpert testing.
		1	Xpert positive for Mtb	
		NA	Unknown/not performed	
**CXR consistent with TB**	Result of CXR performed at initial evaluation as assessed by reader performing clinical evaluation/making TB-treatment decision or by reader to inform research classification of TB if former not available	0	CXR not consistent with TB	All CXR assessments made by the managing clinical team.
		1	CXR consistent with TB	
		NA	Unknown/not assessed	
**TB classification**	Final classification of TB	0	Unlikely TB	Eighteen children were given a diagnosis of EPTB; these children were removed from the analysis. Otherwise, see note on reference classification in study description table above.
		1	Bacteriologically-confirmed TB	
		2	Unconfirmed TB	
		NA	Unknown	

Table S23. Modifications to IPD from Zar/2019/ZA

VARIABLE	DESCRIPTION	CODE	LABEL	MODIFICATION
**Documented TB exposure**	Known exposure to MTB at initial evaluation in previous 12 months	0	No documented TB exposure in previous 12 months	An exposure was defined as a mother, household member, or someone spending ~4 hours a day with the child having documented or reported positive Xpert or TB culture (or receiving treatment for TB) in the previous 24 months.
		1	Documented TB exposure in previous 12 months	
		NA	Unknown	
**First Xpert MTB/RIF**	Result from first Xpert MTB/RIF (not Ultra) performed on ES/IS (or GA for young children) collected at initial evaluation	0	Xpert negative for Mtb	Result from first Xpert: performed only on IS specimens.
		1	Xpert positive for Mtb	
		NA	Unknown/not performed	
**CXR consistent with TB**	Result of CXR performed at initial evaluation as assessed by reader performing clinical evaluation/making TB-treatment decision or by reader to inform research classification of TB if former not available	0	CXR not consistent with TB	All CXR assessments made by the study team; many were determined to be inconclusive for PTB.
		1	CXR consistent with TB	
		NA	Unknown/not assessed	
**Opacities on CXR**	Opacities (e.g., alveolar consolidation and/or bronchopneumonia) on CXR performed at initial evaluation as assessed by reader performing clinical evaluation/making TB-treatment decision or by reader to inform research classification of TB if former not available	0	Opacities not present on CXR	Received data corresponding to presence of alveolar opacification; if positive, then the CXR was said to demonstrate opacities.
		1	Opacities present on CXR	
		NA	Unknown/not assessed	
**Intrathoracic lymphadenopath y on CXR**	Intrathoracic lymphadenopathy (e.g., perihilar nodes, paratracheal nodes, mediastinal nodes) on CXR performed at initial evaluation as assessed by reader performing clinical evaluation/making TB-treatment decision or by reader to inform research classification of TB if former not available	0	Intrathoracic lymphadenopathy not present on CXR	Received data corresponding to presence of perihilar lymphadenopathy and paratracheal lymphadenopathy; if either of these were positive, then the CXR was said to demonstrate nodes.
		1	Intrathoracic lymphadenopathy present on CXR	
		NA	Unknown/not assessed	
**TB classification**	Final classification of TB	0	Unlikely TB	Removed data from 37 individuals with EPTB as not relevant to the analysis population. Otherwise, see note on reference classification in study description table above.
		1	Bacteriologically-confirmed TB	
		2	Unconfirmed TB	
		NA	Unknown	

Table S24. Modifications to IPD from Walters/2017/ZA

VARIABLE	DESCRIPTION	CODE	LABEL	MODIFICATION
**Weight loss**	Presenting history of poor growth over the preceding 3 months AND not responding to nutritional rehabilitation (or antiretroviral therapy if HIV infected)	0	No weight loss	Weight loss was specifically defined as follows: Poor growth documented over the preceding 3 months (clear deviation from the child's previous growth trajectory and/or static growth or weight loss in the preceding 3 months; alternatively, weight-for-age Z-score (WFAZ) ≤2 in children with no previous weight measurements).
		1	Weight loss	
		NA	Unknown	
**Documented TB exposure**	Known exposure to MTB at initial evaluation in previous 12 months	0	No documented TB exposure in previous 12 months	Exposure to any identified adult TB source case in the preceding 12 months, where exposure was either within the household; or involved the child's primary caregiver; or occurred for >4 hours per day during the period of exposure.
		1	Documented TB exposure in previous 12 months	
		NA	Unknown	
**Peripheral lymphadenopathy**	Peripheral lymphadenopathy (at cervical, submandibular, and/or axillary nodes) at initial evaluation	0	No peripheral lymphadenopathy	Received data corresponding to presence of cervical lymphadenopathy, submandibular lymphadenopathy, and axillary lymphadenopathy; if any of these were positive, then the child was said to have peripheral lymphadenopathy.
		1	Peripheral lymphadenopathy	
		NA	Unknown	
**First Xpert MTB/RIF**	Result from first Xpert MTB/RIF (not Ultra) performed on ES/IS (or GA for young children) collected at initial evaluation	0	Xpert negative for Mtb	Result from first Xpert performed.
		1	Xpert positive for Mtb	
		NA	Unknown/not performed	
**CXR consistent with TB**	Result of CXR performed at initial evaluation as assessed by reader performing clinical evaluation/making TB-treatment decision or by reader to inform research classification of TB if former not available	0	CXR not consistent with TB	All CXR assessments made by the study team.
		1	CXR consistent with TB	
		NA	Unknown/not assessed	
**Opacities on CXR**	Opacities (e.g., alveolar consolidation and/or bronchopneumonia) on CXR performed at initial evaluation as assessed by reader performing clinical evaluation/making TB-treatment decision or by reader to inform research classification of TB if former not available	0	Opacities not present on CXR	Received data corresponding to presence of alveolar opacification and bronchopneumonia; if either of these were positive, then the CXR was said to demonstrate opacities.
		1	Opacities present on CXR	
		NA	Unknown/not assessed	
**Intrathoracic lymphadenopathy on CXR**	Intrathoracic lymphadenopathy (e.g., perihilar nodes, paratracheal nodes, mediastinal nodes) on CXR performed at initial evaluation as assessed by reader performing clinical evaluation/making TB-treatment decision or by reader to inform research classification of TB if former not available	0	Intrathoracic lymphadenopathy not present on CXR	Received data corresponding to presence of perihilar lymphadenopathy, paratracheal lymphadenopathy, and calcified nodes; if any of these were positive, then the CXR was said to demonstrate nodes.
		1	Intrathoracic lymphadenopathy present on CXR	
		NA	Unknown/not assessed	
**TB classification**	Final classification of TB	0	Unlikely TB	See note on reference classification in study description table above.
		1	Bacteriologically-confirmed TB	
		2	Unconfirmed TB	
		NA	Unknown	

Table S25. Modifications to IPD from Orikiriza/2018/UG

VARIABLE	DESCRIPTION	CODE	LABEL	MODIFICATION
**Cough duration**	Duration of cough at initial evaluation	0	No cough	Duration of cough was only provided a greater than or equal to 2 weeks or less than 2 weeks/no cough.
		1	Cough 0-13 days	
		2	Cough 14-20 days	
		3	Cough 21-27 days	
		4	Cough 28 days	
		NA	Unknown	
**Fever duration**	Duration of fever at initial evaluation	0	No fever	Duration of fever was only provided as greater than or equal to 1 week or less than 1 week/no cough.
		1	Fever 0-13 days	
		2	Fever 14-20 days	
		3	Fever 21-27 days	
		4	Fever 28 days	
		NA	Unknown	
**Lethargy**	Presenting history of unusual lethargy or lack of playfulness at initial evaluation	0	No lethargy	Lethargy was positive if present for greater than or equal to 2 weeks; negative if no lethargy or for less than 2 weeks.
		1	Lethargy	
		NA	Unknown	
**Documented TB exposure**	Known exposure to MTB at initial evaluation in previous 12 months	0	No documented TB exposure in previous 12 months	For children referred from another contact study (any child who has lived in the same household with the index case continuously for at least 2 weeks within the 3-month period immediately preceding the diagnosis of smear-positive or culture-positive TB in the index case. For other children, documented as reported contact with a bacteriologically-positive case within the preceding 12 months.
		1	Documented TB exposure in previous 12 months	
		NA	Unknown	
**Night sweats**	Presenting history of night sweats at initial evaluation	0	No night sweats	Night sweats coded using the following scale: absent, mild, moderate, severe, or life threatening. Recoded absent = 0, and others = 1.
		1	Night sweats	
		NA	Unknown	
**Peripheral lymphadenopathy**	Peripheral lymphadenopathy (at cervical, submandibular, and/or axillary nodes) at initial evaluation	0	No peripheral lymphadenopathy	Location of peripheral lymphadenopathy not specified.
		1	Peripheral lymphadenopathy	
		NA	Unknown	
**First Xpert MTB/RIF**	Result from first Xpert MTB/RIF (not Ultra) performed on ES/IS (or GA for young children) collected at initial evaluation	0	Xpert negative for Mtb	Result from first Xpert: performed on two pooled IS specimens.
		1	Xpert positive for Mtb	
		NA	Unknown/not performed	
**CXR consistent with TB**	Result of CXR performed at initial evaluation as assessed by reader performing clinical evaluation/making TB-treatment decision or by reader to inform research classification of TB if former not available	0	CXR not consistent with TB	All CXR assessments made by the managing clinical team.
		1	CXR consistent with TB	
		NA	Unknown/not assessed	
**Opacities on CXR**	Opacities (e.g., alveolar consolidation and/or bronchopneumonia) on CXR performed at initial evaluation as assessed by reader performing clinical evaluation/making TB-treatment decision or by reader to inform research classification of TB if former not available	0	Opacities not present on CXR	Received data corresponding to presence of alveolar opacification and bronchopneumonia; if either of these were positive, then the CXR was said to demonstrate opacities.
		1	Opacities present on CXR	
		NA	Unknown/not assessed	
**Intrathoracic lymphadenopathy on CXR**	Intrathoracic lymphadenopathy (e.g., perihilar nodes, paratracheal nodes, mediastinal nodes) on CXR performed at initial evaluation as assessed by reader performing clinical evaluation/making TB-treatment decision or by reader to inform research classification of TB if former not available	0	Intrathoracic lymphadenopathy not present on CXR	Positive if mediastinal lymphadenopathy was present.
		1	Intrathoracic lymphadenopathy present on CXR	
		NA	Unknown/not assessed	
**TB classification**	Final classification of TB	0	Unlikely TB	See note on reference classification in study description table above. 'Probable TB' and 'possible TB' were coded as 'unconfirmed TB.'
		1	Bacteriologically-confirmed TB	
		2	Unconfirmed TB	
		NA	Unknown	

Table S26. Modifications to IPD from Orikiriza/2022/UG

VARIABLE	DESCRIPTION	CODE	LABEL	MODIFICATION
**BCG vaccination**	Evidence of BCG vaccination (BCG scar or BCG recorded in immunization record) at initial evaluation	0	No evidence of BCG vaccination	If had a BCG-scar or a positive immunization card/verbal response, then determined to have evidence of BCG vaccination.
		1	Evidence of BCG vaccination	
		NA	Unknown	
**Cough duration**	Duration of cough at initial evaluation	0	No cough	Duration of cough was only provided a greater than or equal to 2 weeks or less than 2 weeks/no cough.
		1	Cough 0-13 days	
		2	Cough 14-20 days	
		3	Cough 21-27 days	
		4	Cough 28 days	
		NA	Unknown	
**Fever duration**	Duration of fever at initial evaluation	0	No fever	Duration of fever was only provided as greater than or equal to 1 week or less than 1 week/no cough.
		1	Fever 0-13 days	
		2	Fever 14-20 days	
		3	Fever 21-27 days	
		4	Fever 28 days	
		NA	Unknown	
**Documented TB exposure**	Known exposure to MTB at initial evaluation in previous 12 months	0	No documented TB exposure in previous 12 months	Contact of a household member with positive Xpert or TB culture in the previous 12 months
		1	Documented TB exposure in previous 12 months	
		NA	Unknown	
**Peripheral lymphadenopathy**	Peripheral lymphadenopathy (at cervical, submandibular, and/or axillary nodes) at initial evaluation	0	No peripheral lymphadenopathy	Significant peripheral lymphadenopathy on screening (location unspecified).
		1	Peripheral lymphadenopathy	
		NA	Unknown	
**First Xpert MTB/RIF**	Result from first Xpert MTB/RIF (not Ultra) performed on ES/IS (or GA for young children) collected at initial evaluation	0	Xpert negative for Mtb	Result from first Xpert: performed on GA specimens.
		1	Xpert positive for Mtb	
		NA	Unknown/not performed	
**CXR consistent with TB**	Result of CXR performed at initial evaluation as assessed by reader performing clinical evaluation/making TB-treatment decision or by reader to inform research classification of TB if former not available	0	CXR not consistent with TB	All CXR assessments made by the managing clinical team.
		1	CXR consistent with TB	
		NA	Unknown/not assessed	
**Opacities on CXR**	Opacities (e.g., alveolar consolidation and/or bronchopneumonia) on CXR performed at initial evaluation as assessed by reader performing clinical evaluation/making TB-treatment decision or by reader to inform research classification of TB if former not available	0	Opacities not present on CXR	Received data corresponding to presence of alveolar opacification and bronchopneumonia; if either of these were positive, then the CXR was said to demonstrate opacities.
		1	Opacities present on CXR	
		NA	Unknown/not assessed	
**Intrathoracic lymphadenopathy on CXR**	Intrathoracic lymphadenopathy (e.g., perihilar nodes, paratracheal nodes, mediastinal nodes) on CXR performed at initial evaluation as assessed by reader performing clinical evaluation/making TB-treatment decision or by reader to inform research classification of TB if former not available	0	Intrathoracic lymphadenopathy not present on CXR	Received data corresponding to presence of Gohn focus and hilar lymphadenopathy (grouped together) and mediastinal nodes; if either of these were positive, then the CXR was said to demonstrate nodes.
		1	Intrathoracic lymphadenopathy present on CXR	
		NA	Unknown/not assessed	
**TB classification**	Final classification of TB	0	Unlikely TB	See note on reference classification in study description table above.
		1	Bacteriologically-confirmed TB	
		2	Unconfirmed TB	
		NA	Unknown	

Table S27. Modifications to IPD from Giang/2015/VN

VARIABLE	DESCRIPTION	CODE	LABEL	MODIFICATION
**Cough duration**	Duration of cough at initial evaluation	0	No cough	Duration of cough was only provided a greater than or equal to 2 weeks or less than 2 weeks/no cough.
		1	Cough 0-13 days	
		2	Cough 14-20 days	
		3	Cough 21-27 days	
		4	Cough 28 days	
		NA	Unknown	
**Fever duration**	Duration of fever at initial evaluation	0	No fever	Duration of fever was only provided as greater than or equal to 1 week or less than 1 week/no cough.
		1	Fever 0-13 days	
		2	Fever 14-20 days	
		3	Fever 21-27 days	
		4	Fever 28 days	
		NA	Unknown	
**Weight loss**	Presenting history of poor growth over the preceding 3 months AND not responding to nutritional rehabilitation (or antiretroviral therapy if HIV infected)	0	No weight loss	Subjective weight loss and/or failure to thrive.
		1	Weight loss	
		NA	Unknown	
**Documented TB exposure**	Known exposure to MTB at initial evaluation in previous 12 months	0	No documented TB exposure in previous 12 months	Exposure was defined as a household or close contact with a TB case (unspecified).
		1	Documented TB exposure in previous 12 months	
		NA	Unknown	
**Peripheral lymphadenopathy**	Peripheral lymphadenopathy (at cervical, submandibular, and/or axillary nodes) at initial evaluation	0	No peripheral lymphadenopathy	Received data corresponding to presence of cervical lymphadenopathy and submandibular lymphadenopathy; if either of these were positive, then the child was said to have peripheral lymphadenopathy.
		1	Peripheral lymphadenopathy	
		NA	Unknown	
**First Xpert MTB/RIF**	Result from first Xpert MTB/RIF (not Ultra) performed on ES/IS (or GA for young children) collected at initial evaluation	0	Xpert negative for Mtb	Result from first Xpert: performed on mostly GA specimens.
		1	Xpert positive for Mtb	
		NA	Unknown/not performed	
**CXR consistent with TB**	Result of CXR performed at initial evaluation as assessed by reader performing clinical evaluation/making TB-treatment decision or by reader to inform research classification of TB if former not available	0	CXR not consistent with TB	Unclear whether this data corresponds to result as assessed by the study team or the managing clinical team.
		1	CXR consistent with TB	
		NA	Unknown/not assessed	
**TB classification**	Final classification of TB	0	Unlikely TB	See note on reference classification in study description table above. 'Probable TB' and 'possible TB' were coded as 'unconfirmed TB.' One child unable to classify as 'probable TB' or 'possible TB' in original data was coded as 'unconfirmed TB' for the purposes of this analysis
		1	Bacteriologically-confirmed TB	
		2	Unconfirmed TB	
		NA	Unknown	

Appendix I: Missingness in IPD received

Figure S2. Missingness in IPD received (note variables names per Table S1)

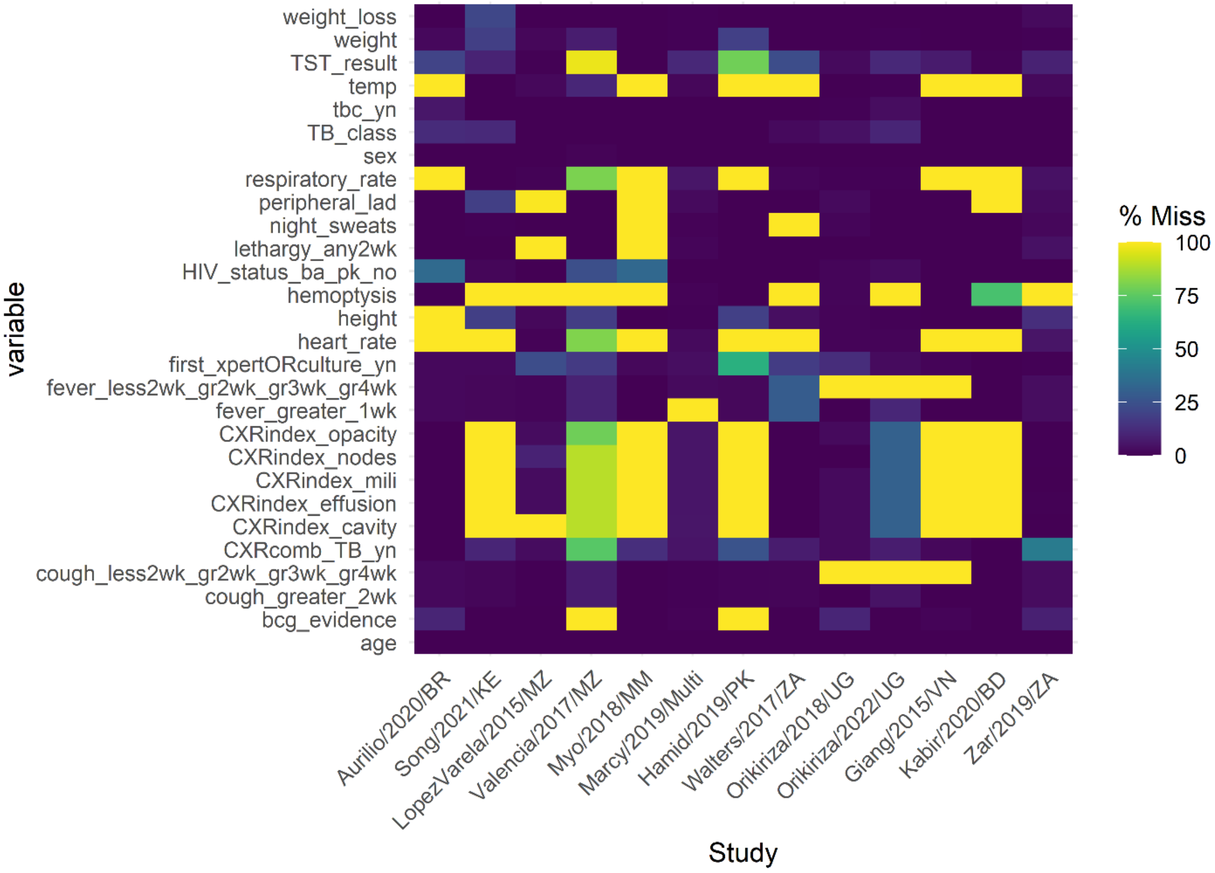

Appendix J: Generate additional variables

After imputation, additional variables were computed from requested variables as follows:

- **Temperature >38°C**
    - Objective temperature recorded as greater than 38°C
- **Tachycardia**
    - Children <2 months old, heart rate >160
    - Children 2-12 months old, heart rate >150
    - Children 12 months – 5 years old, heart rate >140
    - Children >5 years old, heart rate >120
- **Tachypnoea**
    - Children <2 months old, respiratory rate >60
    - Children 2-12 months old, respiratory rate >50
    - Children 12 months – 5 years old, respiratory rate >40
    - Children >5 years old, respiratory rate >30
- **Weight-for-age Z-score**
    - Determined from sex, age, and weight as per WHO Child Growth Standards
    - Implemented in function “addWGSR” in R package *zscorer*
- **Weight-for-height Z-score**
    - Determined from sex, weight, and height as per WHO Child Growth Standards
    - Implemented in function “addWGSR” in R package *zscorer*
- **Body-mass-index-for-height Z-score**
    - Determined from sex, weight, height, and age as per WHO Child Growth Standards
    - Implemented in function “addWGSR” in R package *zscorer*
- **Severely acutely malnourished**
    - Children <5 years old, weight-for-height Z-score <-3
    - Children ≥5 years old, body-mass-index-for-height Z-score <-3

Appendix K: Descriptions of study-level IPD

All clinical, bacteriology, and imaging data collected from the initial visit. IPD – Individual participant data, TB – tuberculosis, IQR – interquartile range, BCG – bacille Calmette-Guerin, HIV – human immunodeficiency virus, Mtb – *Mycobacterium tuberculosis*, CXR – chest x-ray, TST – tuberculin skin test.

Table S28. Description of IPD from Kabir/2020/BD

Variable	Value	Confirmed TB (N=63)	Unconfirmed TB (N=36)	Unlikely TB (N=303)
		n (%) OR median (IQR)	n (%) OR median (IQR)	n (%) OR median (IQR)
Age	Months	25·6 (11,69·6)	41·5 (7·4,83·6)	18·5 (8,57·3)
	Unknown	0 (0)	0 (0)	0 (0)
Sex	Male	36 (57)	24 (67)	177 (58)
	Female	27 (43)	12 (33)	126 (42)
	Unknown	0 (0)	0 (0)	0 (0)
Weight-for-age	z-score	-2.6 (-4.1,-1.5)	-2.7 (-3.7,-1.5)	-2.5 (-3.6,-1.3)
	Unknown	0 (0)	0 (0)	0 (0)
BCG vaccination	Evidence of BCG vaccination	54 (86)	32 (89)	227 (75)
	No evidence of BCG vaccination	9 (14)	4 (11)	76 (25)
	Unknown	0 (0)	0 (0)	0 (0)
HIV status	HIV-positive	0 (0)	0 (0)	0 (0)
	HIV-negative	63 (100)	36 (100)	303 (100)
	Unknown	0 (0)	0 (0)	0 (0)
Cough duration	No cough	2 (3)	1 (3)	10 (3)
	Cough 0-13 days	18 (29)	7 (19)	71 (23)
	Cough 14-20 days	15 (24)	5 (14)	81 (27)
	Cough 21-27 days	4 (6)	7 (19)	31 (10)
	Cough >27 days	24 (38)	16 (44)	110 (36)
	Unknown	0 (0)	0 (0)	0 (0)
Cough ≥ 2 weeks	Cough ≥2 weeks present	43 (68)	28 (78)	222 (73)
	Cough ≥2 weeks not present	20 (32)	8 (22)	81 (27)
	Unknown	0 (0)	0 (0)	0 (0)
Fever duration	No fever	1 (2)	2 (6)	13 (4)
	Fever 0-13 days	14 (22)	7 (19)	94 (31)
	Fever 14-20 days	16 (25)	10 (28)	77 (25)
	Fever 21-27 days	7 (11)	3 (8)	26 (9)
	Fever >27 days	25 (40)	14 (39)	93 (31)
	Unknown	0 (0)	0 (0)	0 (0)
Fever ≥1 week	Fever ≥1 week present	58 (92)	32 (89)	255 (84)
	Fever ≥1 week not present	5 (8)	4 (11)	48 (16)
	Unknown	0 (0)	0 (0)	0 (0)
Lethargy	Lethargy	60 (95)	33 (92)	282 (93)
	No lethargy	3 (5)	3 (8)	21 (7)
	Unknown	0 (0)	0 (0)	0 (0)
Weight loss	Weight loss	54 (86)	33 (92)	255 (84)
	No weight loss	9 (14)	3 (8)	48 (16)
	Unknown	0 (0)	0 (0)	0 (0)
Documented TB exposure	Documented TB exposure in previous 12 months	17 (27)	17 (47)	65 (21)
	No documented TB exposure in previous 12 months	46 (73)	19 (53)	238 (79)
	Unknown	0 (0)	0 (0)	0 (0)
Night sweats	Night sweats	42 (67)	25 (69)	179 (59)
	No night sweats	21 (33)	11 (31)	124 (41)
	Unknown	0 (0)	0 (0)	0 (0)
Haemoptysis	Haemoptysis	0 (0)	1 (3)	7 (2)
	No haemoptysis	9 (14)	6 (17)	94 (31)
	Unknown	54 (86)	29 (81)	202 (67)
Objective fever (≥38 degrees Celsius)	Objective fever	0 (0)	0 (0)	0 (0)
	No objective fever	0 (0)	0 (0)	0 (0)
	Unknown	63 (100)	36 (100)	303 (100)
Tachycardia	Tachycardia	0 (0)	0 (0)	0 (0)
	No tachycardia	0 (0)	0 (0)	0 (0)
	Unknown	63 (100)	36 (100)	303 (100)
Tachypnoea	Tachypnoea	0 (0)	0 (0)	0 (0)
	No tachypnoea	0 (0)	0 (0)	0 (0)
	Unknown	63 (100)	36 (100)	303 (100)
Peripheral lymphadenopathy	Peripheral lymphadenopathy	0 (0)	0 (0)	0 (0)
	No peripheral lymphadenopathy	0 (0)	0 (0)	0 (0)
	Unknown	63 (100)	36 (100)	303 (100)

Variable	Value	Confirmed TB (N=63)	Unconfirmed TB (N=36)	Unlikely TB (N=303)
		n (%) OR median (IQR)	n (%) OR median (IQR)	n (%) OR median (IQR)
First Xpert MTB/RIF	Xpert positive for Mtb	9 (14)	0 (0)	0 (0)
	Xpert negative for Mtb	54 (86)	36 (100)	303 (100)
	Unknown/not performed	0 (0)	0 (0)	0 (0)
Overall CXR read	CXR consistent with TB	57 (90)	31 (86)	274 (90)
	CXR not consistent with TB	6 (10)	5 (14)	29 (10)
	Unknown/not assessed	0 (0)	0 (0)	0 (0)
Opacities on CXR	Opacities present on CXR	0 (0)	0 (0)	0 (0)
	Opacities not present on CXR	0 (0)	0 (0)	0 (0)
	Unknown/not assessed	63 (100)	36 (100)	303 (100)
Cavities on CXR	Cavities present on CXR	0 (0)	0 (0)	0 (0)
	Cavities not present on CXR	0 (0)	0 (0)	0 (0)
	Unknown/not assessed	63 (100)	36 (100)	303 (100)
Miliary infiltrate on CXR	Miliary infiltrate present on CXR	0 (0)	0 (0)	0 (0)
	Miliary infiltrate not present on CXR	0 (0)	0 (0)	0 (0)
	Unknown/not assessed	63 (100)	36 (100)	303 (100)
Intrathoracic lymphadenopathy on CXR	Intrathoracic lymphadenopathy present on CXR	0 (0)	0 (0)	0 (0)
	Intrathoracic lymphadenopathy not present on CXR	0 (0)	0 (0)	0 (0)
	Unknown/not assessed	63 (100)	36 (100)	303 (100)
Pleural effusion on CXR	Pleural effusion present on CXR	0 (0)	0 (0)	0 (0)
	Pleural effusion not present on CXR	0 (0)	0 (0)	0 (0)
	Unknown/not assessed	63 (100)	36 (100)	303 (100)
Tuberculin skin test	TST positive	24 (38)	25 (69)	36 (12)
	TST negative	39 (62)	11 (31)	265 (87)
	Unknown/not performed	0 (0)	0 (0)	2 (1)

Table S29. Description of IPD from Aurilio/2020/BR

Variable	Value	Confirmed TB (N=9)	Unconfirmed TB (N=11)	Unlikely TB (N=24)	TB-status unknown (N=6)
		n (%) OR median (IQR)	n (%) OR median (IQR)	n (%) OR median (IQR)	n (%) OR median (IQR)
Age	Months	31 (9,65)	58 (17·5,89)	32·5 (12,72·2)	33 (18,79·5)
	Unknown	0 (0)	0 (0)	0 (0)	0 (0)
Sex	Male	2 (22)	6 (55)	10 (42)	1 (17)
	Female	7 (78)	5 (45)	14 (58)	5 (83)
	Unknown	0 (0)	0 (0)	0 (0)	0 (0)
Weight-for-age	z-score	0.3 (-1.7,0.6)	-0.3 (-1.7,-0.1)	0.1 (-1.1,0.7)	-1.2 (-1.3,-0.6)
	Unknown	0 (0)	0 (0)	0 (0)	1 (17)
BCG vaccination	Evidence of BCG vaccination	9 (100)	9 (82)	23 (96)	4 (67)
	No evidence of BCG vaccination	0 (0)	0 (0)	0 (0)	0 (0)
	Unknown	0 (0)	2 (18)	1 (4)	2 (33)
HIV status	HIV-positive	0 (0)	1 (9)	4 (17)	1 (17)
	HIV-negative	8 (89)	9 (82)	6 (25)	4 (67)
	Unknown	1 (11)	1 (9)	14 (58)	1 (17)
Cough duration	No cough	4 (44)	4 (36)	12 (50)	2 (33)
	Cough 0-13 days	1 (11)	2 (18)	4 (17)	2 (33)
	Cough 14-20 days	2 (22)	2 (18)	3 (12)	1 (17)
	Cough 21-27 days	0 (0)	0 (0)	2 (8)	0 (0)
	Cough >27 days	1 (11)	3 (27)	3 (12)	1 (17)
	Unknown	1 (11)	0 (0)	0 (0)	0 (0)
Cough ≥2 weeks	Cough ≥2 weeks present	3 (33)	5 (45)	8 (33)	2 (33)
	Cough ≥2 weeks not present	5 (56)	6 (55)	16 (67)	4 (67)
	Unknown	1 (11)	0 (0)	0 (0)	0 (0)
Fever duration	No fever	3 (33)	1 (9)	5 (21)	1 (17)
	Fever 0-13 days	2 (22)	4 (36)	8 (33)	2 (33)
	Fever 14-20 days	1 (11)	3 (27)	4 (17)	2 (33)
	Fever 21-27 days	0 (0)	0 (0)	1 (4)	0 (0)
	Fever >27 days	2 (22)	3 (27)	6 (25)	1 (17)
	Unknown	1 (11)	0 (0)	0 (0)	0 (0)
Fever ≥1 week	Fever ≥1 week present	3 (33)	8 (73)	14 (58)	3 (50)
	Fever ≥1 week not present	5 (56)	3 (27)	10 (42)	3 (50)
	Unknown	1 (11)	0 (0)	0 (0)	0 (0)
Lethargy	Lethargy	0 (0)	1 (9)	2 (8)	4 (67)
	No lethargy	9 (100)	10 (91)	22 (92)	2 (33)
	Unknown	0 (0)	0 (0)	0 (0)	0 (0)
Weight loss	Weight loss	3 (33)	4 (36)	5 (21)	4 (67)
	No weight loss	6 (67)	7 (64)	19 (79)	2 (33)
	Unknown	0 (0)	0 (0)	0 (0)	0 (0)
Documented TB exposure	Documented TB exposure in previous 12 months	6 (67)	6 (55)	11 (46)	2 (33)
	No documented TB exposure in previous 12 months	3 (33)	5 (45)	11 (46)	3 (50)
	Unknown	0 (0)	0 (0)	2 (8)	1 (17)
Night sweats	Night sweats	0 (0)	1 (9)	0 (0)	0 (0)
	No night sweats	9 (100)	10 (91)	24 (100)	6 (100)
	Unknown	0 (0)	0 (0)	0 (0)	0 (0)
Haemoptysis	Haemoptysis	0 (0)	0 (0)	0 (0)	0 (0)
	No haemoptysis	9 (100)	11 (100)	24 (100)	6 (100)
	Unknown	0 (0)	0 (0)	0 (0)	0 (0)
Objective fever (≥38 degrees Celsius)	Objective fever	0 (0)	0 (0)	0 (0)	0 (0)
	No objective fever	0 (0)	0 (0)	0 (0)	0 (0)
	Unknown	9 (100)	11 (100)	24 (100)	6 (100)
Tachycardia	Tachycardia	0 (0)	0 (0)	0 (0)	0 (0)
	No tachycardia	0 (0)	0 (0)	0 (0)	0 (0)
	Unknown	9 (100)	11 (100)	24 (100)	6 (100)
Tachypnoea	Tachypnoea	0 (0)	0 (0)	0 (0)	0 (0)
	No tachypnoea	0 (0)	0 (0)	0 (0)	0 (0)
	Unknown	9 (100)	11 (100)	24 (100)	6 (100)
Peripheral lymphadenopathy	Peripheral lymphadenopathy	0 (0)	0 (0)	0 (0)	0 (0)
	No peripheral lymphadenopathy	9 (100)	11 (100)	24 (100)	6 (100)
	Unknown	0 (0)	0 (0)	0 (0)	0 (0)

Variable	Value	Confirmed TB (N=9)	Unconfirmed TB (N=11)	Unlikely TB (N=24)	TB-status unknown (N=6)
First Xpert MTB/RIF	Xpert positive for Mtb	7 (78)	0 (0)	0 (0)	0 (0)
	Xpert negative for Mtb	2 (22)	11 (100)	23 (96)	6 (100)
	Unknown/not performed	0 (0)	0 (0)	1 (4)	0 (0)
Overall CXR read	CXR consistent with TB	4 (44)	8 (73)	1 (4)	1 (17)
	CXR not consistent with TB	5 (56)	3 (27)	23 (96)	5 (83)
	Unknown/not assessed	0 (0)	0 (0)	0 (0)	0 (0)
Opacities on CXR	Opacities present on CXR	6 (67)	4 (36)	15 (62)	3 (50)
	Opacities not present on CXR	3 (33)	7 (64)	9 (38)	3 (50)
	Unknown/not assessed	0 (0)	0 (0)	0 (0)	0 (0)
Cavities on CXR	Cavities present on CXR	0 (0)	0 (0)	1 (4)	1 (17)
	Cavities not present on CXR	9 (100)	11 (100)	23 (96)	5 (83)
	Unknown/not assessed	0 (0)	0 (0)	0 (0)	0 (0)
Miliary infiltrate on CXR	Miliary infiltrate present on CXR	1 (11)	3 (27)	0 (0)	0 (0)
	Miliary infiltrate not present on CXR	8 (89)	8 (73)	24 (100)	6 (100)
	Unknown/not assessed	0 (0)	0 (0)	0 (0)	0 (0)
Intrathoracic lymphadenopathy on CXR	Intrathoracic lymphadenopathy present on CXR	1 (11)	2 (18)	1 (4)	0 (0)
	Intrathoracic lymphadenopathy not present on CXR	8 (89)	9 (82)	23 (96)	6 (100)
	Unknown/not assessed	0 (0)	0 (0)	0 (0)	0 (0)
Pleural effusion on CXR	Pleural effusion present on CXR	3 (33)	3 (27)	8 (33)	2 (33)
	Pleural effusion not present on CXR	6 (67)	8 (73)	16 (67)	4 (67)
	Unknown/not assessed	0 (0)	0 (0)	0 (0)	0 (0)
Tuberculin skin test	TST positive	4 (44)	4 (36)	4 (17)	0 (0)
	TST negative	2 (22)	6 (55)	18 (75)	2 (33)
	Unknown/not performed	3 (33)	1 (9)	2 (8)	4 (67)

Table S30. Description of IPD from Song/2021/KE

Variable	Value	Confirmed TB (N=31)	Unconfirmed TB (N=65)	Unlikely TB (N=170)	TB-status unknown (N=34)
		n (%) OR median (IQR)	n (%) OR median (IQR)	n (%) OR median (IQR)	n (%) OR median (IQR)
Age	Months	23·2 (12·3,38·1)	24·3 (10·5,39·5)	28·1 (15·5,46·7)	17·6 (9·4,34)
	Unknown	0 (0)	0 (0)	0 (0)	0 (0)
Sex	Male	14 (45)	31 (48)	86 (51)	18 (53)
	Female	17 (55)	34 (52)	84 (49)	16 (47)
	Unknown	0 (0)	0 (0)	0 (0)	0 (0)
Weight-for-age	z-score	-0.5 (-1.8,0.2)	-1.1 (-2,-0.2)	-0.6 (-1.6,0.4)	-1.7 (-2,0.2)
	Unknown	6 (19)	7 (11)	18 (11)	24 (71)
BCG vaccination	Evidence of BCG vaccination	27 (87)	63 (97)	162 (95)	33 (97)
	No evidence of BCG vaccination	4 (13)	2 (3)	8 (5)	1 (3)
	Unknown	0 (0)	0 (0)	0 (0)	0 (0)
HIV status	HIV-positive	7 (23)	24 (37)	33 (19)	9 (26)
	HIV-negative	22 (71)	40 (62)	136 (80)	25 (74)
	Unknown	2 (6)	1 (2)	1 (1)	0 (0)
Cough duration	No cough	5 (16)	2 (3)	16 (9)	2 (6)
	Cough 0-13 days	1 (3)	7 (11)	17 (10)	4 (12)
	Cough 14-20 days	2 (6)	7 (11)	10 (6)	3 (9)
	Cough 21-27 days	3 (10)	1 (2)	4 (2)	0 (0)
	Cough >27 days	20 (65)	48 (74)	120 (71)	24 (71)
	Unknown	0 (0)	0 (0)	3 (2)	1 (3)
Cough ≥2 weeks	Cough ≥2 weeks present	25 (81)	56 (86)	134 (79)	27 (79)
	Cough ≥2 weeks not present	6 (19)	9 (14)	33 (19)	6 (18)
	Unknown	0 (0)	0 (0)	3 (2)	1 (3)
Fever duration	No fever	11 (35)	26 (40)	74 (44)	14 (41)
	Fever 0-13 days	7 (23)	17 (26)	49 (29)	7 (21)
	Fever 14-20 days	7 (23)	16 (25)	26 (15)	5 (15)
	Fever 21-27 days	4 (13)	2 (3)	6 (4)	2 (6)
	Fever >27 days	1 (3)	4 (6)	12 (7)	5 (15)
	Unknown	1 (3)	0 (0)	3 (2)	1 (3)
Fever ≥1 week	Fever ≥1 week present	16 (52)	31 (48)	75 (44)	16 (47)
	Fever ≥1 week not present	14 (45)	34 (52)	92 (54)	17 (50)
	Unknown	1 (3)	0 (0)	3 (2)	1 (3)
Lethargy	Lethargy	13 (42)	20 (31)	45 (26)	14 (41)
	No lethargy	18 (58)	45 (69)	125 (74)	20 (59)
	Unknown	0 (0)	0 (0)	0 (0)	0 (0)
Weight loss	Weight loss	0 (0)	3 (5)	3 (2)	0 (0)
	No weight loss	23 (74)	54 (83)	144 (85)	9 (26)
	Unknown	8 (26)	8 (12)	23 (14)	25 (74)
Documented TB exposure	Documented TB exposure in previous 12 months	24 (77)	31 (48)	30 (18)	4 (12)
	No documented TB exposure in previous 12 months	7 (23)	34 (52)	140 (82)	30 (88)
	Unknown	0 (0)	0 (0)	0 (0)	0 (0)
Night sweats	Night sweats	22 (71)	37 (57)	83 (49)	13 (38)
	No night sweats	9 (29)	27 (42)	87 (51)	21 (62)
	Unknown	0 (0)	1 (2)	0 (0)	0 (0)
Haemoptysis	Haemoptysis	0 (0)	0 (0)	0 (0)	0 (0)
	No haemoptysis	0 (0)	0 (0)	0 (0)	0 (0)
	Unknown	31 (100)	65 (100)	170 (100)	34 (100)
Objective fever (≥38 degrees Celsius)	Objective fever	4 (13)	6 (9)	7 (4)	5 (15)
	No objective fever	27 (87)	59 (91)	163 (96)	29 (85)
	Unknown	0 (0)	0 (0)	0 (0)	0 (0)
Tachycardia	Tachycardia	0 (0)	0 (0)	0 (0)	0 (0)
	No tachycardia	0 (0)	0 (0)	0 (0)	0 (0)
	Unknown	31 (100)	65 (100)	170 (100)	34 (100)
Tachypnoea	Tachypnoea	3 (10)	3 (5)	3 (2)	3 (9)
	No tachypnoea	28 (90)	62 (95)	167 (98)	31 (91)
	Unknown	0 (0)	0 (0)	0 (0)	0 (0)
Peripheral lymphadenopathy	Peripheral lymphadenopathy	0 (0)	0 (0)	1 (1)	0 (0)
	No peripheral lymphadenopathy	25 (81)	58 (89)	151 (89)	10 (29)
	Unknown	6 (19)	7 (11)	18 (11)	24 (71)

Variable	Value	Confirmed TB (N=31)	Unconfirmed TB (N=65)	Unlikely TB (N=170)	TB-status unknown (N=34)
		n (%) OR median (IQR)	n (%) OR median (IQR)	n (%) OR median (IQR)	n (%) OR median (IQR)
First Xpert MTB/RIF	Xpert positive for Mtb	14 (45)	0 (0)	0 (0)	0 (0)
	Xpert negative for Mtb	15 (48)	65 (100)	168 (99)	32 (94)
	Unknown/not performed	2 (6)	0 (0)	2 (1)	2 (6)
Overall CXR read	CXR consistent with TB	13 (42)	16 (25)	14 (8)	2 (6)
	CXR not consistent with TB	17 (55)	43 (66)	150 (88)	15 (44)
	Unknown/not assessed	1 (3)	6 (9)	6 (4)	17 (50)
Opacities on CXR	Opacities present on CXR	0 (0)	0 (0)	0 (0)	0 (0)
	Opacities not present on CXR	0 (0)	0 (0)	0 (0)	0 (0)
	Unknown/not assessed	31 (100)	65 (100)	170 (100)	34 (100)
Cavities on CXR	Cavities present on CXR	0 (0)	0 (0)	0 (0)	0 (0)
	Cavities not present on CXR	0 (0)	0 (0)	0 (0)	0 (0)
	Unknown/not assessed	31 (100)	65 (100)	170 (100)	34 (100)
Miliary infiltrate on CXR	Miliary infiltrate present on CXR	0 (0)	0 (0)	0 (0)	0 (0)
	Miliary infiltrate not present on CXR	0 (0)	0 (0)	0 (0)	0 (0)
	Unknown/not assessed	31 (100)	65 (100)	170 (100)	34 (100)
Intrathoracic lymphadenopathy on CXR	Intrathoracic lymphadenopathy present on CXR	0 (0)	0 (0)	0 (0)	0 (0)
	Intrathoracic lymphadenopathy not present on CXR	0 (0)	0 (0)	0 (0)	0 (0)
	Unknown/not assessed	31 (100)	65 (100)	170 (100)	34 (100)
Pleural effusion on CXR	Pleural effusion present on CXR	0 (0)	0 (0)	0 (0)	0 (0)
	Pleural effusion not present on CXR	0 (0)	0 (0)	0 (0)	0 (0)
	Unknown/not assessed	31 (100)	65 (100)	170 (100)	34 (100)
Tuberculin skin test	TST positive	15 (48)	15 (23)	17 (10)	1 (3)
	TST negative	11 (35)	44 (68)	146 (86)	23 (68)
	Unknown/not performed	5 (16)	6 (9)	7 (4)	10 (29)

Table S31. Description of IPD from LopezVarela/2015/MZ

Variable	Value	Confirmed TB (N=13)	Unconfirmed TB (N=128)	Unlikely TB (N=648)
		n (%) OR median (IQR)	n (%) OR median (IQR)	n (%) OR median (IQR)
Age	Months	20·3 (10·6,24·1)	19·5 (12·9,26)	19·8 (14,25·7)
	Unknown	0 (0)	0 (0)	0 (0)
Sex	Male	5 (38)	72 (56)	353 (54)
	Female	8 (62)	56 (44)	295 (46)
	Unknown	0 (0)	0 (0)	0 (0)
Weight-for-age	z-score	-2.3 (-3.8,-1.5)	-2.4 (-3.5,-1.4)	-1.9 (-2.9,-1.1)
	Unknown	0 (0)	2 (2)	10 (2)
BCG vaccination	Evidence of BCG vaccination	13 (100)	127 (99)	648 (100)
	No evidence of BCG vaccination	0 (0)	1 (1)	0 (0)
	Unknown	0 (0)	0 (0)	0 (0)
HIV status	HIV-positive	2 (15)	42 (33)	60 (9)
	HIV-negative	11 (85)	86 (67)	588 (91)
	Unknown	0 (0)	0 (0)	0 (0)
Cough duration	No cough	8 (62)	86 (67)	479 (74)
	Cough 0-13 days	0 (0)	24 (19)	70 (11)
	Cough 14-20 days	2 (15)	5 (4)	34 (5)
	Cough 21-27 days	1 (8)	6 (5)	29 (4)
	Cough >27 days	2 (15)	7 (5)	35 (5)
	Unknown	0 (0)	0 (0)	1 (0)
Cough ≥2 weeks	Cough ≥2 weeks present	5 (38)	18 (14)	98 (15)
	Cough ≥2 weeks not present	8 (62)	110 (86)	549 (85)
	Unknown	0 (0)	0 (0)	1 (0)
Fever duration	No fever	9 (69)	103 (80)	569 (88)
	Fever 0-13 days	2 (15)	22 (17)	59 (9)
	Fever 14-20 days	1 (8)	1 (1)	10 (2)
	Fever 21-27 days	0 (0)	0 (0)	2 (0)
	Fever >27 days	0 (0)	0 (0)	1 (0)
	Unknown	1 (8)	2 (2)	7 (1)
Fever ≥1 week	Fever ≥1 week present	1 (8)	4 (3)	16 (2)
	Fever ≥1 week not present	11 (85)	122 (95)	625 (96)
	Unknown	1 (8)	2 (2)	7 (1)
Lethargy	Lethargy	0 (0)	0 (0)	0 (0)
	No lethargy	0 (0)	0 (0)	0 (0)
	Unknown	13 (100)	128 (100)	648 (100)
Weight loss	Weight loss	8 (62)	106 (83)	554 (85)
	No weight loss	5 (38)	22 (17)	94 (15)
	Unknown	0 (0)	0 (0)	0 (0)
Documented TB exposure	Documented TB exposure in previous 12 months	2 (15)	26 (20)	59 (9)
	No documented TB exposure in previous 12 months	11 (85)	102 (80)	589 (91)
	Unknown	0 (0)	0 (0)	0 (0)
Night sweats	Night sweats	1 (8)	0 (0)	0 (0)
	No night sweats	12 (92)	128 (100)	648 (100)
	Unknown	0 (0)	0 (0)	0 (0)
Haemoptysis	Haemoptysis	0 (0)	0 (0)	0 (0)
	No haemoptysis	0 (0)	0 (0)	0 (0)
	Unknown	13 (100)	128 (100)	648 (100)
Objective fever (≥38 degrees Celsius)	Objective fever	1 (8)	5 (4)	21 (3)
	No objective fever	11 (85)	119 (93)	618 (95)
	Unknown	1 (8)	4 (3)	9 (1)
Tachycardia	Tachycardia	1 (8)	3 (2)	9 (1)
	No tachycardia	11 (85)	123 (96)	637 (98)
	Unknown	1 (8)	2 (2)	2 (0)
Tachypnoea	Tachypnoea	2 (15)	10 (8)	24 (4)
	No tachypnoea	10 (77)	116 (91)	623 (96)
	Unknown	1 (8)	2 (2)	1 (0)
Peripheral lymphadenopathy	Peripheral lymphadenopathy	1 (8)	2 (2)	1 (0)
	No peripheral lymphadenopathy	0 (0)	0 (0)	0 (0)
	Unknown	12 (92)	126 (98)	647 (100)

Variable	Value	Confirmed TB (N=13)	Unconfirmed TB (N=128)	Unlikely TB (N=648)
		n (%) OR median (IQR)	n (%) OR median (IQR)	n (%) OR median (IQR)
First Xpert MTB/RIF	Xpert positive for Mtb	6 (46)	0 (0)	0 (0)
	Xpert negative for Mtb	4 (31)	99 (77)	498 (77)
	Unknown/not performed	3 (23)	29 (23)	150 (23)
Overall CXR read	CXR consistent with TB	8 (62)	62 (48)	90 (14)
	CXR not consistent with TB	3 (23)	66 (52)	537 (83)
	Unknown/not assessed	2 (15)	0 (0)	21 (3)
Opacities on CXR	Opacities present on CXR	8 (62)	58 (45)	89 (14)
	Opacities not present on CXR	3 (23)	70 (55)	538 (83)
	Unknown/not assessed	2 (15)	0 (0)	21 (3)
Cavities on CXR	Cavities present on CXR	0 (0)	0 (0)	0 (0)
	Cavities not present on CXR	0 (0)	0 (0)	0 (0)
	Unknown/not assessed	13 (100)	128 (100)	648 (100)
Miliary infiltrate on CXR	Miliary infiltrate present on CXR	0 (0)	1 (1)	0 (0)
	Miliary infiltrate not present on CXR	11 (85)	127 (99)	627 (97)
	Unknown/not assessed	2 (15)	0 (0)	21 (3)
Intrathoracic lymphadenopathy on CXR	Intrathoracic lymphadenopathy present on CXR	1 (8)	8 (6)	6 (1)
	Intrathoracic lymphadenopathy not present on CXR	10 (77)	102 (80)	589 (91)
	Unknown/not assessed	2 (15)	18 (14)	53 (8)
Pleural effusion on CXR	Pleural effusion present on CXR	0 (0)	5 (4)	3 (0)
	Pleural effusion not present on CXR	11 (85)	123 (96)	624 (96)
	Unknown/not assessed	2 (15)	0 (0)	21 (3)
Tuberculin skin test	TST positive	3 (23)	26 (20)	45 (7)
	TST negative	10 (77)	100 (78)	603 (93)
	Unknown/not performed	0 (0)	2 (2)	0 (0)

Table S32. Description of IPD from Valencia/2017/MZ

Variable	Value	Confirmed TB (N=5)	Unconfirmed TB (N=28)	Unlikely TB (N=109)
		n (%) OR median (IQR)	n (%) OR median (IQR)	n (%) OR median (IQR)
Age	Months	13·7 (12·1,40·1)	22·2 (12·3,54·3)	38·9 (13·8,83·8)
	Unknown	0 (0)	0 (0)	0 (0)
Sex	Male	4 (80)	16 (57)	61 (56)
	Female	1 (20)	11 (39)	48 (44)
	Unknown	0 (0)	1 (4)	0 (0)
Weight-for-age	z-score	-4 (-4.5,-1.4)	-2.3 (-3.8,-1.2)	-2.1 (-3.5,-0.9)
	Unknown	0 (0)	5 (18)	7 (6)
BCG vaccination	Evidence of BCG vaccination	0 (0)	0 (0)	0 (0)
	No evidence of BCG vaccination	0 (0)	0 (0)	0 (0)
	Unknown	5 (100)	28 (100)	109 (100)
HIV status	HIV-positive	3 (60)	17 (61)	50 (46)
	HIV-negative	1 (20)	9 (32)	28 (26)
	Unknown	1 (20)	2 (7)	31 (28)
Cough duration	No cough	0 (0)	3 (11)	11 (10)
	Cough 0-13 days	2 (40)	7 (25)	40 (37)
	Cough 14-20 days	1 (20)	5 (18)	21 (19)
	Cough 21-27 days	0 (0)	1 (4)	3 (3)
	Cough >27 days	2 (40)	10 (36)	26 (24)
	Unknown	0 (0)	2 (7)	8 (7)
Cough ≥2 weeks	Cough ≥2 weeks present	3 (60)	16 (57)	50 (46)
	Cough ≥2 weeks not present	2 (40)	10 (36)	51 (47)
	Unknown	0 (0)	2 (7)	8 (7)
Fever duration	No fever	1 (20)	6 (21)	41 (38)
	Fever 0-13 days	2 (40)	10 (36)	46 (42)
	Fever 14-20 days	0 (0)	3 (11)	5 (5)
	Fever 21-27 days	0 (0)	1 (4)	1 (1)
	Fever >27 days	2 (40)	6 (21)	5 (5)
	Unknown	0 (0)	2 (7)	11 (10)
Fever ≥1 week	Fever ≥1 week present	4 (80)	15 (54)	32 (29)
	Fever ≥1 week not present	1 (20)	11 (39)	66 (61)
	Unknown	0 (0)	2 (7)	11 (10)
Lethargy	Lethargy	4 (80)	7 (25)	45 (41)
	No lethargy	1 (20)	21 (75)	64 (59)
	Unknown	0 (0)	0 (0)	0 (0)
Weight loss	Weight loss	5 (100)	21 (75)	66 (61)
	No weight loss	0 (0)	7 (25)	43 (39)
	Unknown	0 (0)	0 (0)	0 (0)
Documented TB exposure	Documented TB exposure in previous 12 months	2 (40)	9 (32)	21 (19)
	No documented TB exposure in previous 12 months	3 (60)	19 (68)	88 (81)
	Unknown	0 (0)	0 (0)	0 (0)
Night sweats	Night sweats	1 (20)	9 (32)	33 (30)
	No night sweats	4 (80)	19 (68)	76 (70)
	Unknown	0 (0)	0 (0)	0 (0)
Haemoptysis	Haemoptysis	0 (0)	0 (0)	0 (0)
	No haemoptysis	0 (0)	0 (0)	0 (0)
	Unknown	5 (100)	28 (100)	109 (100)
Objective fever (≥38 degrees Celsius)	Objective fever	0 (0)	5 (18)	16 (15)
	No objective fever	5 (100)	19 (68)	82 (75)
	Unknown	0 (0)	4 (14)	11 (10)
Tachycardia	Tachycardia	0 (0)	0 (0)	0 (0)
	No tachycardia	3 (60)	24 (86)	0 (0)
	Unknown	2 (40)	4 (14)	109 (100)
Tachypnoea	Tachypnoea	0 (0)	3 (11)	0 (0)
	No tachypnoea	3 (60)	22 (79)	0 (0)
	Unknown	2 (40)	3 (11)	109 (100)
Peripheral lymphadenopathy	Peripheral lymphadenopathy	0 (0)	8 (29)	20 (18)
	No peripheral lymphadenopathy	5 (100)	20 (71)	89 (82)
	Unknown	0 (0)	0 (0)	0 (0)

Variable	Value	Confirmed TB (N=5)	Unconfirmed TB (N=28)	Unlikely TB (N=109)
		n (%) OR median (IQR)	n (%) OR median (IQR)	n (%) OR median (IQR)
First Xpert MTB/RIF	Xpert positive for Mtb	5 (100)	0 (0)	0 (0)
	Xpert negative for Mtb	0 (0)	22 (79)	91 (83)
	Unknown/not performed	0 (0)	6 (21)	18 (17)
Overall CXR read	CXR consistent with TB	1 (20)	0 (0)	0 (0)
	CXR not consistent with TB	1 (20)	7 (25)	28 (26)
	Unknown/not assessed	3 (60)	21 (75)	81 (74)
Opacities on CXR	Opacities present on CXR	2 (40)	5 (18)	17 (16)
	Opacities not present on CXR	0 (0)	7 (25)	0 (0)
	Unknown/not assessed	3 (60)	16 (57)	92 (84)
Cavities on CXR	Cavities present on CXR	0 (0)	3 (11)	0 (0)
	Cavities not present on CXR	2 (40)	9 (32)	1 (1)
	Unknown/not assessed	3 (60)	16 (57)	108 (99)
Miliary infiltrate on CXR	Miliary infiltrate present on CXR	0 (0)	0 (0)	0 (0)
	Miliary infiltrate not present on CXR	2 (40)	12 (43)	1 (1)
	Unknown/not assessed	3 (60)	16 (57)	108 (99)
Intrathoracic lymphadenopathy on CXR	Intrathoracic lymphadenopathy present on CXR	1 (20)	7 (25)	0 (0)
	Intrathoracic lymphadenopathy not present on CXR	1 (20)	5 (18)	1 (1)
	Unknown/not assessed	3 (60)	16 (57)	108 (99)
Pleural effusion on CXR	Pleural effusion present on CXR	0 (0)	2 (7)	0 (0)
	Pleural effusion not present on CXR	2 (40)	10 (36)	1 (1)
	Unknown/not assessed	3 (60)	16 (57)	108 (99)
Tuberculin skin test	TST positive	0 (0)	0 (0)	0 (0)
	TST negative	1 (20)	3 (11)	0 (0)
	Unknown/not performed	4 (80)	25 (89)	109 (100)

Table S33. Description of IPD from Myo/2018/MM

Variable	Value	Confirmed TB (N=27)	Unconfirmed TB (N=84)	Unlikely TB (N=112)
		n (%) OR median (IQR)	n (%) OR median (IQR)	n (%) OR median (IQR)
Age	Months	22 (7,60)	36 (12,72)	36 (17·2,72)
	Unknown	0 (0)	0 (0)	0 (0)
Sex	Male	9 (33)	47 (56)	65 (58)
	Female	18 (67)	37 (44)	47 (42)
	Unknown	0 (0)	0 (0)	0 (0)
Weight-for-age	z-score	-2.2 (-3.4,-1)	-1.7 (-2.9,-0.9)	-1.8 (-3,-1)
	Unknown	0 (0)	0 (0)	0 (0)
BCG vaccination	Evidence of BCG vaccination	13 (48)	61 (73)	82 (73)
	No evidence of BCG vaccination	14 (52)	23 (27)	30 (27)
	Unknown	0 (0)	0 (0)	0 (0)
HIV status	HIV-positive	4 (15)	8 (10)	15 (13)
	HIV-negative	23 (85)	49 (58)	50 (45)
	Unknown	0 (0)	27 (32)	47 (42)
Cough duration	No cough	0 (0)	0 (0)	0 (0)
	Cough 0-13 days	9 (33)	45 (54)	62 (55)
	Cough 14-20 days	0 (0)	6 (7)	10 (9)
	Cough 21-27 days	18 (67)	33 (39)	40 (36)
	Cough >27 days	0 (0)	0 (0)	0 (0)
	Unknown	0 (0)	0 (0)	0 (0)
Cough ≥2 weeks	Cough ≥2 weeks present	18 (67)	39 (46)	50 (45)
	Cough ≥2 weeks not present	9 (33)	45 (54)	62 (55)
	Unknown	0 (0)	0 (0)	0 (0)
Fever duration	No fever	0 (0)	3 (4)	3 (3)
	Fever 0-13 days	5 (19)	35 (42)	46 (41)
	Fever 14-20 days	6 (22)	19 (23)	34 (30)
	Fever 21-27 days	1 (4)	4 (5)	6 (5)
	Fever >27 days	15 (56)	23 (27)	23 (21)
	Unknown	0 (0)	0 (0)	0 (0)
Fever ≥1 week	Fever ≥1 week present	25 (93)	73 (87)	91 (81)
	Fever ≥1 week not present	2 (7)	11 (13)	21 (19)
	Unknown	0 (0)	0 (0)	0 (0)
Lethargy	Lethargy	0 (0)	0 (0)	0 (0)
	No lethargy	0 (0)	0 (0)	0 (0)
	Unknown	27 (100)	84 (100)	112 (100)
Weight loss	Weight loss	24 (89)	59 (70)	91 (81)
	No weight loss	3 (11)	25 (30)	21 (19)
	Unknown	0 (0)	0 (0)	0 (0)
Documented TB exposure	Documented TB exposure in previous 12 months	11 (41)	40 (48)	37 (33)
	No documented TB exposure in previous 12 months	16 (59)	44 (52)	75 (67)
	Unknown	0 (0)	0 (0)	0 (0)
Night sweats	Night sweats	0 (0)	0 (0)	0 (0)
	No night sweats	0 (0)	0 (0)	0 (0)
	Unknown	27 (100)	84 (100)	112 (100)
Hemoptysis	Haemoptysis	0 (0)	0 (0)	0 (0)
	No haemoptysis	0 (0)	0 (0)	0 (0)
	Unknown	27 (100)	84 (100)	112 (100)
Objective fever (≥38 degrees Celsius)	Objective fever	0 (0)	0 (0)	0 (0)
	No objective fever	0 (0)	0 (0)	0 (0)
	Unknown	27 (100)	84 (100)	112 (100)
Tachycardia	Tachycardia	0 (0)	0 (0)	0 (0)
	No tachycardia	0 (0)	0 (0)	0 (0)
	Unknown	27 (100)	84 (100)	112 (100)
Tachypnoea	Tachypnoea	0 (0)	0 (0)	0 (0)
	No tachypnoea	0 (0)	0 (0)	0 (0)
	Unknown	27 (100)	84 (100)	112 (100)
Peripheral lymphadenopathy	Peripheral lymphadenopathy	0 (0)	0 (0)	0 (0)
	No peripheral lymphadenopathy	0 (0)	0 (0)	0 (0)
	Unknown	27 (100)	84 (100)	112 (100)

Variable	Value	Confirmed TB (N=27)	Unconfirmed TB (N=84)	Unlikely TB (N=112)
		n (%) OR median (IQR)	n (%) OR median (IQR)	n (%) OR median (IQR)
First Xpert MTB/RIF	Xpert positive for Mtb	26 (96)	0 (0)	0 (0)
	Xpert negative for Mtb	1 (4)	84 (100)	108 (96)
	Unknown/not performed	0 (0)	0 (0)	4 (4)
Overall CXR read	CXR consistent with TB	26 (96)	61 (73)	44 (39)
	CXR not consistent with TB	0 (0)	20 (24)	43 (38)
	Unknown/not assessed	1 (4)	3 (4)	25 (22)
Opacities on CXR	Opacities present on CXR	0 (0)	0 (0)	0 (0)
	Opacities not present on CXR	0 (0)	0 (0)	0 (0)
	Unknown/not assessed	27 (100)	84 (100)	112 (100)
Cavities on CXR	Cavities present on CXR	0 (0)	0 (0)	0 (0)
	Cavities not present on CXR	0 (0)	0 (0)	0 (0)
	Unknown/not assessed	27 (100)	84 (100)	112 (100)
Miliary infiltrate on CXR	Miliary infiltrate present on CXR	0 (0)	0 (0)	0 (0)
	Miliary infiltrate not present on CXR	0 (0)	0 (0)	0 (0)
	Unknown/not assessed	27 (100)	84 (100)	112 (100)
Intrathoracic lymphadenopathy on CXR	Intrathoracic lymphadenopathy present on CXR	0 (0)	0 (0)	0 (0)
	Intrathoracic lymphadenopathy not present on CXR	0 (0)	0 (0)	0 (0)
	Unknown/not assessed	27 (100)	84 (100)	112 (100)
Pleural effusion on CXR	Pleural effusion present on CXR	0 (0)	0 (0)	0 (0)
	Pleural effusion not present on CXR	0 (0)	0 (0)	0 (0)
	Unknown/not assessed	27 (100)	84 (100)	112 (100)
Tuberculin skin test	TST positive	15 (56)	18 (21)	4 (4)
	TST negative	12 (44)	66 (79)	108 (96)
	Unknown/not performed	0 (0)	0 (0)	0 (0)

Table S34. Description of IPD from Marcy/2019/Multi

Variable	Value	Confirmed TB (N=41)	Unconfirmed TB (N=155)	Unlikely TB (N=142)
		n (%) OR median (IQR)	n (%) OR median (IQR)	n (%) OR median (IQR)
Age	Months	85·6 (37·7,97·7)	70·8 (35·5,94·3)	60·5 (19·4,98·6)
	Unknown	0 (0)	0 (0)	0 (0)
Sex	Male	22 (54)	85 (55)	61 (43)
	Female	19 (46)	70 (45)	81 (57)
	Unknown	0 (0)	0 (0)	0 (0)
Weight-for-age	z-score	-2.5 (-3.2,-1.3)	-2.8 (-3.9,-2)	-2.5 (-3.6,-1.3)
	Unknown	1 (2)	0 (0)	0 (0)
BCG vaccination	Evidence of BCG vaccination	37 (90)	114 (74)	119 (84)
	No evidence of BCG vaccination	3 (7)	41 (26)	22 (15)
	Unknown	1 (2)	0 (0)	1 (1)
HIV status	HIV-positive	41 (100)	155 (100)	142 (100)
	HIV-negative	0 (0)	0 (0)	0 (0)
	Unknown	0 (0)	0 (0)	0 (0)
Cough duration	No cough	5 (12)	7 (5)	9 (6)
	Cough 0-13 days	3 (7)	12 (8)	20 (14)
	Cough 14-20 days	7 (17)	38 (25)	34 (24)
	Cough 21-27 days	2 (5)	17 (11)	14 (10)
	Cough >27 days	23 (56)	81 (52)	65 (46)
	Unknown	1 (2)	0 (0)	0 (0)
Cough ≥2 weeks	Cough ≥2 weeks present	32 (78)	136 (88)	113 (80)
	Cough ≥2 weeks not present	8 (20)	19 (12)	29 (20)
	Unknown	1 (2)	0 (0)	0 (0)
Fever duration	No fever	4 (10)	23 (15)	46 (32)
	Fever 0-13 days	8 (20)	25 (16)	47 (33)
	Fever 14-20 days	11 (27)	38 (25)	19 (13)
	Fever 21-27 days	1 (2)	16 (10)	8 (6)
	Fever >27 days	14 (34)	50 (32)	20 (14)
	Unknown	3 (7)	3 (2)	2 (1)
Fever ≥1 week	Fever ≥1 week present	0 (0)	0 (0)	0 (0)
	Fever ≥1 week not present	0 (0)	0 (0)	0 (0)
	Unknown	41 (100)	155 (100)	142 (100)
Lethargy	Lethargy	23 (56)	77 (50)	63 (44)
	No lethargy	16 (39)	76 (49)	79 (56)
	Unknown	2 (5)	2 (1)	0 (0)
Weight loss	Weight loss	35 (85)	104 (67)	83 (58)
	No weight loss	5 (12)	51 (33)	59 (42)
	Unknown	1 (2)	0 (0)	0 (0)
Documented TB exposure	Documented TB exposure in previous 12 months	8 (20)	14 (9)	9 (6)
	No documented TB exposure in previous 12 months	33 (80)	141 (91)	133 (94)
	Unknown	0 (0)	0 (0)	0 (0)
Night sweats	Night sweats	10 (24)	71 (46)	42 (30)
	No night sweats	30 (73)	83 (54)	100 (70)
	Unknown	1 (2)	1 (1)	0 (0)
Hemoptysis	Haemoptysis	1 (2)	10 (6)	1 (1)
	No haemoptysis	39 (95)	145 (94)	140 (99)
	Unknown	1 (2)	0 (0)	1 (1)
Objective fever (≥38 degrees Celsius)	Objective fever	20 (49)	22 (14)	25 (18)
	No objective fever	20 (49)	133 (86)	113 (80)
	Unknown	1 (2)	0 (0)	4 (3)
Tachycardia	Tachycardia	15 (37)	21 (14)	12 (8)
	No tachycardia	25 (61)	133 (86)	124 (87)
	Unknown	1 (2)	1 (1)	6 (4)
Tachypnoea	Tachypnoea	14 (34)	47 (30)	40 (28)
	No tachypnoea	22 (54)	106 (68)	90 (63)
	Unknown	5 (12)	2 (1)	12 (8)
Peripheral lymphadenopathy	Peripheral lymphadenopathy	14 (34)	53 (34)	48 (34)
	No peripheral lymphadenopathy	26 (63)	99 (64)	90 (63)
	Unknown	1 (2)	3 (2)	4 (3)

Variable	Value	Confirmed TB (N=41)	Unconfirmed TB (N=155)	Unlikely TB (N=142)
		n (%) OR median (IQR)	n (%) OR median (IQR)	n (%) OR median (IQR)
First Xpert MTB/RIF	Xpert positive for Mtb	21 (51)	0 (0)	0 (0)
	Xpert negative for Mtb	19 (46)	154 (99)	132 (93)
	Unknown/not performed	1 (2)	1 (1)	10 (7)
Overall CXR read	CXR consistent with TB	27 (66)	112 (72)	65 (46)
	CXR not consistent with TB	12 (29)	39 (25)	66 (46)
	Unknown/not assessed	2 (5)	4 (3)	11 (8)
Opacities on CXR	Opacities present on CXR	15 (37)	62 (40)	35 (25)
	Opacities not present on CXR	24 (59)	89 (57)	95 (67)
	Unknown/not assessed	2 (5)	4 (3)	12 (8)
Cavities on CXR	Cavities present on CXR	1 (2)	6 (4)	0 (0)
	Cavities not present on CXR	38 (93)	145 (94)	129 (91)
	Unknown/not assessed	2 (5)	4 (3)	13 (9)
Miliary infiltrate on CXR	Miliary infiltrate present on CXR	3 (7)	8 (5)	5 (4)
	Miliary infiltrate not present on CXR	36 (88)	143 (92)	125 (88)
	Unknown/not assessed	2 (5)	4 (3)	12 (8)
Intrathoracic lymphadenopathy on CXR	Intrathoracic lymphadenopathy present on CXR	5 (12)	67 (43)	17 (12)
	Intrathoracic lymphadenopathy not present on CXR	34 (83)	84 (54)	113 (80)
	Unknown/not assessed	2 (5)	4 (3)	12 (8)
Pleural effusion on CXR	Pleural effusion present on CXR	0 (0)	7 (5)	5 (4)
	Pleural effusion not present on CXR	39 (95)	144 (93)	125 (88)
	Unknown/not assessed	2 (5)	4 (3)	12 (8)
Tuberculin skin test	TST positive	7 (17)	10 (6)	8 (6)
	TST negative	29 (71)	133 (86)	114 (80)
	Unknown/not performed	5 (12)	12 (8)	20 (14)

Table S35. Description of IPD from Hamid/2019/PK

Variable	Value	Confirmed TB (N=0)	Unconfirmed TB (N=29)	Unlikely TB (N=416)
		n (%) OR median (IQR)	n (%) OR median (IQR)	n (%) OR median (IQR)
Age	Months	NA (NA,NA)	66 (30,90)	66 (42,90)
	Unknown	0 (NaN)	0 (0)	0 (0)
Sex	Male	0 (NaN)	12 (41)	222 (53)
	Female	0 (NaN)	17 (59)	194 (47)
	Unknown	0 (NaN)	0 (0)	0 (0)
Weight-for-age	z-score	NA (NA,NA)	-2.4 (-3.5,-1.6)	-2.2 (-3.2,-1.5)
	Unknown	0 (NaN)	0 (0)	83 (20)
BCG vaccination	Evidence of BCG vaccination	0 (NaN)	0 (0)	0 (0)
	No evidence of BCG vaccination	0 (NaN)	0 (0)	0 (0)
	Unknown	0 (NaN)	29 (100)	416 (100)
HIV status	HIV-positive	0 (NaN)	0 (0)	0 (0)
	HIV-negative	0 (NaN)	29 (100)	416 (100)
	Unknown	0 (NaN)	0 (0)	0 (0)
Cough duration	No cough	0 (NaN)	5 (17)	175 (42)
	Cough 0-13 days	0 (NaN)	1 (3)	60 (14)
	Cough 14-20 days	0 (NaN)	17 (59)	46 (11)
	Cough 21-27 days	0 (NaN)	6 (21)	67 (16)
	Cough >27 days	0 (NaN)	0 (0)	64 (15)
	Unknown	0 (NaN)	0 (0)	4 (1)
Cough ≥2 weeks	Cough ≥2 weeks present	0 (NaN)	23 (79)	177 (43)
	Cough ≥2 weeks not present	0 (NaN)	6 (21)	235 (56)
	Unknown	0 (NaN)	0 (0)	4 (1)
Fever duration	No fever	0 (NaN)	7 (24)	152 (37)
	Fever 0-13 days	0 (NaN)	11 (38)	157 (38)
	Fever 14-20 days	0 (NaN)	10 (34)	66 (16)
	Fever 21-27 days	0 (NaN)	1 (3)	27 (6)
	Fever >27 days	0 (NaN)	0 (0)	6 (1)
	Unknown	0 (NaN)	0 (0)	8 (2)
Fever ≥1 week	Fever ≥1 week present	0 (NaN)	11 (38)	99 (24)
	Fever ≥1 week not present	0 (NaN)	18 (62)	309 (74)
	Unknown	0 (NaN)	0 (0)	8 (2)
Lethargy	Lethargy	0 (NaN)	2 (7)	6 (1)
	No lethargy	0 (NaN)	27 (93)	410 (99)
	Unknown	0 (NaN)	0 (0)	0 (0)
Weight loss	Weight loss	0 (NaN)	14 (48)	58 (14)
	No weight loss	0 (NaN)	15 (52)	358 (86)
	Unknown	0 (NaN)	0 (0)	0 (0)
Documented TB exposure	Documented TB exposure in previous 12 months	0 (NaN)	13 (45)	16 (4)
	No documented TB exposure in previous 12 months	0 (NaN)	16 (55)	400 (96)
	Unknown	0 (NaN)	0 (0)	0 (0)
Night sweats	Night sweats	0 (NaN)	2 (7)	11 (3)
	No night sweats	0 (NaN)	27 (93)	405 (97)
	Unknown	0 (NaN)	0 (0)	0 (0)
Haemoptysis	Haemoptysis	0 (NaN)	0 (0)	1 (0)
	No haemoptysis	0 (NaN)	29 (100)	415 (100)
	Unknown	0 (NaN)	0 (0)	0 (0)
Objective fever (≥38 degrees Celsius)	Objective fever	0 (NaN)	0 (0)	0 (0)
	No objective fever	0 (NaN)	0 (0)	0 (0)
	Unknown	0 (NaN)	29 (100)	416 (100)
Tachycardia	Tachycardia	0 (NaN)	0 (0)	0 (0)
	No tachycardia	0 (NaN)	0 (0)	0 (0)
	Unknown	0 (NaN)	29 (100)	416 (100)
Tachypnoea	Tachypnoea	0 (NaN)	0 (0)	0 (0)
	No tachypnoea	0 (NaN)	0 (0)	0 (0)
	Unknown	0 (NaN)	29 (100)	416 (100)
Peripheral lymphadenopathy	Peripheral lymphadenopathy	0 (NaN)	2 (7)	34 (8)
	No peripheral lymphadenopathy	0 (NaN)	27 (93)	382 (92)
	Unknown	0 (NaN)	0 (0)	0 (0)

Variable	Value	Confirmed TB (N=0)	Unconfirmed TB (N=29)	Unlikely TB (N=416)
		n (%) OR median (IQR)	n (%) OR median (IQR)	n (%) OR median (IQR)
First Xpert MTB/RIF	Xpert positive for Mtb	0 (NaN)	0 (0)	0 (0)
	Xpert negative for Mtb	0 (NaN)	7 (24)	156 (38)
	Unknown/not performed	0 (NaN)	22 (76)	260 (62)
Overall CXR read	CXR consistent with TB	0 (NaN)	20 (69)	29 (7)
	CXR not consistent with TB	0 (NaN)	4 (14)	278 (67)
	Unknown/not assessed	0 (NaN)	5 (17)	109 (26)
Opacities on CXR	Opacities present on CXR	0 (NaN)	0 (0)	0 (0)
	Opacities not present on CXR	0 (NaN)	0 (0)	0 (0)
	Unknown/not assessed	0 (NaN)	29 (100)	416 (100)
Cavities on CXR	Cavities present on CXR	0 (NaN)	0 (0)	0 (0)
	Cavities not present on CXR	0 (NaN)	0 (0)	0 (0)
	Unknown/not assessed	0 (NaN)	29 (100)	416 (100)
Miliary infiltrate on CXR	Miliary infiltrate present on CXR	0 (NaN)	0 (0)	0 (0)
	Miliary infiltrate not present on CXR	0 (NaN)	0 (0)	0 (0)
	Unknown/not assessed	0 (NaN)	29 (100)	416 (100)
Intrathoracic lymphadenopathy on CXR	Intrathoracic lymphadenopathy present on CXR	0 (NaN)	0 (0)	0 (0)
	Intrathoracic lymphadenopathy not present on CXR	0 (NaN)	0 (0)	0 (0)
	Unknown/not assessed	0 (NaN)	29 (100)	416 (100)
Pleural effusion on CXR	Pleural effusion present on CXR	0 (NaN)	0 (0)	0 (0)
	Pleural effusion not present on CXR	0 (NaN)	0 (0)	0 (0)
	Unknown/not assessed	0 (NaN)	29 (100)	416 (100)
Tuberculin skin test	TST positive	0 (NaN)	10 (34)	82 (20)
	TST negative	0 (NaN)	0 (0)	4 (1)
	Unknown/not performed	0 (NaN)	19 (66)	330 (79)

Table S36. Description of IPD from Zar/2019/ZA

Variable	Value	Confirmed TB (N=189)	Unconfirmed TB (N=274)	Unlikely TB (N=303)
		n (%) OR median (IQR)	n (%) OR median (IQR)	n (%) OR median (IQR)
Age	Months	32·4 (17,61·6)	25·1 (11·9,53·9)	24 (12·4,50·5)
	Unknown	0 (0)	0 (0)	0 (0)
Sex	Male	111 (59)	155 (57)	156 (51)
	Female	78 (41)	119 (43)	147 (49)
	Unknown	0 (0)	0 (0)	0 (0)
Weight-for-age	z-score	-1.3 (-2.2,-0.2)	-0.9 (-1.7,0)	-0.8 (-1.9,0.1)
	Unknown	0 (0)	2 (1)	1 (0)
BCG vaccination	Evidence of BCG vaccination	163 (86)	252 (92)	281 (93)
	No evidence of BCG vaccination	0 (0)	2 (1)	1 (0)
	Unknown	26 (14)	20 (7)	21 (7)
HIV status	HIV-positive	29 (15)	57 (21)	51 (17)
	HIV-negative	160 (85)	217 (79)	252 (83)
	Unknown	0 (0)	0 (0)	0 (0)
Cough duration	No cough	33 (17)	62 (23)	47 (16)
	Cough 0-13 days	71 (38)	109 (40)	134 (44)
	Cough 14-20 days	37 (20)	33 (12)	47 (16)
	Cough 21-27 days	16 (8)	12 (4)	16 (5)
	Cough >27 days	27 (14)	51 (19)	48 (16)
	Unknown	5 (3)	7 (3)	11 (4)
Cough ≥2 weeks	Cough ≥2 weeks present	80 (42)	96 (35)	111 (37)
	Cough ≥2 weeks not present	104 (55)	171 (62)	181 (60)
	Unknown	5 (3)	7 (3)	11 (4)
Fever duration	No fever	72 (38)	103 (38)	106 (35)
	Fever 0-13 days	70 (37)	118 (43)	145 (48)
	Fever 14-20 days	22 (12)	21 (8)	17 (6)
	Fever 21-27 days	5 (3)	12 (4)	9 (3)
	Fever >27 days	15 (8)	13 (5)	13 (4)
	Unknown	5 (3)	7 (3)	13 (4)
Fever ≥1 week	Fever ≥1 week present	66 (35)	85 (31)	87 (29)
	Fever ≥1 week not present	118 (62)	182 (66)	203 (67)
	Unknown	5 (3)	7 (3)	13 (4)
Lethargy	Lethargy	91 (48)	124 (45)	124 (41)
	No lethargy	93 (49)	138 (50)	163 (54)
	Unknown	5 (3)	12 (4)	16 (5)
Weight loss	Weight loss	146 (77)	182 (66)	206 (68)
	No weight loss	41 (22)	85 (31)	88 (29)
	Unknown	2 (1)	7 (3)	9 (3)
Documented TB exposure	Documented TB exposure in previous 12 months	113 (60)	160 (58)	184 (61)
	No documented TB exposure in previous 12 months	76 (40)	114 (42)	119 (39)
	Unknown	0 (0)	0 (0)	0 (0)
Night sweats	Night sweats	109 (58)	149 (54)	131 (43)
	No night sweats	79 (42)	121 (44)	164 (54)
	Unknown	1 (1)	4 (1)	8 (3)
Haemoptysis	Haemoptysis	0 (0)	0 (0)	0 (0)
	No haemoptysis	0 (0)	0 (0)	0 (0)
	Unknown	189 (100)	274 (100)	303 (100)
Objective fever (≥38 degrees Celsius)	Objective fever	42 (22)	48 (18)	52 (17)
	No objective fever	145 (77)	220 (80)	243 (80)
	Unknown	2 (1)	6 (2)	8 (3)
Tachycardia	Tachycardia	51 (27)	85 (31)	87 (29)
	No tachycardia	129 (68)	171 (62)	203 (67)
	Unknown	9 (5)	18 (7)	13 (4)
Tachypnoea	Tachypnoea	61 (32)	92 (34)	116 (38)
	No tachypnoea	120 (63)	166 (61)	178 (59)
	Unknown	8 (4)	16 (6)	9 (3)
Peripheral lymphadenopathy	Peripheral lymphadenopathy	80 (42)	107 (39)	91 (30)
	No peripheral lymphadenopathy	106 (56)	157 (57)	205 (68)
	Unknown	3 (2)	10 (4)	7 (2)

Variable	Value	Confirmed TB (N=189)	Unconfirmed TB (N=274)	Unlikely TB (N=303)
		n (%) OR median (IQR)	n (%) OR median (IQR)	n (%) OR median (IQR)
First Xpert MTB/RIF	Xpert positive for Mtb	98 (52)	0 (0)	0 (0)
	Xpert negative for Mtb	91 (48)	271 (99)	303 (100)
	Unknown/not performed	0 (0)	3 (1)	0 (0)
Overall CXR read	CXR consistent with TB	95 (50)	104 (38)	66 (22)
	CXR not consistent with TB	17 (9)	76 (28)	94 (31)
	Unknown/not assessed	77 (41)	94 (34)	143 (47)
Opacities on CXR	Opacities present on CXR	130 (69)	165 (60)	194 (64)
	Opacities not present on CXR	59 (31)	109 (40)	108 (36)
	Unknown/not assessed	0 (0)	0 (0)	1 (0)
Cavities on CXR	Cavities present on CXR	21 (11)	6 (2)	12 (4)
	Cavities not present on CXR	168 (89)	268 (98)	291 (96)
	Unknown/not assessed	0 (0)	0 (0)	0 (0)
Miliary infiltrate on CXR	Miliary infiltrate present on CXR	21 (11)	2 (1)	2 (1)
	Miliary infiltrate not present on CXR	168 (89)	272 (99)	301 (99)
	Unknown/not assessed	0 (0)	0 (0)	0 (0)
Intrathoracic lymphadenopathy on CXR	Intrathoracic lymphadenopathy present on CXR	75 (40)	94 (34)	56 (18)
	Intrathoracic lymphadenopathy not present on CXR	114 (60)	180 (66)	246 (81)
	Unknown/not assessed	0 (0)	0 (0)	1 (0)
Pleural effusion on CXR	Pleural effusion present on CXR	44 (23)	39 (14)	23 (8)
	Pleural effusion not present on CXR	145 (77)	235 (86)	278 (92)
	Unknown/not assessed	0 (0)	0 (0)	2 (1)
Tuberculin skin test	TST positive	138 (73)	173 (63)	42 (14)
	TST negative	32 (17)	81 (30)	230 (76)
	Unknown/not performed	19 (10)	20 (7)	31 (10)

Table S37. Description of IPD from Walters/2017/ZA

Variable	Value	Confirmed TB (N=119)	Unconfirmed TB (N=180)	Unlikely TB (N=283)	TB-status unknown (N=13)
		n (%) OR median (IQR)	n (%) OR median (IQR)	n (%) OR median (IQR)	n (%) OR median (IQR)
Age	Months	18·8 (9·7,44·7)	16·4 (10·5,29·3)	15·1 (9·1,28·4)	11·5 (7·1,22·6)
	Unknown	0 (0)	0 (0)	0 (0)	0 (0)
Sex	Male	53 (45)	106 (59)	153 (54)	8 (62)
	Female	66 (55)	74 (41)	130 (46)	5 (38)
	Unknown	0 (0)	0 (0)	0 (0)	0 (0)
Weight-for-age	z-score	-1.5 (-2.6,-0.7)	-1.2 (-2.4,-0.2)	-1.2 (-2,-0.3)	-1.6 (-2.3,-0.6)
	Unknown	0 (0)	0 (0)	0 (0)	0 (0)
BCG vaccination	Evidence of BCG vaccination	108 (91)	179 (99)	278 (98)	13 (100)
	No evidence of BCG vaccination	11 (9)	1 (1)	5 (2)	0 (0)
	Unknown	0 (0)	0 (0)	0 (0)	0 (0)
HIV status	HIV-positive	16 (13)	32 (18)	21 (7)	1 (8)
	HIV-negative	103 (87)	148 (82)	262 (93)	12 (92)
	Unknown	0 (0)	0 (0)	0 (0)	0 (0)
Cough duration	No cough	18 (15)	35 (19)	64 (23)	13 (100)
	Cough 0-13 days	43 (36)	65 (36)	120 (42)	0 (0)
	Cough 14-20 days	24 (20)	22 (12)	35 (12)	0 (0)
	Cough 21-27 days	8 (7)	13 (7)	18 (6)	0 (0)
	Cough >27 days	26 (22)	44 (24)	41 (14)	0 (0)
	Unknown	0 (0)	1 (1)	5 (2)	0 (0)
Cough ≥2 weeks	Cough ≥2 weeks present	58 (49)	79 (44)	94 (33)	0 (0)
	Cough ≥2 weeks not present	61 (51)	100 (56)	184 (65)	13 (100)
	Unknown	0 (0)	1 (1)	5 (2)	0 (0)
Fever duration	No fever	45 (38)	78 (43)	148 (52)	4 (31)
	Fever 0-13 days	26 (22)	32 (18)	61 (22)	2 (15)
	Fever 14-20 days	3 (3)	1 (1)	4 (1)	2 (15)
	Fever 21-27 days	2 (2)	1 (1)	2 (1)	0 (0)
	Fever >27 days	3 (3)	6 (3)	5 (2)	0 (0)
	Unknown	40 (34)	62 (34)	63 (22)	5 (38)
Fever ≥1 week	Fever ≥1 week present	15 (13)	16 (9)	19 (7)	3 (23)
	Fever ≥1 week not present	64 (54)	102 (57)	201 (71)	5 (38)
	Unknown	40 (34)	62 (34)	63 (22)	5 (38)
Lethargy	Lethargy	51 (43)	80 (44)	97 (34)	6 (46)
	No lethargy	68 (57)	100 (56)	186 (66)	7 (54)
	Unknown	0 (0)	0 (0)	0 (0)	0 (0)
Weight loss	Weight loss	55 (46)	83 (46)	109 (39)	7 (54)
	No weight loss	64 (54)	97 (54)	174 (61)	6 (46)
	Unknown	0 (0)	0 (0)	0 (0)	0 (0)
Documented TB exposure	Documented TB exposure in previous 12 months	61 (51)	93 (52)	61 (22)	5 (38)
	No documented TB exposure in previous 12 months	58 (49)	87 (48)	222 (78)	8 (62)
	Unknown	0 (0)	0 (0)	0 (0)	0 (0)
Night sweats	Night sweats	0 (0)	0 (0)	0 (0)	0 (0)
	No night sweats	0 (0)	0 (0)	0 (0)	0 (0)
	Unknown	119 (100)	180 (100)	283 (100)	13 (100)
Haemoptysis	Haemoptysis	0 (0)	0 (0)	0 (0)	0 (0)
	No haemoptysis	0 (0)	0 (0)	0 (0)	0 (0)
	Unknown	119 (100)	180 (100)	283 (100)	13 (100)
Objective fever (≥38 degrees Celsius)	Objective fever	0 (0)	0 (0)	0 (0)	0 (0)
	No objective fever	0 (0)	0 (0)	0 (0)	0 (0)
	Unknown	119 (100)	180 (100)	283 (100)	13 (100)
Tachycardia	Tachycardia	0 (0)	0 (0)	0 (0)	0 (0)
	No tachycardia	0 (0)	0 (0)	0 (0)	0 (0)
	Unknown	119 (100)	180 (100)	283 (100)	13 (100)
Tachypnoea	Tachypnoea	39 (33)	61 (34)	79 (28)	4 (31)
	No tachypnoea	78 (66)	116 (64)	202 (71)	9 (69)
	Unknown	2 (2)	3 (2)	2 (1)	0 (0)
Peripheral lymphadenopathy	Peripheral lymphadenopathy	34 (29)	51 (28)	56 (20)	1 (8)
	No peripheral lymphadenopathy	85 (71)	129 (72)	227 (80)	12 (92)
	Unknown	0 (0)	0 (0)	0 (0)	0 (0)

Variable	Value	Confirmed TB (N=119)	Unconfirmed TB (N=180)	Unlikely TB (N=283)	TB-status unknown (N=13)
		n (%) OR median (IQR)	n (%) OR median (IQR)	n (%) OR median (IQR)	n (%) OR median (IQR)
First Xpert MTB/RIF	Xpert positive for Mtb	37 (31)	0 (0)	0 (0)	0 (0)
	Xpert negative for Mtb	69 (58)	141 (78)	233 (82)	11 (85)
	Unknown/not performed	13 (11)	39 (22)	50 (18)	2 (15)
Overall CXR read	CXR consistent with TB	83 (70)	61 (34)	25 (9)	2 (15)
	CXR not consistent with TB	29 (24)	105 (58)	235 (83)	11 (85)
	Unknown/not assessed	7 (6)	14 (8)	23 (8)	0 (0)
Opacities on CXR	Opacities present on CXR	60 (50)	74 (41)	126 (45)	6 (46)
	Opacities not present on CXR	59 (50)	106 (59)	157 (55)	7 (54)
	Unknown/not assessed	0 (0)	0 (0)	0 (0)	0 (0)
Cavities on CXR	Cavities present on CXR	9 (8)	5 (3)	6 (2)	0 (0)
	Cavities not present on CXR	110 (92)	175 (97)	277 (98)	13 (100)
	Unknown/not assessed	0 (0)	0 (0)	0 (0)	0 (0)
Miliary infiltrate on CXR	Miliary infiltrate present on CXR	6 (5)	3 (2)	0 (0)	0 (0)
	Miliary infiltrate not present on CXR	113 (95)	177 (98)	283 (100)	13 (100)
	Unknown/not assessed	0 (0)	0 (0)	0 (0)	0 (0)
Intrathoracic lymphadenopathy on CXR	Intrathoracic lymphadenopathy present on CXR	69 (58)	50 (28)	14 (5)	1 (8)
	Intrathoracic lymphadenopathy not present on CXR	50 (42)	130 (72)	269 (95)	12 (92)
	Unknown/not assessed	0 (0)	0 (0)	0 (0)	0 (0)
Pleural effusion on CXR	Pleural effusion present on CXR	8 (7)	4 (2)	7 (2)	0 (0)
	Pleural effusion not present on CXR	111 (93)	176 (98)	276 (98)	13 (100)
	Unknown/not assessed	0 (0)	0 (0)	0 (0)	0 (0)
Tuberculin skin test	TST positive	63 (53)	48 (27)	14 (5)	3 (23)
	TST negative	26 (22)	95 (53)	200 (71)	8 (62)
	Unknown/not performed	30 (25)	37 (21)	69 (24)	2 (15)

Table S38. Description of IPD from Orikiriza/2018/UG

Variable	Value	Confirmed TB (N=12)	Unconfirmed TB (N=145)	Unlikely TB (N=167)	TB-status unknown (N=14)
		n (%) OR median (IQR)	n (%) OR median (IQR)	n (%) OR median (IQR)	n (%) OR median (IQR)
Age	Months	31 (20·8,66)	28 (12,60)	47 (16,72)	20 (10·5,66)
	Unknown	0 (0)	0 (0)	0 (0)	0 (0)
Sex	Male	9 (75)	82 (57)	91 (54)	5 (36)
	Female	3 (25)	63 (43)	76 (46)	9 (64)
	Unknown	0 (0)	0 (0)	0 (0)	0 (0)
Weight-for-age	z-score	-2 (-3,-0.9)	-1.5 (-2.9,-0.4)	-1.2 (-2.6,-0.2)	-1.9 (-2.9,-1.1)
	Unknown	0 (0)	0 (0)	0 (0)	0 (0)
BCG vaccination	Evidence of BCG vaccination	10 (83)	123 (85)	151 (90)	11 (79)
	No evidence of BCG vaccination	1 (8)	4 (3)	3 (2)	1 (7)
	Unknown	1 (8)	18 (12)	13 (8)	2 (14)
HIV status	HIV-positive	5 (42)	43 (30)	51 (31)	2 (14)
	HIV-negative	6 (50)	100 (69)	115 (69)	12 (86)
	Unknown	1 (8)	2 (1)	1 (1)	0 (0)
Cough duration	No cough	0 (0)	0 (0)	0 (0)	0 (0)
	Cough 0-13 days	0 (0)	0 (0)	0 (0)	0 (0)
	Cough 14-20 days	0 (0)	0 (0)	0 (0)	0 (0)
	Cough 21-27 days	0 (0)	0 (0)	0 (0)	0 (0)
	Cough >27 days	0 (0)	0 (0)	0 (0)	0 (0)
	Unknown	12 (100)	145 (100)	167 (100)	14 (100)
Cough ≥2 weeks	Cough ≥2 weeks present	12 (100)	129 (89)	161 (96)	10 (71)
	Cough ≥2 weeks not present	0 (0)	16 (11)	6 (4)	4 (29)
	Unknown	0 (0)	0 (0)	0 (0)	0 (0)
Fever duration	No fever	0 (0)	0 (0)	0 (0)	0 (0)
	Fever 0-13 days	0 (0)	0 (0)	0 (0)	0 (0)
	Fever 14-20 days	0 (0)	0 (0)	0 (0)	0 (0)
	Fever 21-27 days	0 (0)	0 (0)	0 (0)	0 (0)
	Fever >27 days	0 (0)	0 (0)	0 (0)	0 (0)
	Unknown	12 (100)	145 (100)	167 (100)	14 (100)
Fever ≥1 week	Fever ≥1 week present	7 (58)	59 (41)	54 (32)	6 (43)
	Fever ≥1 week not present	5 (42)	86 (59)	113 (68)	8 (57)
	Unknown	0 (0)	0 (0)	0 (0)	0 (0)
Lethargy	Lethargy	7 (58)	53 (37)	49 (29)	6 (43)
	No lethargy	5 (42)	92 (63)	118 (71)	8 (57)
	Unknown	0 (0)	0 (0)	0 (0)	0 (0)
Weight loss	Weight loss	8 (67)	73 (50)	90 (54)	9 (64)
	No weight loss	4 (33)	72 (50)	77 (46)	5 (36)
	Unknown	0 (0)	0 (0)	0 (0)	0 (0)
Documented TB exposure	Documented TB exposure in previous 12 months	4 (33)	58 (40)	3 (2)	5 (36)
	No documented TB exposure in previous 12 months	8 (67)	86 (59)	164 (98)	9 (64)
	Unknown	0 (0)	1 (1)	0 (0)	0 (0)
Night sweats	Night sweats	3 (25)	44 (30)	42 (25)	2 (14)
	No night sweats	9 (75)	100 (69)	123 (74)	11 (79)
	Unknown	0 (0)	1 (1)	2 (1)	1 (7)
Haemoptysis	Haemoptysis	0 (0)	2 (1)	3 (2)	0 (0)
	No haemoptysis	12 (100)	142 (98)	162 (97)	13 (93)
	Unknown	0 (0)	1 (1)	2 (1)	1 (7)
Objective fever (≥38 degrees Celsius)	Objective fever	2 (17)	5 (3)	8 (5)	0 (0)
	No objective fever	10 (83)	140 (97)	159 (95)	14 (100)
	Unknown	0 (0)	0 (0)	0 (0)	0 (0)
Tachycardia	Tachycardia	4 (33)	16 (11)	23 (14)	3 (21)
	No tachycardia	8 (67)	127 (88)	143 (86)	11 (79)
	Unknown	0 (0)	2 (1)	1 (1)	0 (0)
Tachypnoea	Tachypnoea	3 (25)	33 (23)	38 (23)	2 (14)
	No tachypnoea	9 (75)	111 (77)	129 (77)	12 (86)
	Unknown	0 (0)	1 (1)	0 (0)	0 (0)
Peripheral lymphadenopathy	Peripheral lymphadenopathy	0 (0)	3 (2)	4 (2)	0 (0)
	No peripheral lymphadenopathy	12 (100)	139 (96)	160 (96)	12 (86)
	Unknown	0 (0)	3 (2)	3 (2)	2 (14)

Variable	Value	Confirmed TB (N=12)	Unconfirmed TB (N=145)	Unlikely TB (N=167)	TB-status unknown (N=14)
		n (%) OR median (IQR)	n (%) OR median (IQR)	n (%) OR median (IQR)	n (%) OR median (IQR)
First Xpert MTB/RIF	Xpert positive for Mtb	9 (75)	0 (0)	0 (0)	0 (0)
	Xpert negative for Mtb	1 (8)	124 (86)	151 (90)	10 (71)
	Unknown/not performed	2 (17)	21 (14)	16 (10)	4 (29)
Overall CXR read	CXR consistent with TB	7 (58)	84 (58)	26 (16)	7 (50)
	CXR not consistent with TB	5 (42)	57 (39)	140 (84)	4 (29)
	Unknown/not assessed	0 (0)	4 (3)	1 (1)	3 (21)
Opacities on CXR	Opacities present on CXR	10 (83)	81 (56)	61 (37)	7 (50)
	Opacities not present on CXR	2 (17)	60 (41)	105 (63)	4 (29)
	Unknown/not assessed	0 (0)	4 (3)	1 (1)	3 (21)
Cavities on CXR	Cavities present on CXR	1 (8)	3 (2)	0 (0)	0 (0)
	Cavities not present on CXR	11 (92)	138 (95)	166 (99)	11 (79)
	Unknown/not assessed	0 (0)	4 (3)	1 (1)	3 (21)
Miliary infiltrate on CXR	Miliary infiltrate present on CXR	0 (0)	2 (1)	0 (0)	0 (0)
	Miliary infiltrate not present on CXR	12 (100)	139 (96)	166 (99)	11 (79)
	Unknown/not assessed	0 (0)	4 (3)	1 (1)	3 (21)
Intrathoracic lymphadenopathy on CXR	Intrathoracic lymphadenopathy present on CXR	1 (8)	14 (10)	7 (4)	1 (7)
	Intrathoracic lymphadenopathy not present on CXR	11 (92)	131 (90)	160 (96)	13 (93)
	Unknown/not assessed	0 (0)	0 (0)	0 (0)	0 (0)
Pleural effusion on CXR	Pleural effusion present on CXR	1 (8)	1 (1)	3 (2)	0 (0)
	Pleural effusion not present on CXR	11 (92)	140 (97)	163 (98)	11 (79)
	Unknown/not assessed	0 (0)	4 (3)	1 (1)	3 (21)
Tuberculin skin test	TST positive	7 (58)	72 (50)	1 (1)	4 (29)
	TST negative	5 (42)	71 (49)	162 (97)	8 (57)
	Unknown/not performed	0 (0)	2 (1)	4 (2)	2 (14)

Table S39. Description of IPD from Orikiriza/2022/UG

Variable	Value	Confirmed TB (N=12)	Unconfirmed TB (N=58)	Unlikely TB (N=125)	TB-status unknown (N=22)
		n (%) OR median (IQR)	n (%) OR median (IQR)	n (%) OR median (IQR)	n (%) OR median (IQR)
Age	Months	10·6 (5·1,31·2)	17·8 (12·3,37·6)	15 (9,22·9)	23·3 (10·9,39·8)
	Unknown	0 (0)	0 (0)	0 (0)	0 (0)
Sex	Male	6 (50)	24 (41)	68 (54)	12 (55)
	Female	6 (50)	34 (59)	57 (46)	10 (45)
	Unknown	0 (0)	0 (0)	0 (0)	0 (0)
Weight-for-age	z-score	-4.9 (-5.8,-2.9)	-3.6 (-5,-2.6)	-4.2 (-5.2,-2.8)	-4.2 (-5.2,-2.3)
	Unknown	0 (0)	0 (0)	0 (0)	1 (5)
BCG vaccination	Evidence of BCG vaccination	12 (100)	53 (91)	118 (94)	19 (86)
	No evidence of BCG vaccination	0 (0)	5 (9)	7 (6)	3 (14)
	Unknown	0 (0)	0 (0)	0 (0)	0 (0)
HIV status	HIV-positive	4 (33)	17 (29)	37 (30)	12 (55)
	HIV-negative	8 (67)	41 (71)	82 (66)	10 (45)
	Unknown	0 (0)	0 (0)	6 (5)	0 (0)
Cough duration	No cough	0 (0)	0 (0)	0 (0)	0 (0)
	Cough 0-13 days	0 (0)	0 (0)	0 (0)	0 (0)
	Cough 14-20 days	0 (0)	0 (0)	0 (0)	0 (0)
	Cough 21-27 days	0 (0)	0 (0)	0 (0)	0 (0)
	Cough >27 days	0 (0)	0 (0)	0 (0)	0 (0)
	Unknown	12 (100)	58 (100)	125 (100)	22 (100)
Cough ≥2 weeks	Cough ≥2 weeks present	2 (17)	7 (12)	15 (12)	2 (9)
	Cough ≥2 weeks not present	9 (75)	50 (86)	102 (82)	20 (91)
	Unknown	1 (8)	1 (2)	8 (6)	0 (0)
Fever duration	No fever	0 (0)	0 (0)	0 (0)	0 (0)
	Fever 0-13 days	0 (0)	0 (0)	0 (0)	0 (0)
	Fever 14-20 days	0 (0)	0 (0)	0 (0)	0 (0)
	Fever 21-27 days	0 (0)	0 (0)	0 (0)	0 (0)
	Fever >27 days	0 (0)	0 (0)	0 (0)	0 (0)
	Unknown	12 (100)	58 (100)	125 (100)	22 (100)
Fever ≥1 week	Fever ≥1 week present	3 (25)	11 (19)	28 (22)	6 (27)
	Fever ≥1 week not present	8 (67)	43 (74)	81 (65)	14 (64)
	Unknown	1 (8)	4 (7)	16 (13)	2 (9)
Lethargy	Lethargy	12 (100)	55 (95)	120 (96)	21 (95)
	No lethargy	0 (0)	3 (5)	5 (4)	1 (5)
	Unknown	0 (0)	0 (0)	0 (0)	0 (0)
Weight loss	Weight loss	11 (92)	48 (83)	106 (85)	16 (73)
	No weight loss	1 (8)	10 (17)	19 (15)	6 (27)
	Unknown	0 (0)	0 (0)	0 (0)	0 (0)
Documented TB exposure	Documented TB exposure in previous 12 months	3 (25)	15 (26)	0 (0)	0 (0)
	No documented TB exposure in previous 12 months	9 (75)	40 (69)	121 (97)	22 (100)
	Unknown	0 (0)	3 (5)	4 (3)	0 (0)
Night sweats	Night sweats	4 (33)	24 (41)	34 (27)	5 (23)
	No night sweats	8 (67)	34 (59)	91 (73)	17 (77)
	Unknown	0 (0)	0 (0)	0 (0)	0 (0)
Haemoptysis	Haemoptysis	0 (0)	0 (0)	0 (0)	0 (0)
	No haemoptysis	0 (0)	0 (0)	0 (0)	0 (0)
	Unknown	12 (100)	58 (100)	125 (100)	22 (100)
Objective fever (≥38 degrees Celsius)	Objective fever	2 (17)	10 (17)	27 (22)	7 (32)
	No objective fever	10 (83)	48 (83)	98 (78)	14 (64)
	Unknown	0 (0)	0 (0)	0 (0)	1 (5)
Tachycardia	Tachycardia	9 (75)	25 (43)	44 (35)	15 (68)
	No tachycardia	3 (25)	33 (57)	80 (64)	6 (27)
	Unknown	0 (0)	0 (0)	1 (1)	1 (5)
Tachypnoea	Tachypnoea	6 (50)	15 (26)	20 (16)	8 (36)
	No tachypnoea	6 (50)	43 (74)	105 (84)	13 (59)
	Unknown	0 (0)	0 (0)	0 (0)	1 (5)
Peripheral lymphadenopathy	Peripheral lymphadenopathy	5 (42)	15 (26)	25 (20)	6 (27)
	No peripheral lymphadenopathy	7 (58)	43 (74)	100 (80)	16 (73)
	Unknown	0 (0)	0 (0)	0 (0)	0 (0)

Variable	Value	Confirmed TB (N=12)	Unconfirmed TB (N=58)	Unlikely TB (N=125)	TB-status unknown (N=22)
		n (%) OR median (IQR)	n (%) OR median (IQR)	n (%) OR median (IQR)	n (%) OR median (IQR)
First Xpert MTB/RIF	Xpert positive for Mtb	8 (67)	0 (0)	0 (0)	0 (0)
	Xpert negative for Mtb	4 (33)	58 (100)	124 (99)	17 (77)
	Unknown/not performed	0 (0)	0 (0)	1 (1)	5 (23)
Overall CXR read	CXR consistent with TB	4 (33)	10 (17)	4 (3)	1 (5)
	CXR not consistent with TB	6 (50)	48 (83)	121 (97)	6 (27)
	Unknown/not assessed	2 (17)	0 (0)	0 (0)	15 (68)
Opacities on CXR	Opacities present on CXR	6 (50)	38 (66)	57 (46)	4 (18)
	Opacities not present on CXR	4 (33)	11 (19)	30 (24)	1 (5)
	Unknown/not assessed	2 (17)	9 (16)	38 (30)	17 (77)
Cavities on CXR	Cavities present on CXR	0 (0)	2 (3)	1 (1)	0 (0)
	Cavities not present on CXR	10 (83)	47 (81)	86 (69)	5 (23)
	Unknown/not assessed	2 (17)	9 (16)	38 (30)	17 (77)
Miliary infiltrate on CXR	Miliary infiltrate present on CXR	2 (17)	0 (0)	0 (0)	1 (5)
	Miliary infiltrate not present on CXR	8 (67)	49 (84)	87 (70)	4 (18)
	Unknown/not assessed	2 (17)	9 (16)	38 (30)	17 (77)
Intrathoracic lymphadenopathy on CXR	Intrathoracic lymphadenopathy present on CXR	1 (8)	6 (10)	3 (2)	0 (0)
	Intrathoracic lymphadenopathy not present on CXR	9 (75)	43 (74)	84 (67)	5 (23)
	Unknown/not assessed	2 (17)	9 (16)	38 (30)	17 (77)
Pleural effusion on CXR	Pleural effusion present on CXR	0 (0)	2 (3)	0 (0)	0 (0)
	Pleural effusion not present on CXR	10 (83)	47 (81)	87 (70)	5 (23)
	Unknown/not assessed	2 (17)	9 (16)	38 (30)	17 (77)
Tuberculin skin test	TST positive	4 (33)	3 (5)	0 (0)	0 (0)
	TST negative	6 (50)	52 (90)	117 (94)	11 (50)
	Unknown/not performed	2 (17)	3 (5)	8 (6)	11 (50)

Table S40. Description of IPD from Giang/2015/VN

Variable	Value	Confirmed TB (N=20)	Unconfirmed TB (N=77)	Unlikely TB (N=16)
		n (%) OR median (IQR)	n (%) OR median (IQR)	n (%) OR median (IQR)
Age	Months	13 (7·8,23·8)	14 (9,20)	18 (9·8,44·5)
	Unknown	0 (0)	0 (0)	0 (0)
Sex	Male	14 (70)	55 (71)	9 (56)
	Female	6 (30)	22 (29)	7 (44)
	Unknown	0 (0)	0 (0)	0 (0)
Weight-for-age	z-score	-0.9 (-1.7,-0.1)	-0.7 (-1.7,0.2)	-1.3 (-2,-0.9)
	Unknown	0 (0)	0 (0)	0 (0)
BCG vaccination	Evidence of BCG vaccination	16 (80)	71 (92)	16 (100)
	No evidence of BCG vaccination	3 (15)	6 (8)	0 (0)
	Unknown	1 (5)	0 (0)	0 (0)
HIV status	HIV-positive	0 (0)	0 (0)	0 (0)
	HIV-negative	20 (100)	77 (100)	16 (100)
	Unknown	0 (0)	0 (0)	0 (0)
Cough duration	No cough	0 (0)	0 (0)	0 (0)
	Cough 0-13 days	0 (0)	0 (0)	0 (0)
	Cough 14-20 days	0 (0)	0 (0)	0 (0)
	Cough 21-27 days	0 (0)	0 (0)	0 (0)
	Cough >27 days	0 (0)	0 (0)	0 (0)
	Unknown	20 (100)	77 (100)	16 (100)
Cough ≥2 weeks	Cough ≥2 weeks present	14 (70)	65 (84)	14 (88)
	Cough ≥2 weeks not present	6 (30)	12 (16)	2 (12)
	Unknown	0 (0)	0 (0)	0 (0)
Fever duration	No fever	0 (0)	0 (0)	0 (0)
	Fever 0-13 days	0 (0)	0 (0)	0 (0)
	Fever 14-20 days	0 (0)	0 (0)	0 (0)
	Fever 21-27 days	0 (0)	0 (0)	0 (0)
	Fever >27 days	0 (0)	0 (0)	0 (0)
	Unknown	20 (100)	77 (100)	16 (100)
Fever ≥1 week	Fever ≥1 week present	11 (55)	43 (56)	9 (56)
	Fever ≥1 week not present	9 (45)	34 (44)	7 (44)
	Unknown	0 (0)	0 (0)	0 (0)
Lethargy	Lethargy	7 (35)	27 (35)	5 (31)
	No lethargy	13 (65)	50 (65)	11 (69)
	Unknown	0 (0)	0 (0)	0 (0)
Weight loss	Weight loss	9 (45)	33 (43)	5 (31)
	No weight loss	11 (55)	44 (57)	11 (69)
	Unknown	0 (0)	0 (0)	0 (0)
Documented TB exposure	Documented TB exposure in previous 12 months	6 (30)	9 (12)	1 (6)
	No documented TB exposure in previous 12 months	14 (70)	68 (88)	15 (94)
	Unknown	0 (0)	0 (0)	0 (0)
Night sweats	Night sweats	14 (70)	60 (78)	13 (81)
	No night sweats	6 (30)	17 (22)	3 (19)
	Unknown	0 (0)	0 (0)	0 (0)
Haemoptysis	Haemoptysis	1 (5)	0 (0)	0 (0)
	No haemoptysis	19 (95)	77 (100)	16 (100)
	Unknown	0 (0)	0 (0)	0 (0)
Objective fever (≥38 degrees Celsius)	Objective fever	0 (0)	0 (0)	0 (0)
	No objective fever	0 (0)	0 (0)	0 (0)
	Unknown	20 (100)	77 (100)	16 (100)
Tachycardia	Tachycardia	0 (0)	0 (0)	0 (0)
	No tachycardia	0 (0)	0 (0)	0 (0)
	Unknown	20 (100)	77 (100)	16 (100)
Tachypnoea	Tachypnoea	0 (0)	0 (0)	0 (0)
	No tachypnoea	0 (0)	0 (0)	0 (0)
	Unknown	20 (100)	77 (100)	16 (100)
Peripheral lymphadenopathy	Peripheral lymphadenopathy	0 (0)	1 (1)	0 (0)
	No peripheral lymphadenopathy	20 (100)	76 (99)	16 (100)
	Unknown	0 (0)	0 (0)	0 (0)

Variable	Value	Confirmed TB (N=20)	Unconfirmed TB (N=77)	Unlikely TB (N=16)
		n (%) OR median (IQR)	n (%) OR median (IQR)	n (%) OR median (IQR)
First Xpert MTB/RIF	Xpert positive for Mtb	7 (35)	0 (0)	0 (0)
	Xpert negative for Mtb	13 (65)	76 (99)	16 (100)
	Unknown/not performed	0 (0)	1 (1)	0 (0)
Overall CXR read	CXR consistent with TB	19 (95)	76 (99)	15 (94)
	CXR not consistent with TB	0 (0)	1 (1)	0 (0)
	Unknown/not assessed	1 (5)	0 (0)	1 (6)
Opacities on CXR	Opacities present on CXR	0 (0)	0 (0)	0 (0)
	Opacities not present on CXR	0 (0)	0 (0)	0 (0)
	Unknown/not assessed	20 (100)	77 (100)	16 (100)
Cavities on CXR	Cavities present on CXR	0 (0)	0 (0)	0 (0)
	Cavities not present on CXR	0 (0)	0 (0)	0 (0)
	Unknown/not assessed	20 (100)	77 (100)	16 (100)
Miliary infiltrate on CXR	Miliary infiltrate present on CXR	0 (0)	0 (0)	0 (0)
	Miliary infiltrate not present on CXR	0 (0)	0 (0)	0 (0)
	Unknown/not assessed	20 (100)	77 (100)	16 (100)
Intrathoracic lymphadenopathy on CXR	Intrathoracic lymphadenopathy present on CXR	0 (0)	0 (0)	0 (0)
	Intrathoracic lymphadenopathy not present on CXR	0 (0)	0 (0)	0 (0)
	Unknown/not assessed	20 (100)	77 (100)	16 (100)
Pleural effusion on CXR	Pleural effusion present on CXR	0 (0)	0 (0)	0 (0)
	Pleural effusion not present on CXR	0 (0)	0 (0)	0 (0)
	Unknown/not assessed	20 (100)	77 (100)	16 (100)
Tuberculin skin test	TST positive	18 (90)	60 (78)	9 (56)
	TST negative	1 (5)	12 (16)	5 (31)
	Unknown/not performed	1 (5)	5 (6)	2 (12)

Appendix L: Descriptions of entire IPD

Table S41. All clinical, bacteriology, and imaging data collected from the initial evaluation. Excluding data from 89 individuals with unknown TB status. IPD – Individual participant data, IQR – interquartile range, BCG – bacille Calmette-Guerin, HIV – human immunodeficiency virus, Mtb – *Mycobacterium tuberculosis*, CXR – chest x-ray, TST – tuberculin skin test.

**Variable**	**Value**	**Confirmed tuberculosis (N=541)**	**Unconfirmed tuberculosis (N=1 270)**	**Unlikely tuberculosis (N=2 818)**
		n (%) OR median (IQR)	n (%) OR median (IQR)	n (%) OR median (IQR)
Age	Months	25·6 (12,58·7)	24·1 (12,53·6)	24·4 (13·4,54)
	Unknown	0 (0)	0 (0)	0 (0)
Sex	Male	285 (53)	715 (56)	1512 (54)
	Female	256 (47)	554 (44)	1306 (46)
	Unknown	0 (0)	1 (0)	0 (0)
Weight-for-age	z-score	-1.7 (-2.9,-0.6)	-1.7 (-2.9,-0.6)	-1.8 (-3,-0.7)
	Unknown	7 (1)	16 (1)	119 (4)
BCG vaccination	Evidence of BCG vaccination	462 (85)	1 084 (85)	2 105 (75)
	No evidence of BCG vaccination	45 (8)	89 (7)	152 (5)
	Unknown	34 (6)	97 (8)	561 (20)
HIV status	HIV-positive	111 (21)	396 (31)	464 (16)
	HIV-negative	425 (79)	841 (66)	2 254 (80)
	Unknown	5 (1)	33 (3)	100 (4)
Cough duration	No cough	75 (14)	205 (16)	823 (29)
	Cough 0-13 days	148 (27)	279 (22)	598 (21)
	Cough 14-20 days	90 (17)	140 (11)	321 (11)
	Cough 21-27 days	52 (10)	96 (8)	224 (8)
	Cough >27 days	125 (23)	260 (20)	512 (18)
	Unknown	51 (9)	290 (23)	340 (12)
Cough ≥ 2 weeks	Cough ≥2 weeks present	295 (55)	697 (55)	1 247 (44)
	Cough ≥2 weeks not present	238 (44)	562 (44)	1531 (54)
	Unknown	8 (1)	11 (1)	40 (1)
Fever duration	No fever	146 (27)	352 (28)	1 157 (41)
	Fever 0-13 days	136 (25)	281 (22)	712 (25)
	Fever 14-20 days	67 (12)	122 (10)	262 (9)
	Fever 21-27 days	20 (4)	40 (3)	88 (3)
	Fever >27 days	77 (14)	119 (9)	184 (7)
	Unknown	95 (18)	356 (28)	415 (15)
Fever ≥1 week	Fever ≥1 week present	209 (39)	388 (31)	779 (28)
	Fever ≥1 week not present	242 (45)	650 (51)	1 776 (63)
	Unknown	90 (17)	232 (18)	263 (9)
Lethargy	Lethargy	268 (50)	479 (38)	838 (30)
	No lethargy	226 (42)	565 (44)	1 204 (43)
	Unknown	47 (9)	226 (18)	776 (28)
Weight loss	Weight loss	358 (66)	763 (60)	1 631 (58)
	No weight loss	172 (32)	492 (39)	1 155 (41)
	Unknown	11 (2)	15 (1)	32 (1)
Documented tuberculosis exposure	Documented tuberculosis exposure in previous 12 months	257 (48)	491 (39)	497 (18)
	No documented tuberculosis exposure in previous 12 months	284 (52)	775 (61)	2315 (82)
	Unknown	0 (0)	4 (0)	6 (0)
Night sweats	Night sweats	206 (38)	422 (33)	568 (20)
	No night sweats	187 (35)	577 (45)	1 845 (65)
	Unknown	148 (27)	271 (21)	405 (14)
Haemoptysis	Haemoptysis	2 (0)	13 (1)	12 (0)
	No haemoptysis	88 (16)	410 (32)	851 (30)
	Unknown	451 (83)	847 (67)	1 955 (69)
Objective fever (≥38 degrees Celsius)	Objective fever	71 (13)	101 (8)	156 (6)
	No objective fever	228 (42)	738 (58)	1 476 (52)
	Unknown	242 (45)	431 (34)	1 186 (42)
Tachycardia	Tachycardia	80 (15)	150 (12)	175 (6)
	No tachycardia	179 (33)	611 (48)	1 187 (42)
	Unknown	282 (52)	509 (40)	1 456 (52)
Tachypnoea	Tachypnoea	128 (24)	264 (21)	320 (11)
	No tachypnoea	276 (51)	742 (58)	1 494 (53)
	Unknown	137 (25)	264 (21)	1 004 (36)

Variable	Value	Confirmed tuberculosis (N=541)	Unconfirmed tuberculosis (N=1 270)	Unlikely tuberculosis (N=2 818)
Peripheral lymphadenopathy	Peripheral lymphadenopathy	134 (25)	242 (19)	280 (10)
	No peripheral lymphadenopathy	295 (55)	759 (60)	1 444 (51)
	Unknown	112 (21)	269 (21)	1 094 (39)
First Xpert MTB/RIF	Xpert positive for Mtb	247 (46)	0 (0)	0 (0)
	Xpert negative for Mtb	273 (50)	1148 (90)	2 306 (82)
	Unknown/not performed	21 (4)	122 (10)	512 (18)
Overall CXR read	CXR consistent with tuberculosis	344 (64)	644 (51)	653 (23)
	CXR not consistent with tuberculosis	101 (19)	474 (37)	1 744 (62)
	Unknown/not assessed	96 (18)	152 (12)	421 (15)
Opacities on CXR	Opacities present on CXR	237 (44)	487 (38)	594 (21)
	Opacities not present on CXR	154 (28)	459 (36)	1 042 (37)
	Unknown/not assessed	150 (28)	324 (26)	1 182 (42)
Cavities on CXR	Cavities present on CXR	32 (6)	25 (2)	20 (1)
	Cavities not present on CXR	348 (64)	793 (62)	973 (35)
	Unknown/not assessed	161 (30)	452 (36)	1 825 (65)
Miliary infiltrate on CXR	Miliary infiltrate present on CXR	33 (6)	19 (1)	7 (0)
	Miliary infiltrate not present on CXR	358 (66)	927 (73)	1 614 (57)
	Unknown/not assessed	150 (28)	324 (26)	1 197 (42)
Intrathoracic lymphadenopathy on CXR	Intrathoracic lymphadenopathy present on CXR	154 (28)	248 (20)	104 (4)
	Intrathoracic lymphadenopathy not present on CXR	237 (44)	684 (54)	1 485 (53)
	Unknown/not assessed	150 (28)	338 (27)	1 229 (44)
Pleural effusion on CXR	Pleural effusion present on CXR	56 (10)	63 (5)	49 (2)
	Pleural effusion not present on CXR	335 (62)	883 (70)	1 570 (56)
	Unknown/not assessed	150 (28)	324 (26)	1 199 (43)
Tuberculin skin test	TST positive	298 (55)	464 (37)	262 (9)
	TST negative	174 (32)	674 (53)	1 972 (70)
	Unknown/not performed	69 (13)	132 (10)	584 (21)

Appendix M: Study quality assessment

Table S42. Modified Newcastle-Ottawa Scale for cohort studies[21]

**Selection**	**Kabir/2020/BD**	**Aurilio/2020/BR**	**Song/2021/KE**	**LopezVarela/2015/MZ**	**Valencia/2017/MZ**	**Myo/2018/MM**	**Marcy/2019/Multi**	**Hamid/2019/PK**	**Zar/2019/ZA**	**Walters/2017/ZA**	**Orikiriza/2018/UG**	**Orikiriza/2022/UG**	**Giang/2015/VN**
1) Representativeness of the exposed cohort													
a) truly representative of the target population in the community*													
b) somewhat representative of the target population in the community*	✓	✓		✓	✓	✓	✓	✓	✓	✓	✓		✓
c) selected group of users			✓									✓	
d) no description of the derivation of the cohort													
2) Selection of the non-exposed cohort													
a) drawn from the same community as the exposed cohort*	✓	✓	✓	✓	✓	✓	✓	✓	✓	✓	✓	✓	✓
b) drawn from a different source													
c) no description of the derivation of the non-exposed cohort													
3) Ascertainment of TB reference classification													
a) secure record*													
b) structured interview*	✓	✓	✓	✓	✓	✓	✓	✓	✓	✓	✓	✓	✓
c) written self-report													
d) no description													
**Comparability**													
1) Comparability of cohorts on the basis of the design or analysis	N/A	N/A	N/A	N/A	N/A	N/A	N/A	N/A	N/A	N/A	N/A	N/A	N/A
**Outcome**													
1) Assessment of reference classification													
a) independent assessment blinded to TB reference classification*	✓	✓	✓			✓	✓		✓	✓	✓	✓	
b) record linkage*				✓	✓			✓					✓
c) self-report													
d) no description													
2) Follow-up of cohorts to establish reference classification													
a) Follow-up for all participants*		✓	✓			✓	✓	✓	✓	✓	✓	✓	✓
b) Follow-up for only participants treated for TB	✓			✓	✓								
c) follow-up information not used to establish reference classification													
3) Adequacy of follow-up of cohorts	N/A	N/A	N/A	N/A	N/A	N/A	N/A	N/A	N/A	N/A	N/A	N/A	N/A
**Total** (out of maximum score 5)	**4**	**5**	**4**	**4**	**4**	**5**	**5**	**5**	**5**	**5**	**5**	**4**	**5**

Appendix N: Existing algorithms and modifications to make maximal use of IPD

Table S43. Modifications to Marais et al. criteria[22]

Algorithm	Variable in data	Differences
Persistent, nonremitting cough > 2 weeks	Cough duration	Cannot specify cough characteristic (persistent and nonremitting)
Objective weight loss (documented failure to thrive) during the preceding 3 months	Weight loss	Definition of weight loss was not specific to failure to thrive
Reported fatigue	Lethargy	

Figure S3. The Union's desk guide[23]

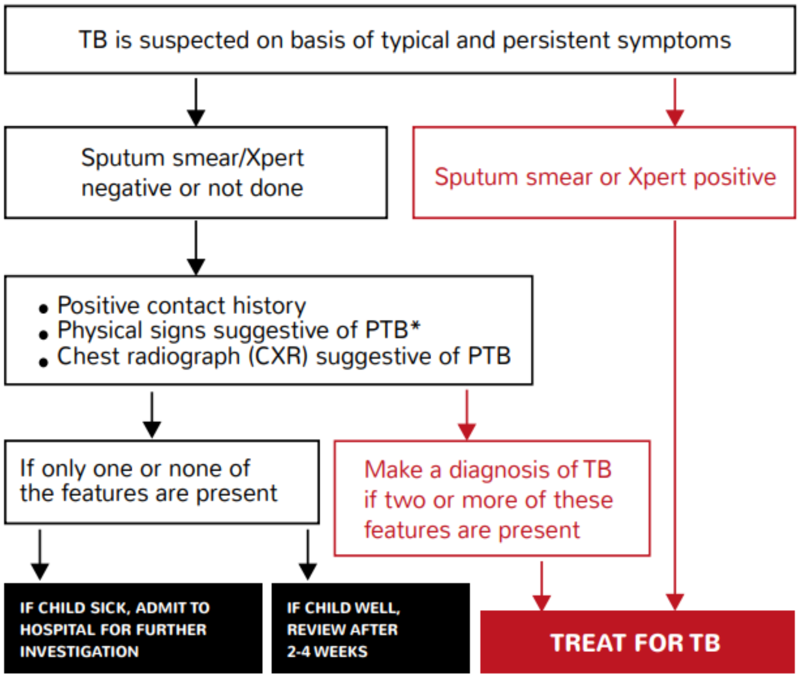

Table S44. Modifications to The Union's desk guide

Algorithm	Variables in data	Differences
**Strict Symptom Criteria**		
Persistent, non-remitting cough or wheeze for more than 2 weeks not responding to standard therapy	Cough duration	Cannot specify cough characteristic (persistent and nonremitting)
Documented loss of weight or failure to thrive during past 3 months especially if not responding to food and/or micronutrient supplementation, or severe malnutrition	Weight loss<br><br>Weight/Height/Age	Definition of weight loss was not specific to failure to thrive<br><br>Use weight/height/age to determine if severely acutely malnourished
Fatigue/reduced playfulness	Lethargy	
Persistent fever >10 days	Fever duration	Evaluated as fever >7 days
**TB contact in the preceding year**	Documented TB exposure	Some studies defined documented TB exposure as within the previous 24 months
**HIV**	HIV status	
**Physical signs**		
Weight loss or poor weight gain, evidence of growth faltering	Weight loss	Definition of weight loss was not specific to failure to thrive
Fever	Temperature (C)	
Increased respiratory rate	Respiratory rate (per min)	
Signs of respiratory distress	N/A	N/A
Auscultation and percussion	N/A	N/A
**CXR**		
Enlarged hilar lymph nodes	Intrathoracic lymphadenopathy on CXR	
Opacification in lung tissue	Opacities on CXR	
Miliary mottling	Miliary infiltrate on CXR	
Cavitation	Cavities on CXR	
Pleural or pericardial effusion	Pleural effusion on CXR	Did not evaluate pericardial effusion
Marked abnormality on CXR in child with no signs of respiratory distress (no fast breathing or chest indrawing) is supportive of TB	N/A	N/A
**Sputum Xpert**	First Xpert MTB/RIF	
**Sputum smear**	N/A	N/A

Figure S4. Stegen-Toledo Score[24]

Finding	Score
Positive culture result	7
Tuberculous granuloma	4
Positive PPD test result[a]	3
Known contact with a person with TB during past 2 years	2
Radiographic results suggestive of TB	2
Clinical presentation suggestive of TB[b]	2

NOTE. Highly probable tuberculosis was denoted by a Stegen-Toledo score of ≥7; probable tuberculosis, by a score of 5–6; suspected tuberculosis, by a score of 3–4; and unlikely tuberculosis, by a score of 0–2. PPD, tuberculin purified protein derivative.

[a] Induration >10 mm.

[b] Duration of cough >2 weeks.

Table S45. Modifications to Stegen-Toledo Score (using cutoff of 5 points to classify TB)

Algorithm	Variables in data	Differences
Positive culture result	First Xpert MTB/RIF	Used Xpert rather than culture given practical advantage of Xpert
Tuberculosis granuloma	N/A	N/A
Positive PPD test result	TST result	
Known contact with a person with TB during past 2 years	Documented TB exposure	Some studies defined documented TB exposure as within the previous 12 months
Radiological results suggestive of TB	CXR consistent with TB	
Clinical presentation suggestive of TB (defined as duration of cough >2 weeks)	Cough duration	

Figure S5. Uganda National Tuberculosis and Leprosy Control Programme (NTLP) algorithm[25]

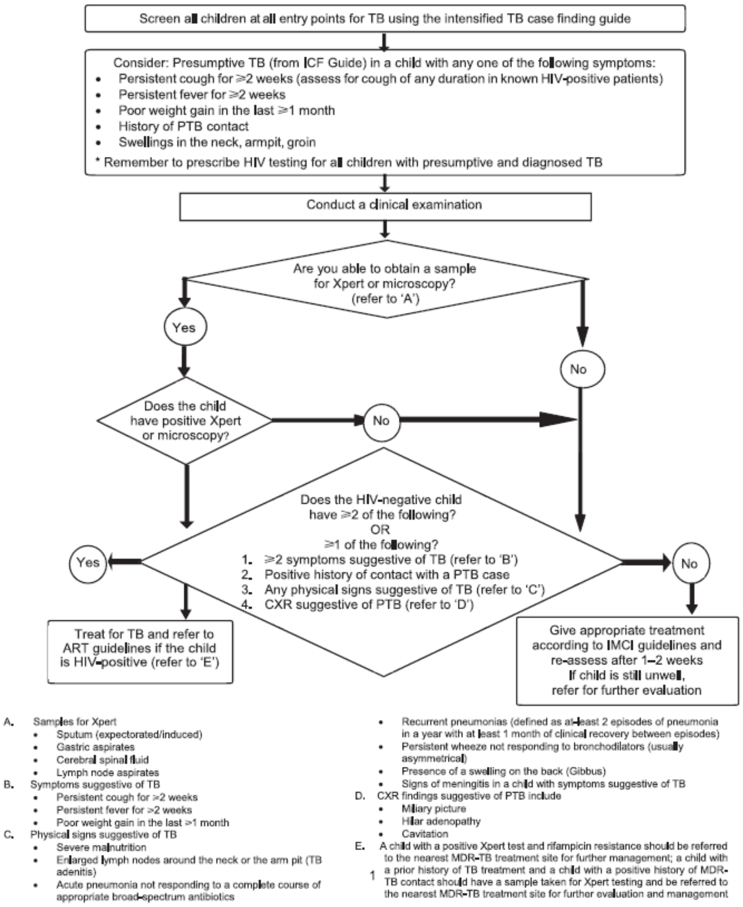

Table S46. Modifications to Uganda NTLP Algorithm

Algorithm	Variables in data	Differences
**Xpert or microscopy**	First Xpert MTB/RIF	Did not evaluate microscopy
**Symptoms suggestive of TB** (≥2 of the following)		
Persistent cough ≥2 wks	Cough duration	
Persistent fever for ≥2 wks	Fever duration	
Poor weight gain in the last ≥1 month	Weight loss	
**CXR findings suggestive of PTB**		
Miliary picture	Miliary infiltrate on CXR	
Hilar adenopathy	Intrathoracic lymphadenopathy on CXR	
Cavitation	Cavities on CXR	
**Physical signs suggestive of TB**		
Severe malnutrition	Weight/Height/Age	Use weight/height/age to determine if severely acutely malnourished
Enlarged lymph nodes around neck or arm pit	Peripheral lymphadenopathy	
Acute pneumonia not responding to complete course of appropriate antibiotics	N/A	N/A
Recurrent pneumonias	N/A	N/A
Persistent wheeze not responding to bronchodilators	N/A	N/A
Persistence of swelling on the back (Gibbus)	N/A	N/A
Signs of meningitis in child with symptoms suggestive of TB	N/A	N/A

Figure S6. Brazilian Ministry of Health Score[26]

Diagnosis of pulmonary tuberculosis in children and adolescents with negative smear microscopy or undetected RMT 2 .

Clinical condition	**Radiological patterns**	Contact with adult with TB*	TST*	**Nutritional status**
Fever or symptoms such as cough, adynamia, expectoration, slimming, sweating > 2 weeks **15 points**	Hilar adenomegaly or miliary pattern and/or condensation or infiltrate (with or without cavitation) unchanged by ≥ 2 weeks and/or condensation or infiltrate (with or without excavation) for ≥ 2 weeks, progressing with worsening or without improvement with antibiotics for common germs **15 points**	Close contact in he last 2 years **10 points**	TST between 5 and 9 mm **5 points** TST ≥10 mm **10 points**	Serious malnutrition **5 points**
Asymptomatic or with symptoms < 2 weeks **0 points**	Condensation or infiltrate of any type for less than 2 weeks **5 points**	Occasional or negative **0 points**	TST < 5 mm **0 points**	Weight ≥ 10 th percentile **0 points**
Respiratory infection that improved after using antibiotics for common germs or without antibiotics (**– 10 points)**	Normal radiography **(–5 points)**			

RMT = rapid molecular test; TB = tuberculosis; TST = tuberculin skin test. At least 40 points (very likely diagnosis) = it is recommended to start TB treatment; 30- 35 points (possible diagnosis) = indicative of TB; it is advised to initiate treatment, at medical discretion; Less than 25 points (diagnosis is unlikely) = investigation of the child should be continued.

Table S47. Modifications to Brazilian Ministry of Health Score (using cutoff of 30 points to classify TB)

Algorithm	Variables in data	Differences
Fever ≥2 *wks*	Fever duration	
Cough ≥2 *wks*	Cough duration	
Adynamia ≥2 *wks*	Lethargy	Duration not specified in data
Expectoration ≥2 *wks*	N/A	N/A
Slimming ≥2 *wks*	Weight loss	Duration not specified in data
Sweating ≥2 *wks*	Night sweats	Duration not specified in data
Respiratory infection that improved after using antibiotics for common germs or without antibiotics	N/A	
Hilar adenomegaly or miliary pattern and/or condensation or infiltrate (with or without cavitation) unchanged by ≥ 2 weeks and/or condensation or infiltrate (with or without excavation) for ≥ 2 weeks, progressing with worsening or without improvement with antibiotics for common germs	Intrathoracic lymphadenopathy on CXR<br>Miliary infiltrate on CXR<br>Opacities on CXR<br>Cavities on CXR	CXR abnormalities consistent with TB of unknown duration
Condensation or infiltrate of any type for less than 2 weeks	N/A	No data on duration of CXR abnormalities consistent with TB
Normal radiography	N/A	No data to indicate CXR
Close contact in the last 2 years (with adult with TB)	Documented TB exposure	Some studies defined documented TB exposure as within the previous 12 months
TST diameter	TST result	TST diameter not specified in data, only whether result was positive or not
Serious malnutrition (weight $<10^{th}$ percentile)	Weight/age	Weight and age used to compute weight-for-age z-score

Figure S7. Keith-Edwards Score[27]

Table 1. Keith Edwards Score for Diagnosis of Tuberculosis in Children

Feature	Score		
	0	1	3
Duration of illness (weeks)	< 2	2-4	> 4
Nutrition (% of weight for age)	> 80	60-80	< 60
Family history of tuberculosis	None	Reported by family	Proven sputum positive

Score for Other Features if Present

Feature	Score
Unexplained fever, night sweats, no response to malaria treatment	2
Positive tuberculin test	3
Lymph nodes: large, painless, firm, soft sinus in neck/axilla	3
Malnutrition, not improving after 4 weeks	3
Central nervous system : change in temperament, fits with or without abnormal cerebrospinal fluid findings	3
Joint swelling, bone swelling, sinuses	3
Unexplained abdominal mass, ascites	3
Angle deformity of spine	4

A score of 7 or more is indicative of tuberculosis

Table S48. Modifications to Keith-Edwards Score

Algorithm	Variables in Data	Differences
Duration of illness	Cough duration<br>Fever duration	Used the max of cough or fever duration to represent duration of illness
Nutrition (% of weight for age)	Weight/age	Weight and age used to compute weight-for-age z-score
Family history of tuberculosis	Documented TB exposure	Data unavailable on whether TB exposure was bacteriologically-confirmed
Fever	Temperature (C)	
Night sweats	Night sweats	
No response to malaria treatment	N/A	N/A
Lymph nodes: large, painless, firm, soft sinus in neck/axilla	Peripheral lymphadenopathy	
Malnutrition, not improving after 4 weeks	Weight loss	Cannot specify whether malnutrition did not improve after 4 weeks
Central nervous system: change in temperament, fits with or without abnormal cerebrospinal fluid findings	Lethargy	Unable to evaluate fits or abnormal cerebrospinal fluid findings
Joint swelling, bone swelling, sinuses	N/A	N/A
Unexplained abdominal mass, ascites	N/A	N/A
Angle deformity of spine	N/A	N/A

Figure S8. Gunasekera et al. algorithm[28]

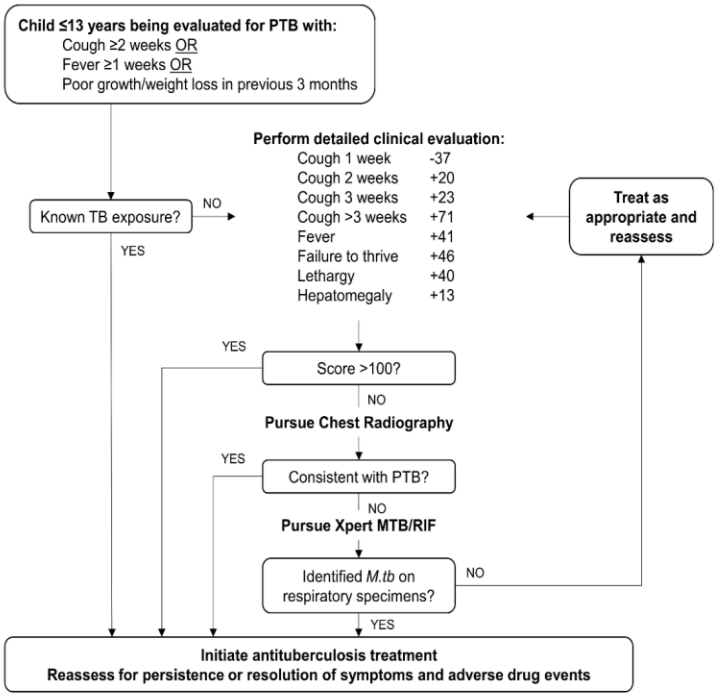

Table S49. Modifications to Gunasekera et al. Algorithm. Given that we had access to the Walters/2017/ZA data from HIV-negative children used to develop this algorithm, we refit the logistic regression model using a complete case analysis of variables available in the IPD before any imputation using the composite TB reference standard; thus, this algorithm is modified from the originally stated algorithm (we do not include hepatomegaly and fever is defined as ≥1 week). This model had an AUC of 0·85. The model parameter coefficients were scaled to produce a score such that a sum of the scores resulted in classification of TB with a sensitivity of 90% -- this resulted in an algorithm with a sensitivity of 91% and a specificity of 49%.

	OR	2·50%	97·5%	p-value	Scaled score
**No cough**	--	--	--	--	--
**Cough < 2 weeks**	0·81	0·37	1·78	0·60	-22
**Cough 2 weeks**	1·14	0·40	3·18	0·81	13
**Cough 3 weeks**	1·56	0·46	5·25	0·47	46
**Cough >3 weeks**	3·60	1·49	9·01	0·01	132
**No fever or fever <1 week**	--	--	--	--	--
**Fever ≥1 week**	2·12	0·87	5·33	0·10	78
**No weight loss**	--	--	--	--	--
**Weight loss**	1·98	1·06	3·76	0·03	71
**No lethargy**	--	--	--	--	--
**Lethargy**	1·43	0·71	2·88	0·32	37
**No history of documented TB contact**	--	--	--	--	--
**History of documented TB contact**	6·64	3·53	12·99	0·00	195
**CXR not consistent with TB**	--	--	--	--	--
**CXR consistent with TB**	11·02	5·39	23·90	0·00	248
**Xpert negative for Mtb**	--	--	--	--	--
**Xpert positive for Mtb**	13927274·15	0·00	Inf	0·98	1698

Figure S9. Marcy et al. Algorithm[14]

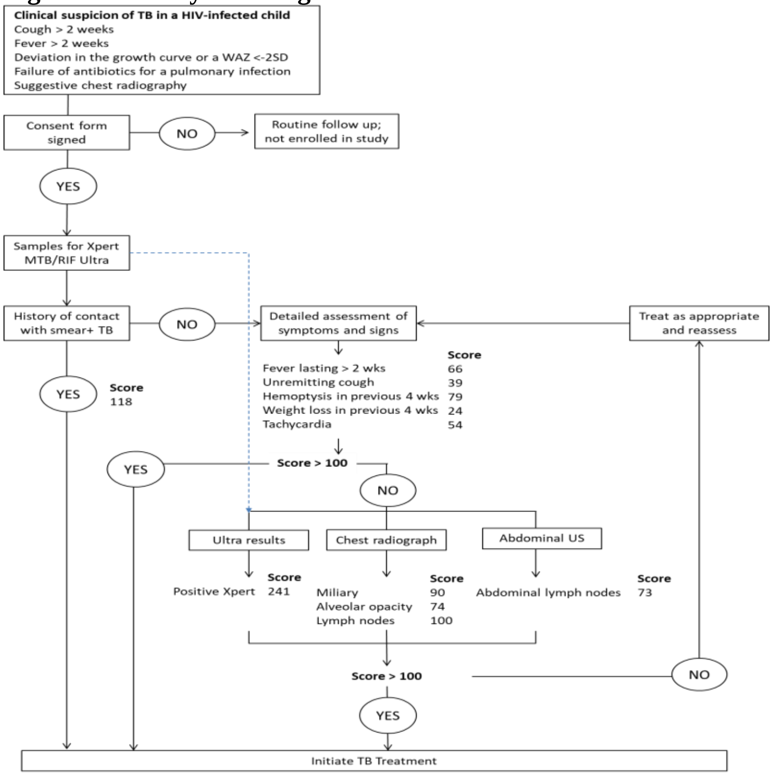

Table S50. Modifications to Marcy et al. Algorithm. Given that we had access to the Marcy/2019/Multi data from HIV-positive children used to develop this algorithm, we refit the logistic regression model using a complete case analysis of variables available in the IPD before any imputation using the composite TB reference standard; thus, this algorithm is modified from the originally stated algorithm (we use cough ≥2 weeks rather than remitting cough, and we do not include abdominal ultrasound results). This model had an AUC of 0·80. The model parameter coefficients were scaled to produce a score such that a sum of the scores resulted in classification of TB with a sensitivity of 90% -- this resulted in an algorithm with a sensitivity of 91% and a specificity of 40%.

	odds-ratio	2·50%	97·5%	p-value	Scaled score
No cough or cough <2 weeks	--	--	--	--	--
Cough ≥2 weeks	1·11	0·52	2·37	0·78	9
No fever or fever <1 week	--	--	--	--	--
Fever ≥1 week	2·94	1·72	5·39	0·00	95
No weight loss	--	--	--	--	--
Weight loss	1·79	1·01	3·37	0·05	52
No haemoptysis	--	--	--	--	--
Haemoptysis	3·29	0·62	93·23	0·23	105
No tachycardia	--	--	--	--	--
Tachycardia	2·03	0·91	5·12	0·09	62
No history of documented TB contact	--	--	--	--	--
History of documented TB contact	1·71	0·60	5·59	0·33	47
Miliary infiltrate not present on CXR	--	--	--	--	--
Miliary infiltrate present on CXR	2·56	0·77	10·36	0·14	83
Opacities not present on CXR	--	--	--	--	--
Opacities present on CXR	2·36	1·32	4·53	0·00	76
Intrathoracic lymphadenopathy not present on CXR	--	--	--	--	--
Intrathoracic lymphadenopathy present on CXR	5·41	2·84	11·83	0·00	149
Xpert negative for Mtb	--	--	--	--	--
Xpert positive for Mtb	29·18	3·40	Inf	0·03	298

Appendix O: Performance of existing algorithms against reference classification of all TB

Figure S10. Performance of Marais et al. criteria. Study-level and pooled estimates of the **(A)** sensitivity and **(B)** specificity of classifying TB (composite reference standard: bacteriologically-confirmed pulmonary TB and unconfirmed pulmonary TB).

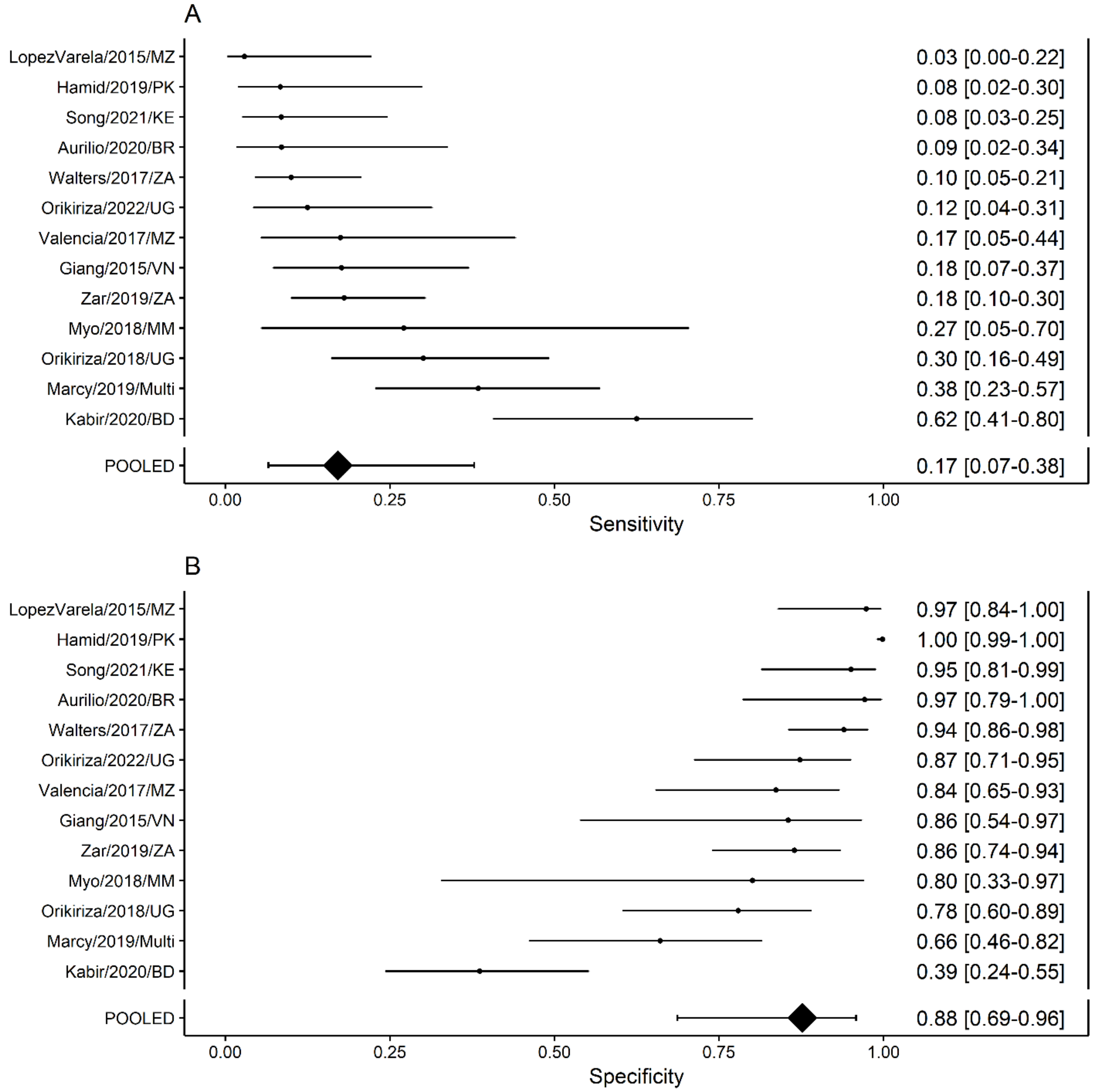

Figure S11. Performance of Stegen-Toledo Score (using cutoff of 5 points to classify TB). Study-level and pooled estimates of the (A) sensitivity and (B) specificity of classifying TB (composite reference standard: bacteriologically-confirmed pulmonary TB and unconfirmed pulmonary TB).

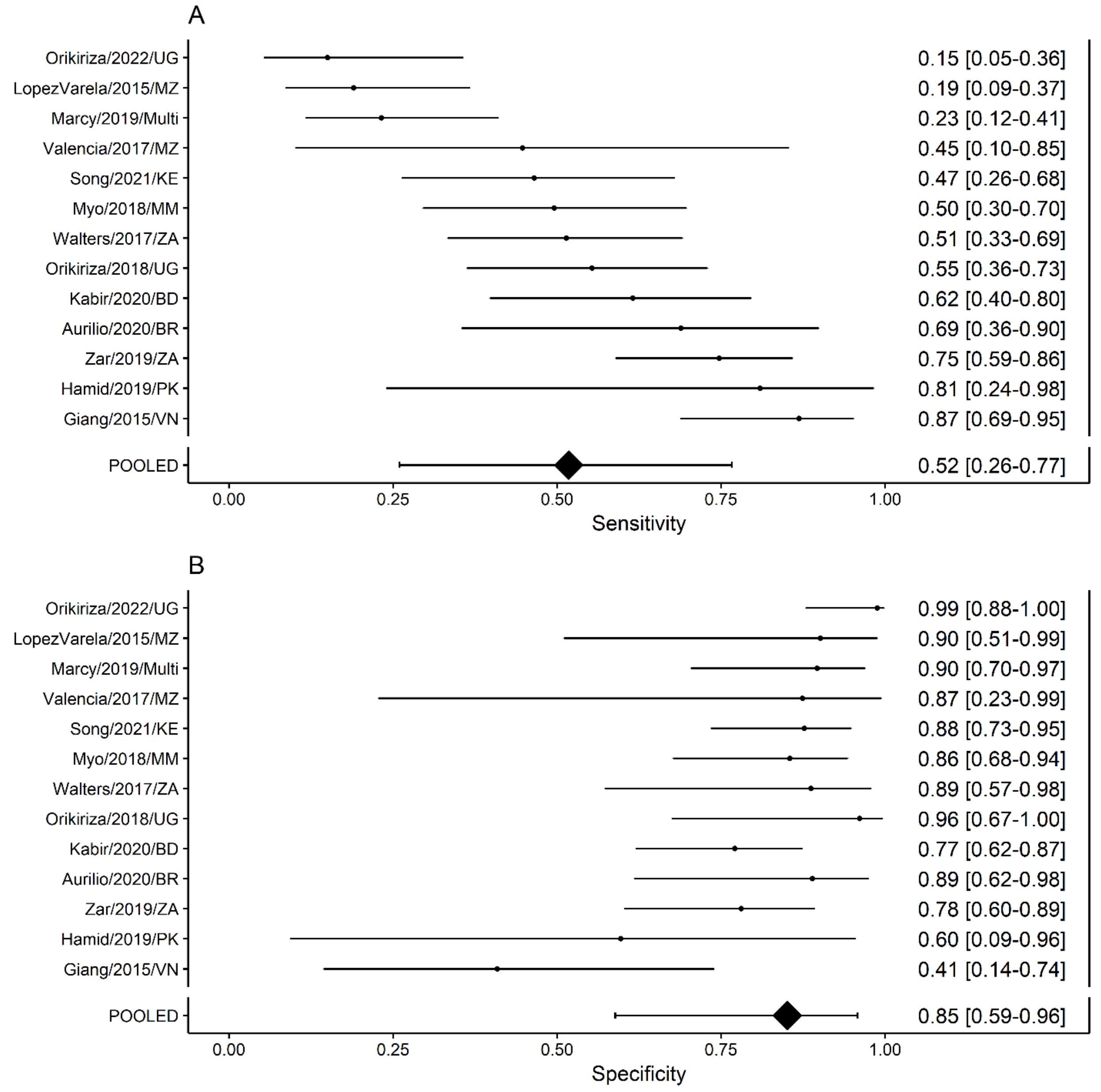

Figure S12. Performance of Uganda NTLP Algorithm. Study-level and pooled estimates of the **(A)** sensitivity and **(B)** specificity of classifying TB (composite reference standard: bacteriologically-confirmed pulmonary TB and unconfirmed pulmonary TB).

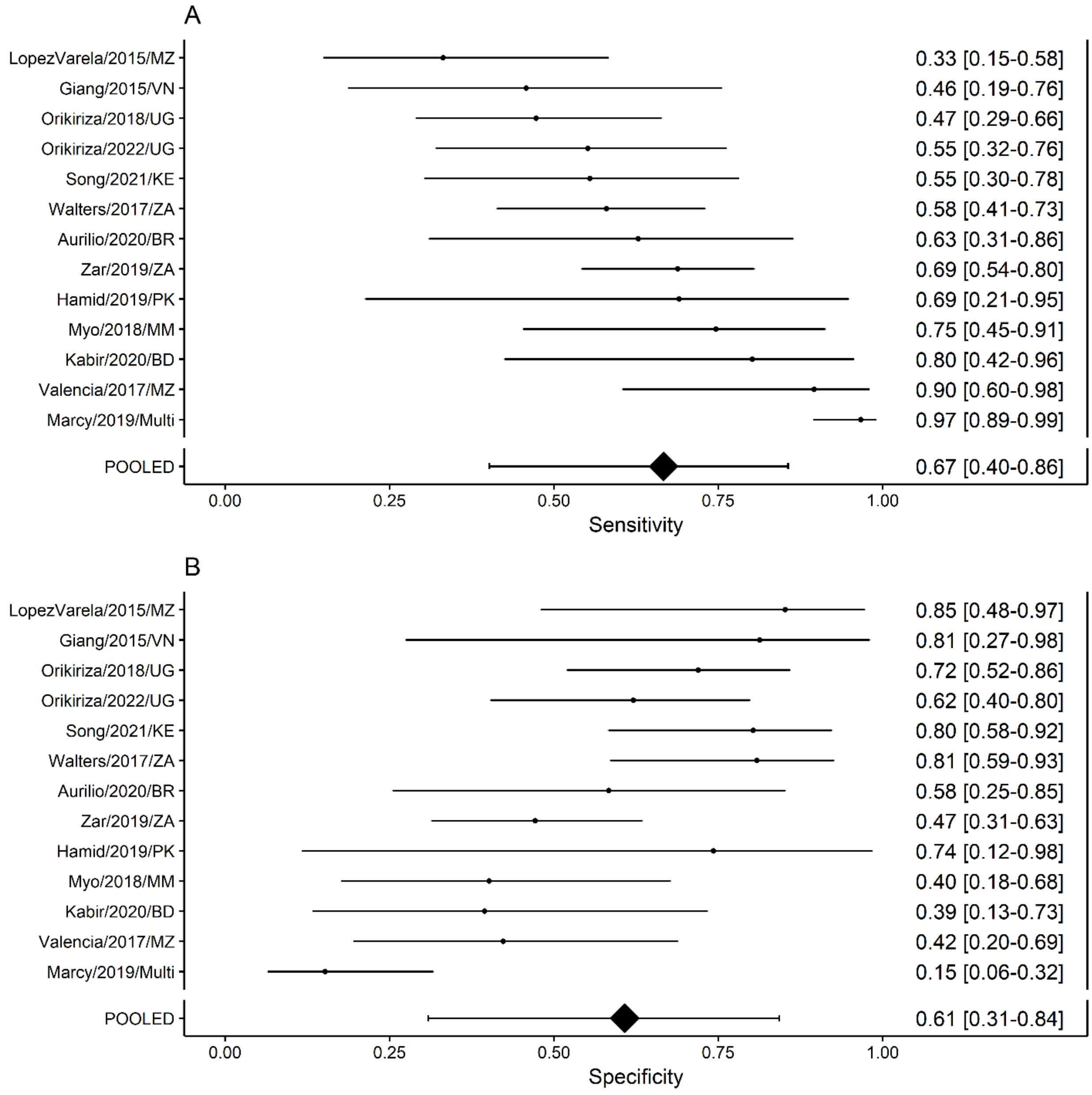

Figure S13. Performance of The Union's Desk Guide. Study-level and pooled estimates of the **(A)** sensitivity and **(B)** specificity of classifying TB (composite reference standard: bacteriologically-confirmed pulmonary TB and unconfirmed pulmonary TB).

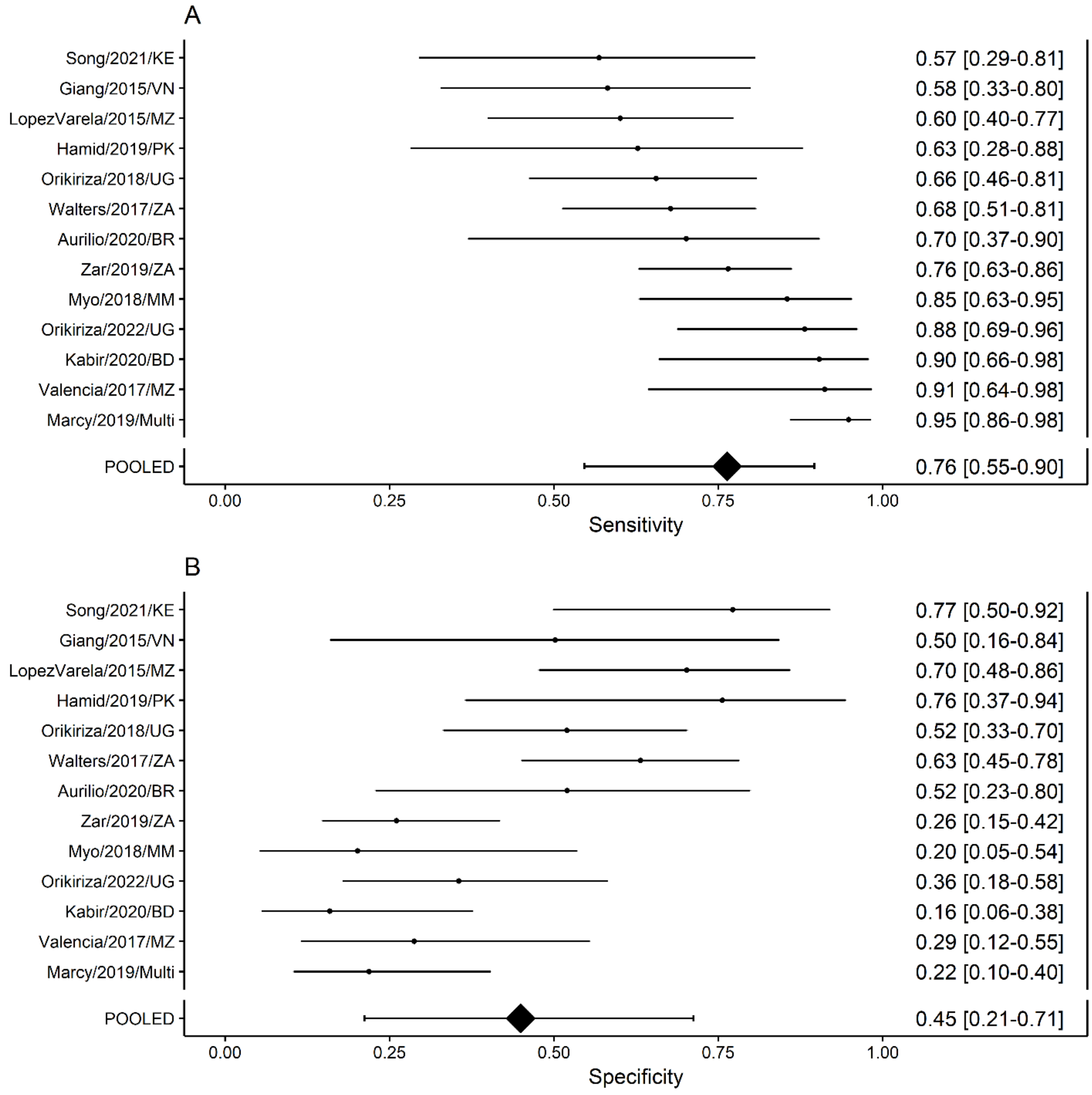

Figure S14. Performance of Brazilian Ministry of Health Score (using cutoff of 30 points to classify TB) Study-level and pooled estimates of the **(A)** sensitivity and **(B)** specificity of classifying TB (composite reference standard: bacteriologically-confirmed pulmonary TB and unconfirmed pulmonary TB).

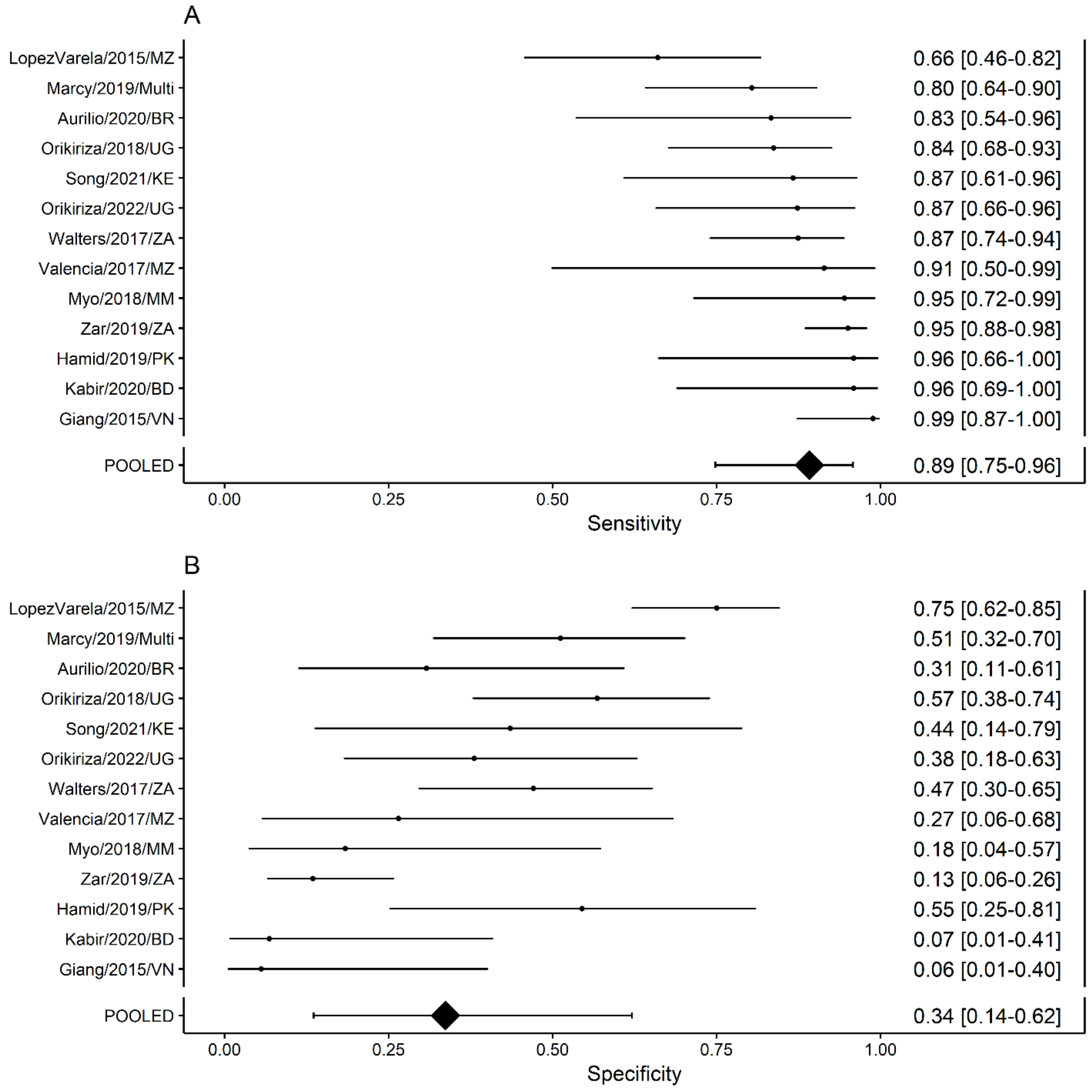

Figure S15. Performance of Keith-Edwards Score. Study-level and pooled estimates of the **(A)** sensitivity and **(B)** specificity of classifying TB (composite reference standard: bacteriologically-confirmed pulmonary TB and unconfirmed pulmonary TB).

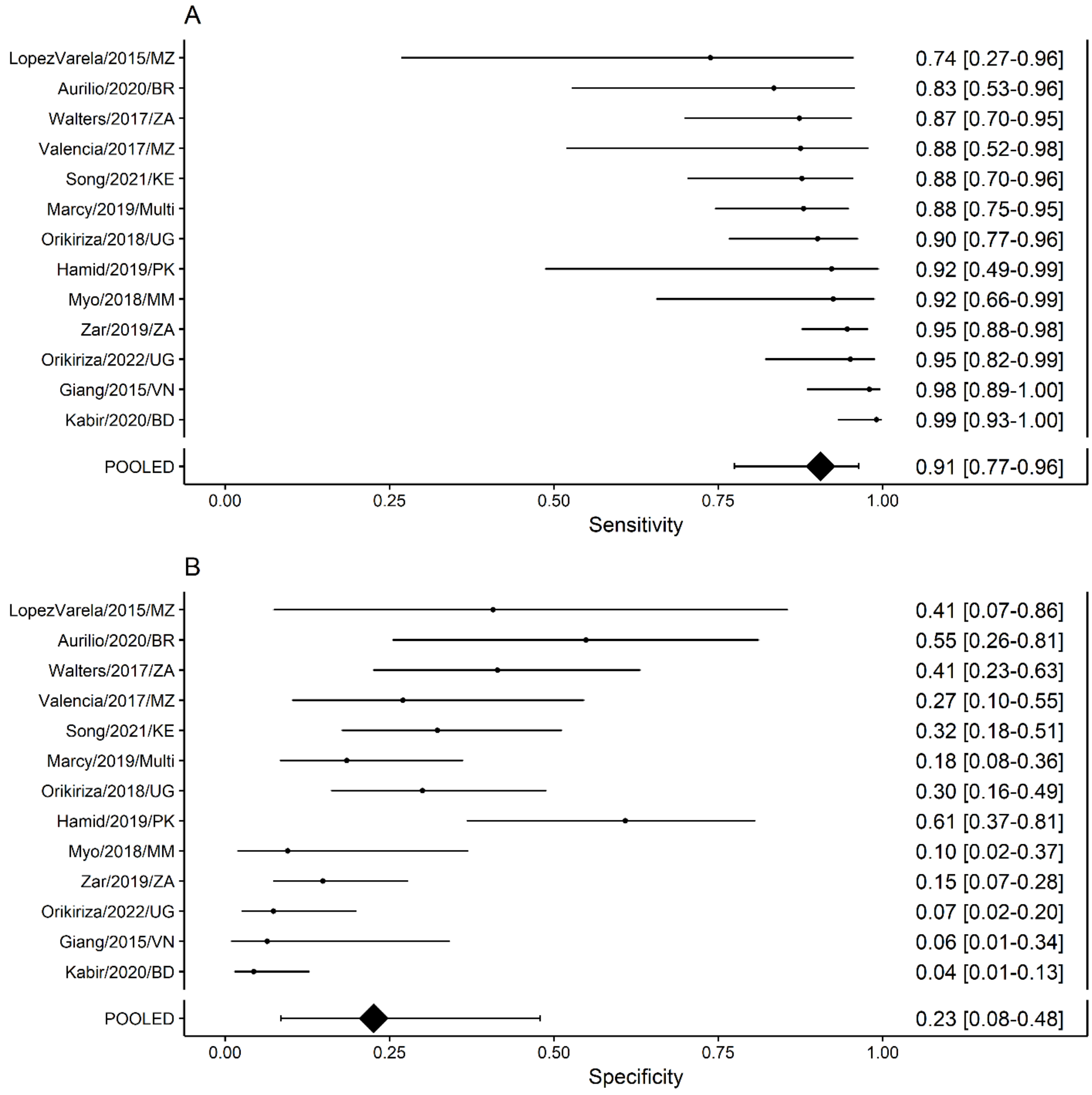

Figure S16. Performance of Marcy et al. Algorithm. Performance estimates of the Marcy et al. Algorithm were derived from only HIV-positive children in the IPD that excludes data form the Marcy/2019/Multi cohort (from which the algorithm was developed). Study-level and pooled estimates of the **(A)** sensitivity and **(B)** specificity of classifying TB (composite reference standard: bacteriologically-confirmed pulmonary TB and unconfirmed pulmonary TB).

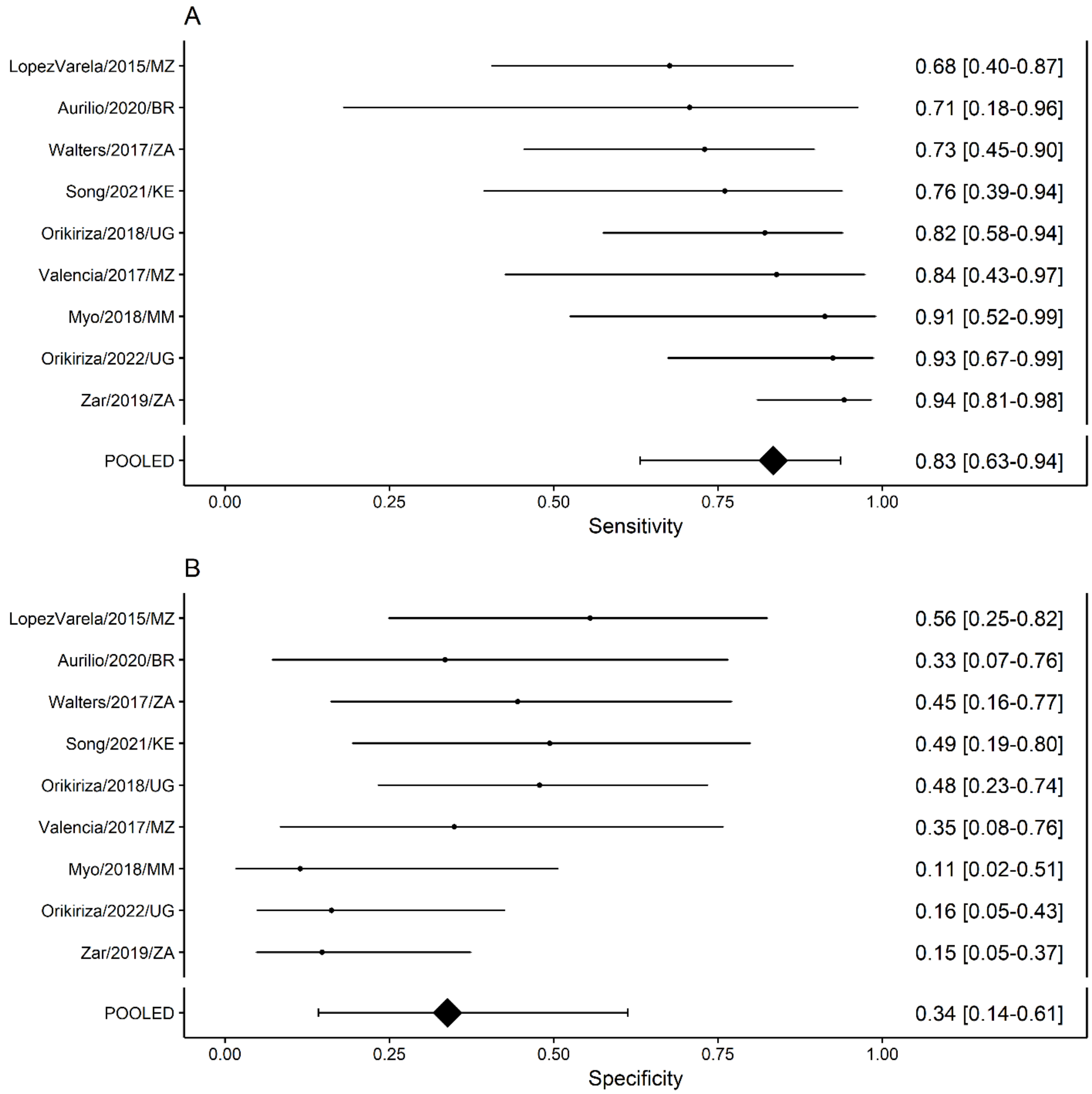

Figure S17. Performance of Gunasekera et al. Algorithm. Performance estimates of the Gunasekera et al. Algorithm were derived from only HIV-negative children in the IPD that excludes data from the Walter/2017/ZA population (from which the algorithm was developed). Study-level and pooled estimates of the **(A)** sensitivity and **(B)** specificity of classifying TB (composite reference standard: bacteriologically-confirmed pulmonary TB and unconfirmed pulmonary TB).

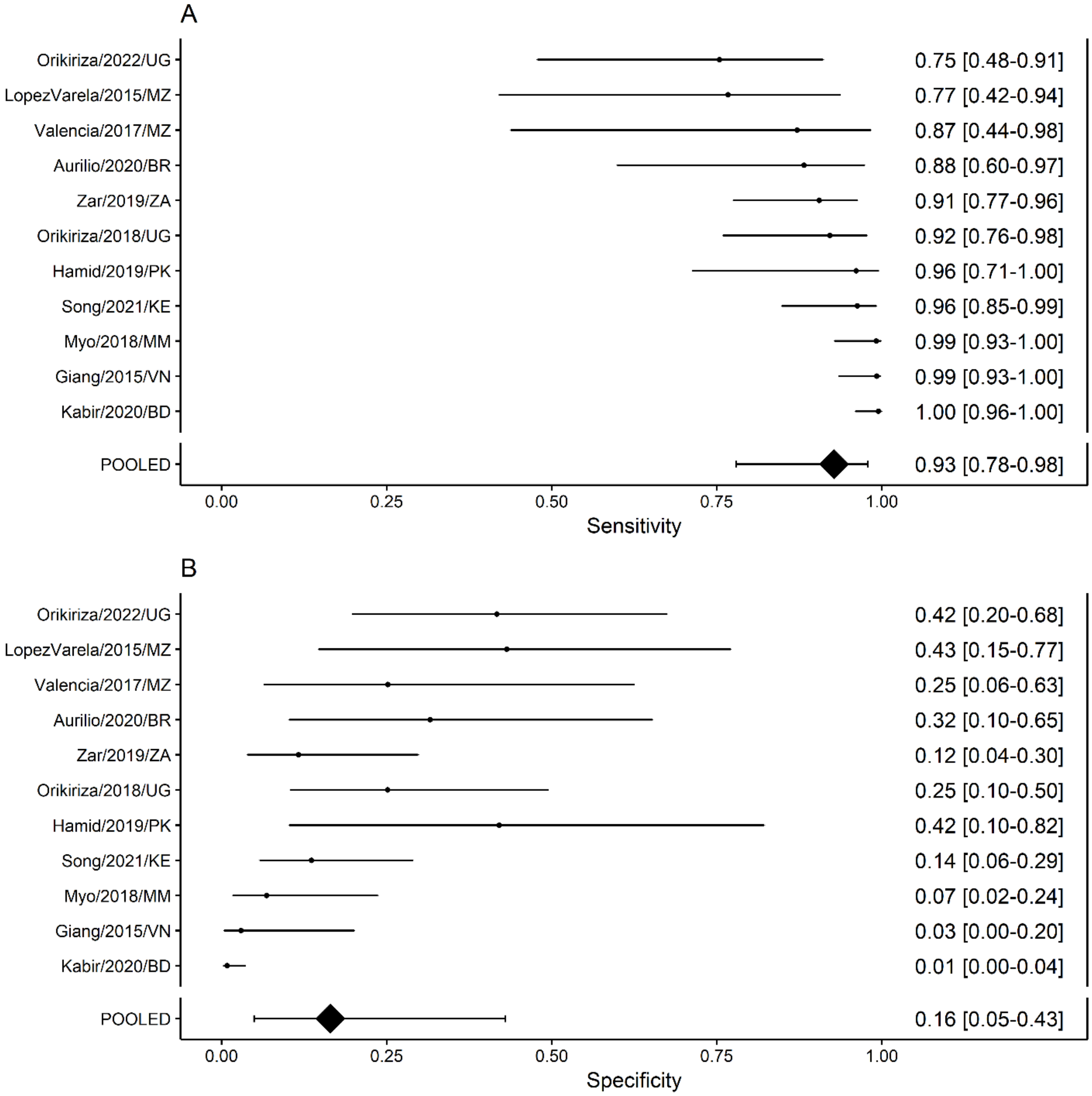

Appendix P: Performance of existing algorithms against reference classification of bacteriologically-confirmed TB

Figure S18. Performance of existing algorithms at classifying confirmed TB (excluding data from children with unconfirmed TB). Retrospective estimates of the pooled **(A)** sensitivity and **(B)** specificity of eight algorithms to guide decisions to treat children with presumptive pulmonary TB, had they been used to evaluate the children for whom we have IPD records. The reference classification of pulmonary TB included bacteriologically-confirmed pulmonary TB only (children with unconfirmed TB are excluded from this analysis).

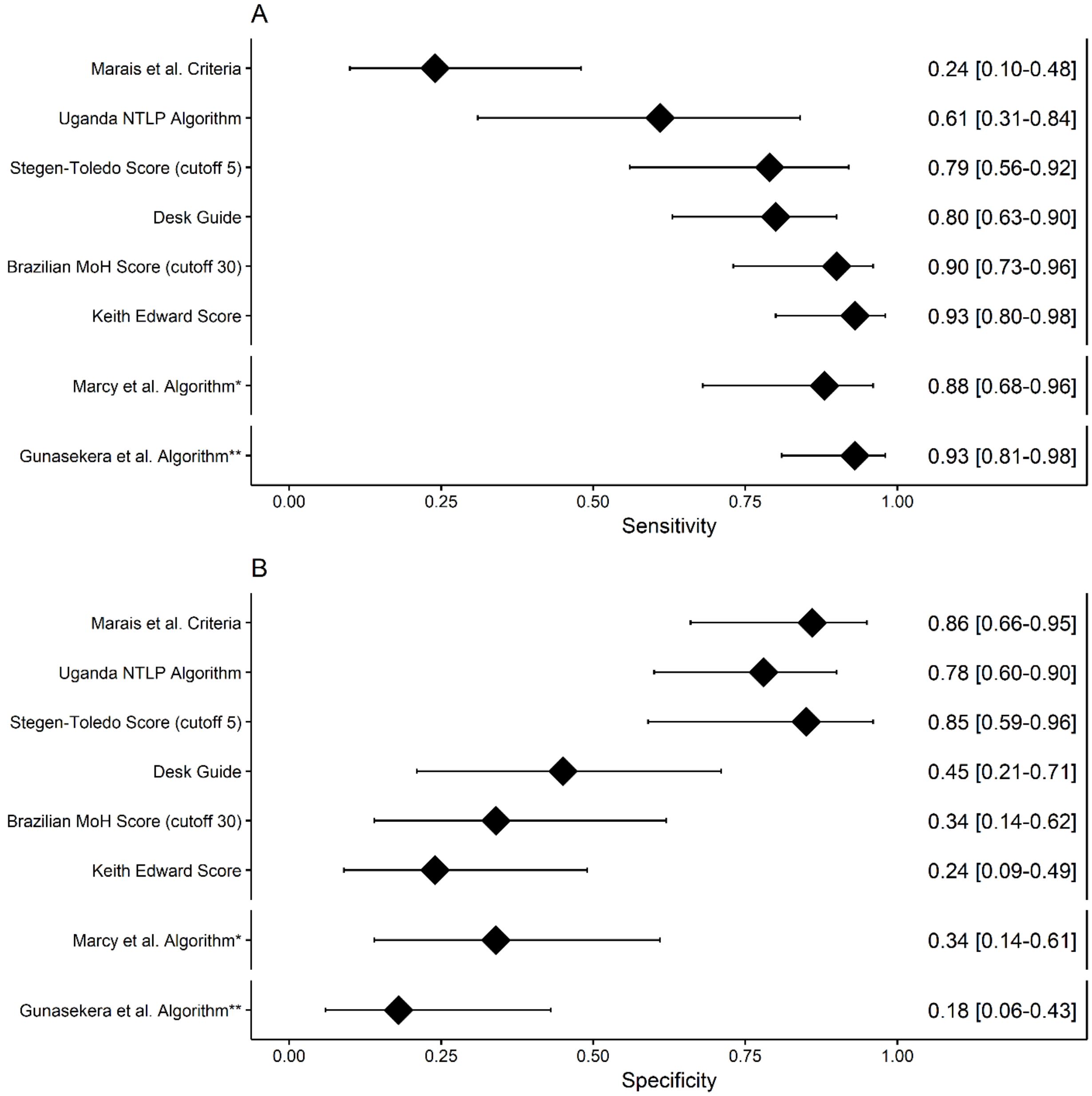

Appendix Q: Discrimination and calibration of prediction model to classify TB

Figures S19. Forest plot depicting discrimination and calibration of prediction model including chest x-ray features to classify TB. Study-level and pooled estimates of the **(A)** discrimination (c-statistic) and calibration [(**B**) calibration intercept and (**C**) O:E ratio] of the prediction model developed from the IPD in classifying TB using an internal-external cross-validation framework (reference standard: bacteriologically-confirmed pulmonary TB and unconfirmed pulmonary TB). c-Statistic – concordance statistic, O:E – observed: expected ratio, IPD – individual participant data, TB – tuberculosis, BD – Bangladesh, BR – Brazil, KE – Kenya, MM – Myanmar, Multi – Multi-country study (includes Burkina Faso, Cameroon, Vietnam, and Cambodia), MZ – Mozambique, PK – Pakistan, UG – Uganda, VN – Vietnam, ZA – South Africa.

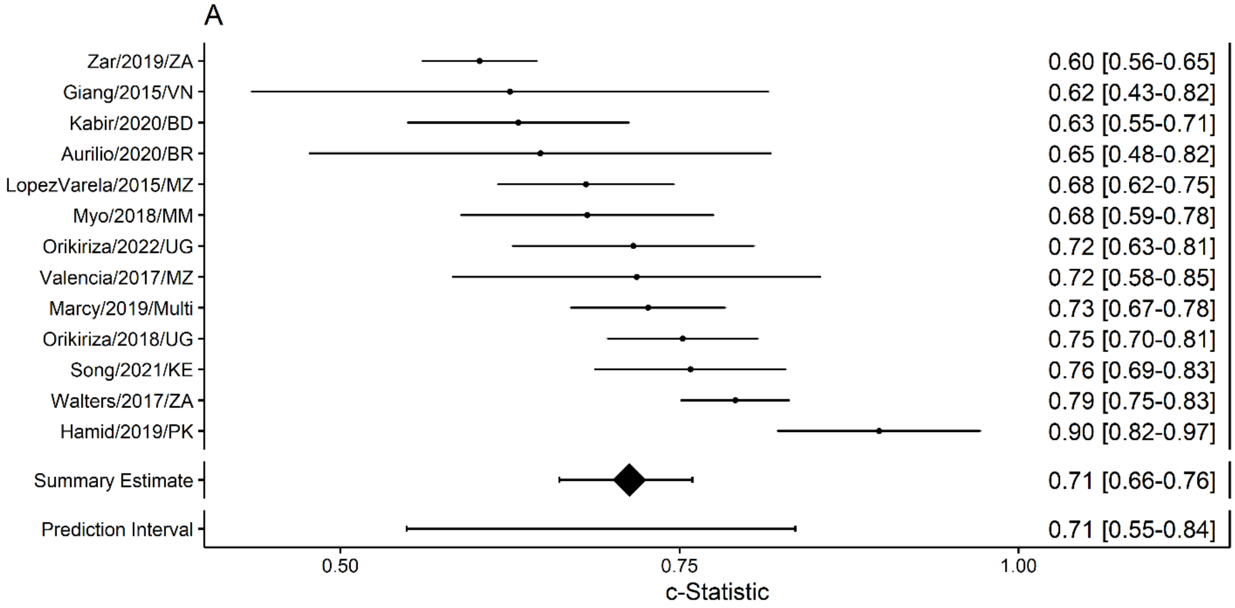

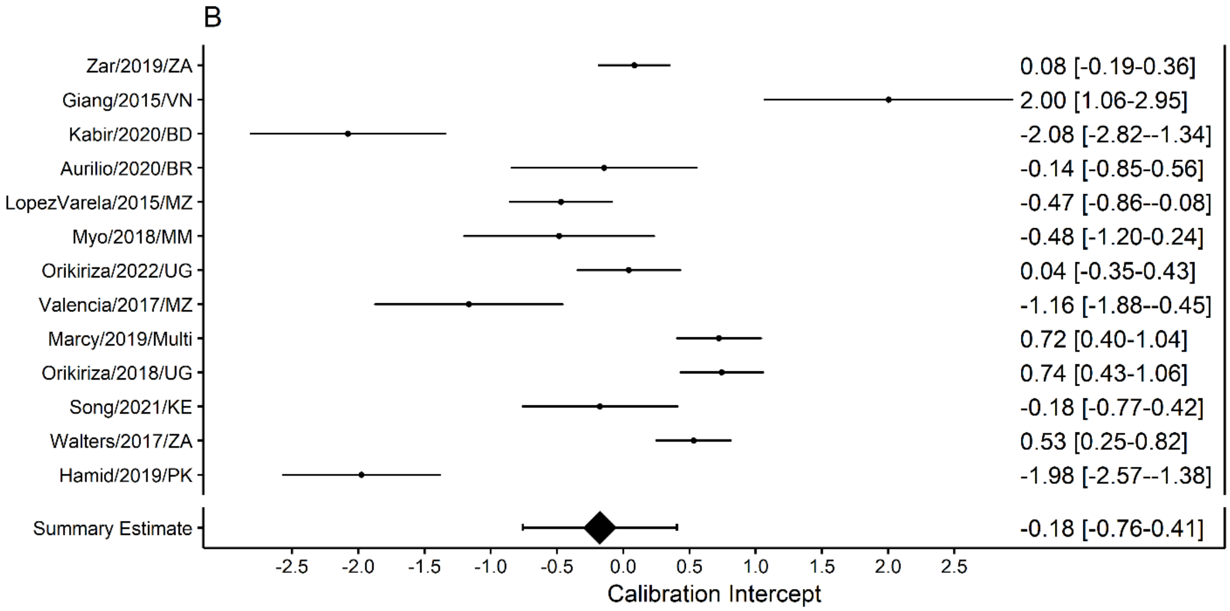
B
Zar/2019/ZA
Giang/2015/VN
Kabir/2020/BD
Aurilio/2020/BR
LopezVarela/2015/MZ
Myo/2018/MM
Orikiriza/2022/UG
Valencia/2017/MZ
Marcy/2019/Multi
Orikiriza/2018/UG
Song/2021/KE
Walters/2017/ZA
Hamid/2019/PK
Summary Estimate
0.08 [-0.19-0.36]
2.00 [1.06-2.95]
-2.08 [-2.82--1.34]
-0.14 [-0.85-0.56]
-0.47 [-0.86--0.08]
-0.48 [-1.20-0.24]
0.04 [-0.35-0.43]
-1.16 [-1.88--0.45]
0.72 [0.40-1.04]
0.74 [0.43-1.06]
-0.18 [-0.77-0.42]
0.53 [0.25-0.82]
-1.98 [-2.57--1.38]
-0.18 [-0.76-0.41]
-2.5 -2.0 -1.5 -1.0 -0.5 0.0 0.5 1.0 1.5 2.0 2.5
Calibration Intercept

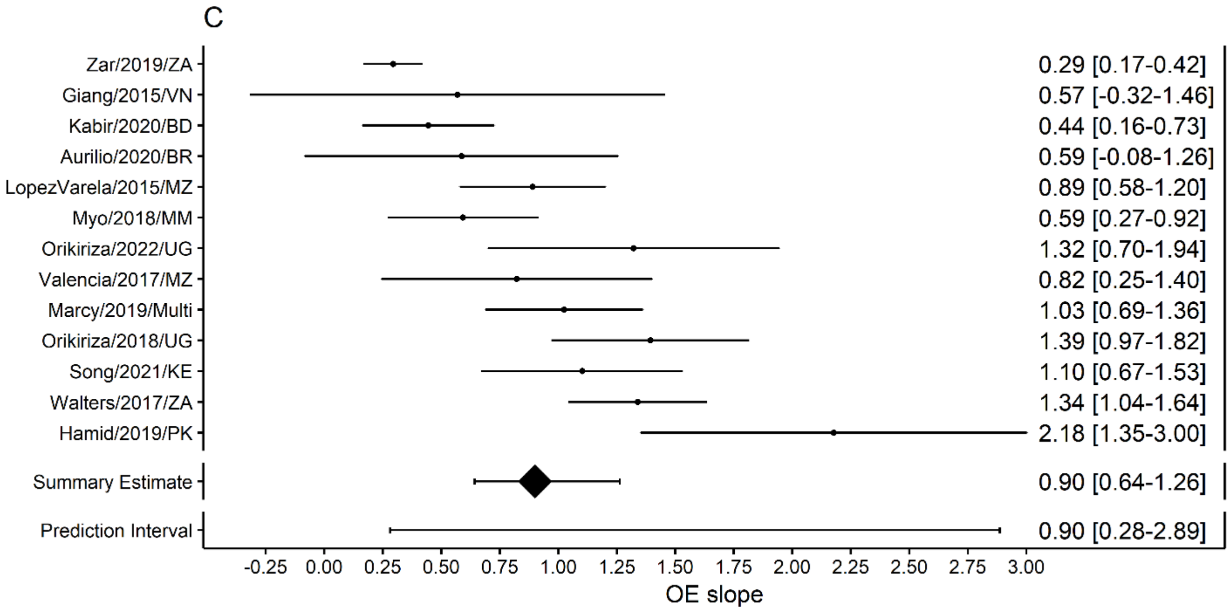
C
Zar/2019/ZA
Giang/2015/VN
Kabir/2020/BD
Aurilio/2020/BR
LopezVarela/2015/MZ
Myo/2018/MM
Orikiriza/2022/UG
Valencia/2017/MZ
Marcy/2019/Multi
Orikiriza/2018/UG
Song/2021/KE
Walters/2017/ZA
Hamid/2019/PK
Summary Estimate
Prediction Interval
0.29 [0.17-0.42]
0.57 [-0.32-1.46]
0.44 [0.16-0.73]
0.59 [-0.08-1.26]
0.89 [0.58-1.20]
0.59 [0.27-0.92]
1.32 [0.70-1.94]
0.82 [0.25-1.40]
1.03 [0.69-1.36]
1.39 [0.97-1.82]
1.10 [0.67-1.53]
1.34 [1.04-1.64]
2.18 [1.35-3.00]
0.90 [0.64-1.26]
0.90 [0.28-2.89]
-0.25 0.00 0.25 0.50 0.75 1.00 1.25 1.50 1.75 2.00 2.25 2.50 2.75 3.00
OE slope

Appendix R: Logistic regression model, calibration, and discrimination of prediction model developed from IPD without CXR features

Table S51. Estimates of logistic regression prediction model developed from IPD without CXR features. Parameter estimate with 95% confidence intervals, odds ratio estimate with 95% confidence interval, and p-values for each parameter included in the logistic regression prediction model. The estimate provided for each predictor is computed against a reference that reflects the absence of that feature. The model parameter estimates account for potential clustering at the study-level as well as uncertainty introduced by missing data. *P-value calculated using Rubin's rules for multiple imputed data. TB – tuberculosis, IPD – individual participant data, OR – odds ratio, CI – confidence interval, CXR – chest x-ray.

Predictors	Coefficient	Coefficient 95% CI	OR	OR 95% CI	P-value*
Intercept	-1·36	-1·94, -0·78	--	--	--
Cough duration ≥ 2 weeks (Absence is no cough or cough <2 weeks)	0·22	-0·03, 0·47	1·25	0·97, 1·6	0·75
Fever duration ≥ 2 weeks (Absence is no fever or fever <2 weeks)	0·46	0·18, 0·73	1·58	1·2, 2·07	0·21
Lethargy	0·2	-0·01, 0·41	1·22	0·99, 1·51	0·75
Weight loss	0·24	0·01, 0·48	1·28	1·01, 1·62	0·68
History of documented TB exposure	1·33	0·81, 1·84	3·76	2·24, 6·31	<0·001
Haemoptysis	0·4	-0·27, 1·06	1·49	0·76, 2·89	0·70
Night sweats	0·28	0·12, 0·45	1·33	1·12, 1·57	0·43
Peripheral lymphadenopathy	0·32	0·12, 0·52	1·38	1·13, 1·68	0·40
Temperature ≥38 degrees Celsius	0·01	-0·22, 0·23	1·01	0·8, 1·26	>0·999
Tachycardia	0·19	-0·08, 0·47	1·21	0·92, 1·6	0·83
Tachypnoea	0·07	-0·18, 0·33	1·08	0·84, 1·39	0.97

Figures S20. Forest plot depicting discrimination and calibration of prediction model without chest x-ray features to classify TB. Study-level and pooled estimates of the **(A)** discrimination (c-statistic) and calibration [**(B**) calibration intercept and (**C**) O:E ratio] of the prediction model developed from the IPD in classifying TB using an internal-external cross-validation framework (reference standard: bacteriologically-confirmed pulmonary TB and unconfirmed pulmonary TB). c-Statistic – concordance statistic, O:E – observed: expected ratio
, IPD – individual participant data, TB – tuberculosis, BD – Bangladesh, BR – Brazil, KE – Kenya, MM – Myanmar, Multi – Multi-country study (includes Burkina Faso, Cameroon, Vietnam, and Cambodia), MZ – Mozambique, PK – Pakistan, UG – Uganda, VN – Vietnam, ZA – South Africa.

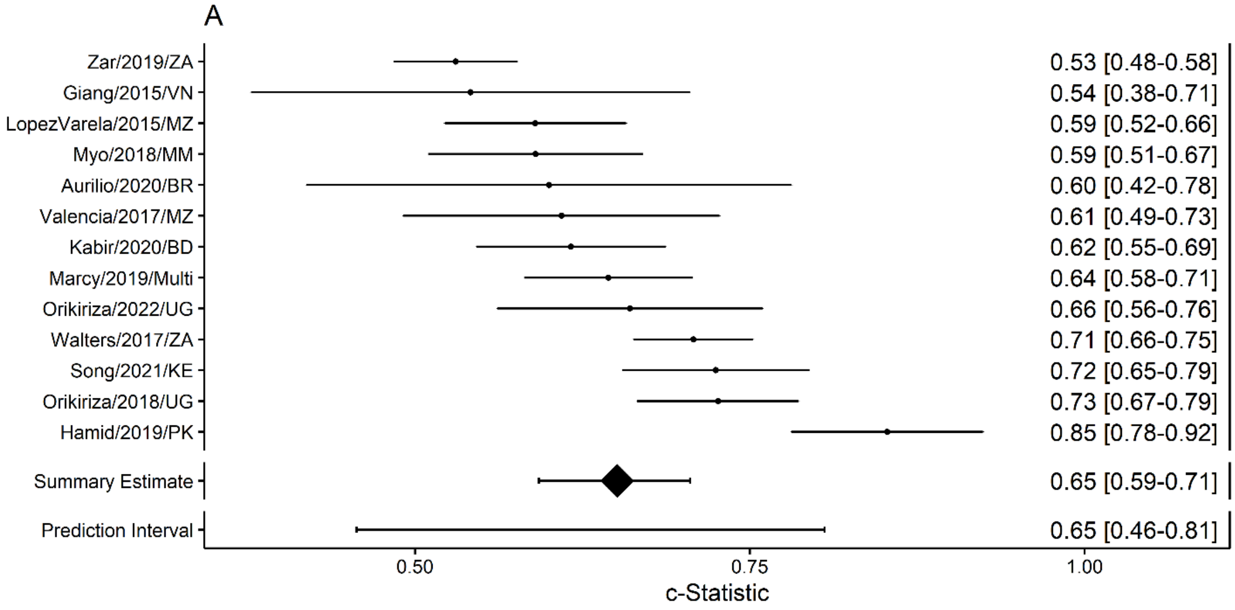

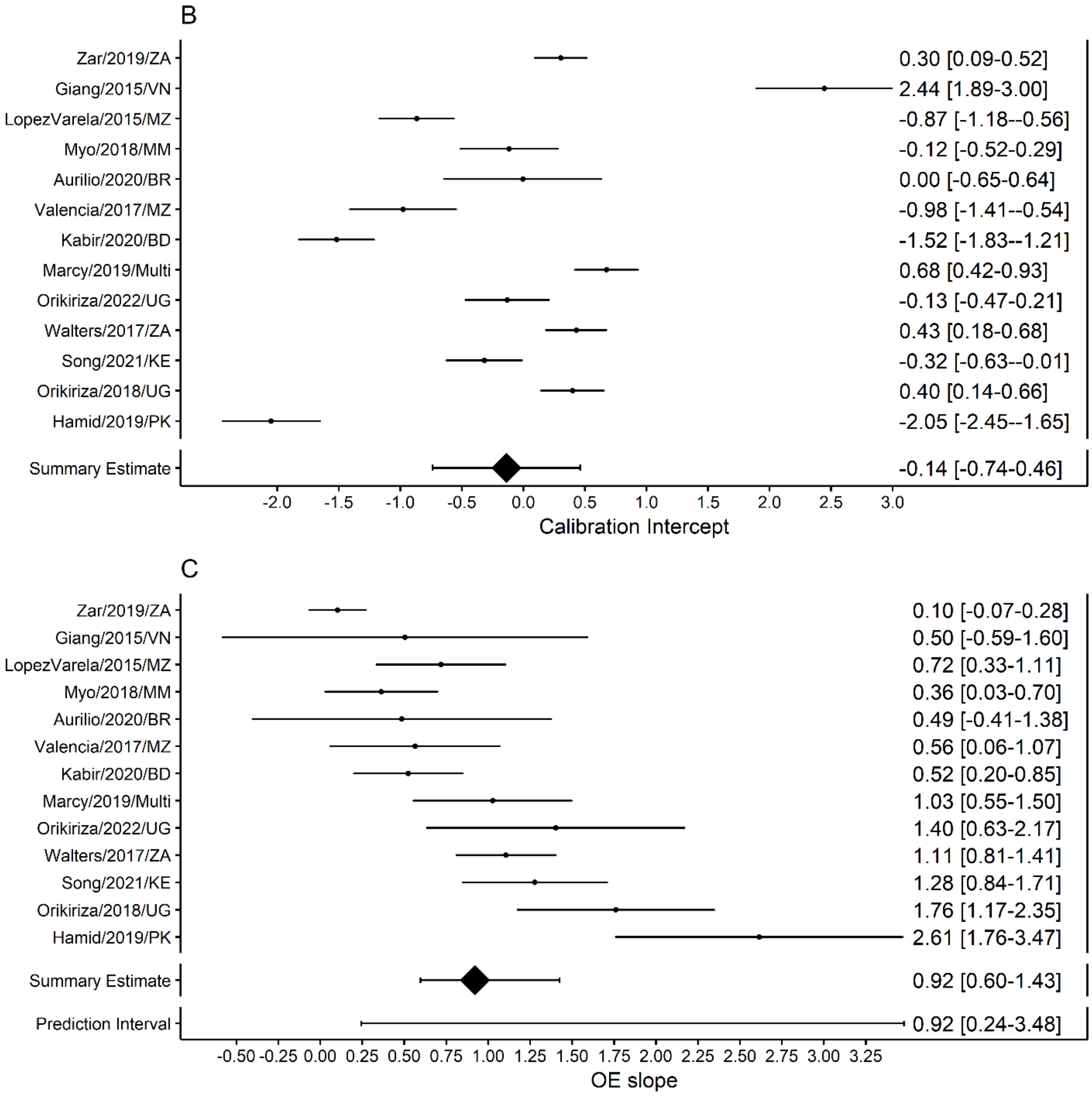
B
Zar/2019/ZA
Giang/2015/VN
LopezVarela/2015/MZ
Myo/2018/MM
Aurilio/2020/BR
Valencia/2017/MZ
Kabir/2020/BD
Marcy/2019/Multi
Orikiriza/2022/UG
Walters/2017/ZA
Song/2021/KE
Orikiriza/2018/UG
Hamid/2019/PK
Summary Estimate
0.30 [0.09-0.52]
2.44 [1.89-3.00]
-0.87 [-1.18--0.56]
-0.12 [-0.52-0.29]
0.00 [-0.65-0.64]
-0.98 [-1.41--0.54]
-1.52 [-1.83--1.21]
0.68 [0.42-0.93]
-0.13 [-0.47-0.21]
0.43 [0.18-0.68]
-0.32 [-0.63--0.01]
0.40 [0.14-0.66]
-2.05 [-2.45--1.65]
-0.14 [-0.74-0.46]
-2.0 -1.5 -1.0 -0.5 0.0 0.5 1.0 1.5 2.0 2.5 3.0
Calibration Intercept
C
Zar/2019/ZA
Giang/2015/VN
LopezVarela/2015/MZ
Myo/2018/MM
Aurilio/2020/BR
Valencia/2017/MZ
Kabir/2020/BD
Marcy/2019/Multi
Orikiriza/2022/UG
Walters/2017/ZA
Song/2021/KE
Orikiriza/2018/UG
Hamid/2019/PK
Summary Estimate
Prediction Interval
0.10 [-0.07-0.28]
0.50 [-0.59-1.60]
0.72 [0.33-1.11]
0.36 [0.03-0.70]
0.49 [-0.41-1.38]
0.56 [0.06-1.07]
0.52 [0.20-0.85]
1.03 [0.55-1.50]
1.40 [0.63-2.17]
1.11 [0.81-1.41]
1.28 [0.84-1.71]
1.76 [1.17-2.35]
2.61 [1.76-3.47]
0.92 [0.60-1.43]
0.92 [0.24-3.48]
-0.50 -0.25 0.00 0.25 0.50 0.75 1.00 1.25 1.50 1.75 2.00 2.25 2.50 2.75 3.00 3.25
OE slope

Appendix S: Prediction model fit and scaled scores at different sensitivity thresholds

Table S52. OR and 95% CI from prediction model developed from IPD and corresponding scaled scores.

Predictors	Coefficient	Coefficient 95% CI	OR	OR 95% CI	P-value*	Score at 90% sens.	Score at 85% sens.	Score at 80% sens.	Score at 75% sens.	Score at 70% sens.
Intercept	-1·92	-2·58, -1·25	--	--	--	--	--	--	--	--
Cough duration ≥ 2 weeks (Absence is no cough or cough <2 weeks)	0·17	-0·09, 0·43	1·18	0·91, 1·54	0·86	3	2	2	1	1
Fever duration ≥ 2 weeks (Absence is no fever or fever <2 weeks)	0·45	0·16, 0·74	1·57	1·18, 2·09	0·25	7	5	4	4	3
Lethargy	0·25	0·02, 0·48	1·28	1·02, 1·62	0·66	4	3	2	2	2
Weight loss	0·22	-0·03, 0·48	1·25	0·97, 1·61	0·75	3	3	2	2	2
History of documented TB exposure	1·43	0·87, 2	4·19	2·38, 7·38	<0·001	22	17	14	12	10
Haemoptysis	0·34	-0·37, 1·05	1·4	0·69, 2·86	0·79	5	4	3	3	2
Night sweats	0·2	0·02, 0·38	1·22	1·02, 1·47	0·71	3	2	2	2	1
Peripheral lymphadenopathy	0·35	0·13, 0·57	1·42	1·14, 1·77	0·35	5	4	3	3	2
Temperature ≥38 degrees Celsius	0	-0·25, 0·26	1	0·78, 1·3	>0·999	0	0	0	0	0
Tachycardia	0·15	-0·13, 0·42	1·16	0·88, 1·53	0·90	2	2	1	1	1
Tachypnoea	-0·05	-0·27, 0·16	0·95	0·77, 1·18	0·98	-1	-1	-1	0	0
Cavities on CXR	0·47	-0·11, 1·05	1·6	0·9, 2·85	0·53	7	6	5	4	3
Intrathoracic lymphadenopathy on CXR	1·46	1, 1·92	4·32	2·73, 6·85	<0·001	23	17	14	12	10
Opacities on CXR	0·43	0·02, 0·84	1·54	1·02, 2·32	0·45	7	5	4	4	3
Miliary infiltrate on CXR	1·27	0·57, 1·97	3·56	1·76, 7·19	<0·001	20	15	12	10	9
Pleural effusion on CXR	0·64	0·2, 1·09	1·9	1·22, 2·96	0·13	10	8	6	5	4

Table S53. OR and 95% CI from prediction model without chest x-ray features developed from IPD and corresponding scaled scores.

Predictors	Coefficient	Coefficient 95% CI	OR	OR 95% CI	P-value*	Score at 90% sens.	Score at 85% sens.	Score at 80% sens.	Score at 75% sens.	Score at 70% sens.
Intercept	-1·36	-1·94, -0·78	--	--	--	--	--	--	--	--
Cough duration ≥ 2 weeks (Absence is no cough or cough <2 weeks)	0·22	-0·03, 0·47	1·25	0·97, 1·6	0·75	6	5	4	3	3
Fever duration ≥ 2 weeks (Absence is no fever or fever <2 weeks)	0·46	0·18, 0·73	1·58	1·2, 2·07	0·21	13	10	8	7	6
Lethargy	0·2	-0·01, 0·41	1·22	0·99, 1·51	0·75	6	4	4	3	3
Weight loss	0·24	0·01, 0·48	1·28	1·01, 1·62	0·68	7	5	4	4	3
History of documented TB exposure	1·33	0·81, 1·84	3·76	2·24, 6·31	<0·001	39	29	24	20	17
Haemoptysis	0·4	-0·27, 1·06	1·49	0·76, 2·89	0·70	12	9	7	6	5
Night sweats	0·28	0·12, 0·45	1·33	1·12, 1·57	0·43	8	6	5	4	4
Peripheral lymphadenopathy	0·32	0·12, 0·52	1·38	1·13, 1·68	0·40	9	7	6	5	4
Temperature ≥38 degrees Celsius	0·01	-0·22, 0·23	1·01	0·8, 1·26	>0·999	0	0	0	0	0
Tachycardia	0·19	-0·08, 0·47	1·21	0·92, 1·6	0·83	6	4	3	3	2
Tachypnoea	0·07	-0·18, 0·33	1·08	0·84, 1·39	0·97	2	2	1	1	1

Appendix T: Performance of scores from prediction model at different sensitivity thresholds

Figure S21. (A) sensitivity and (B) specificity of score developed from prediction model to classify TB with 90% sensitivity.

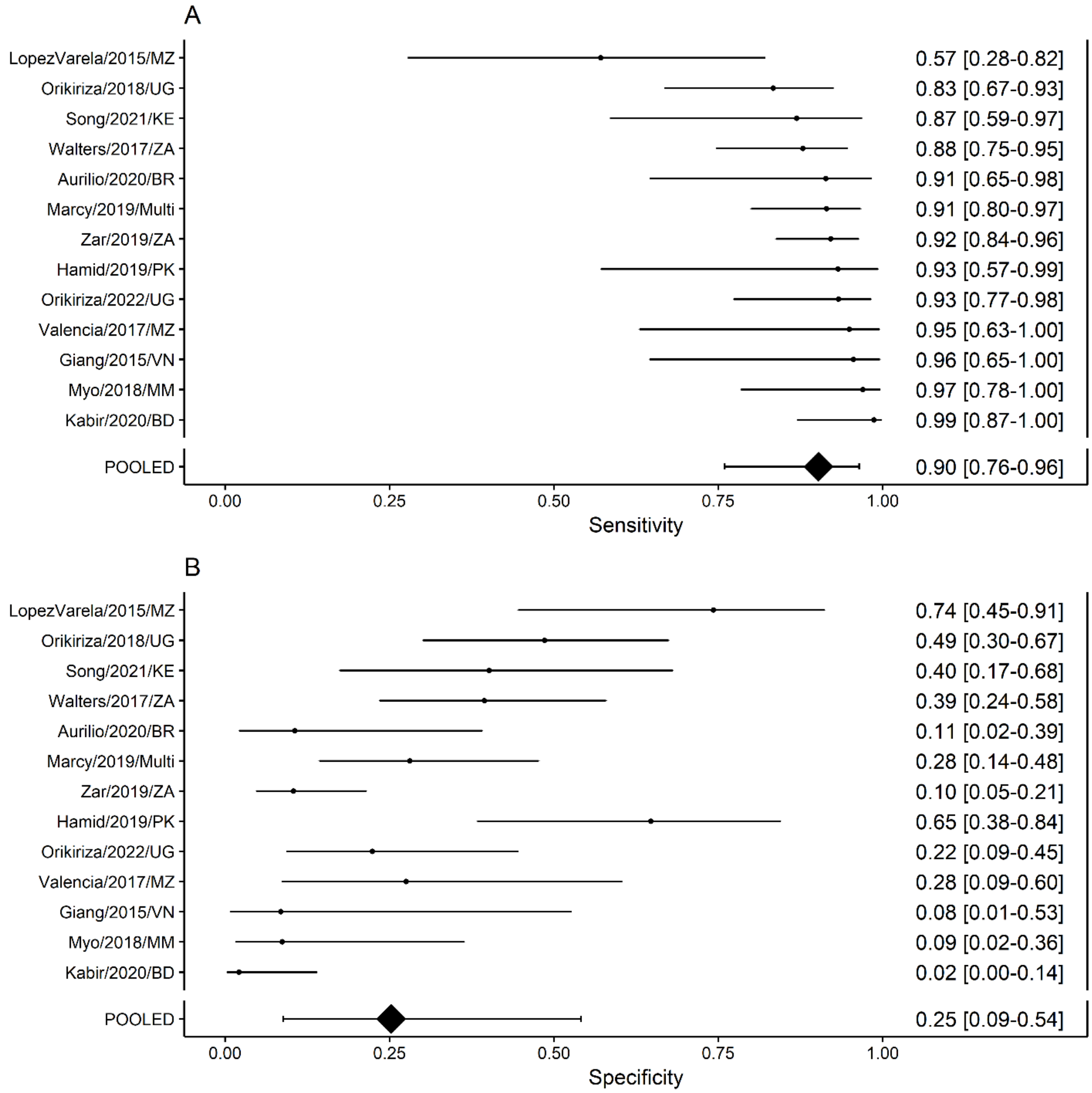

Figure S22. (A) sensitivity and (B) specificity of score developed from prediction model to classify TB with 85% sensitivity. Presented in the main text.

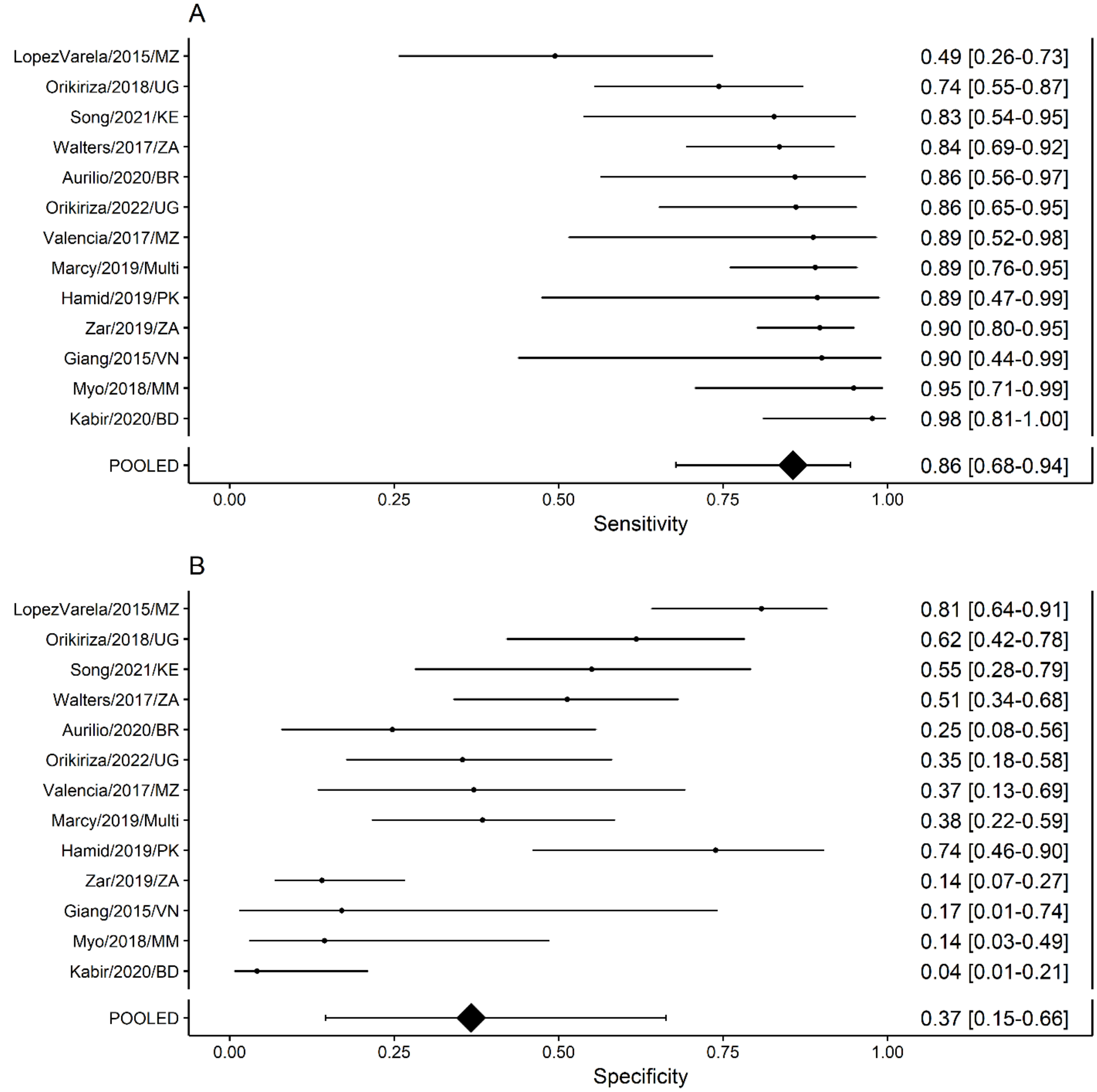

Figure S23. (A) sensitivity and (B) specificity of score developed from prediction model to classify TB with 80% sensitivity.

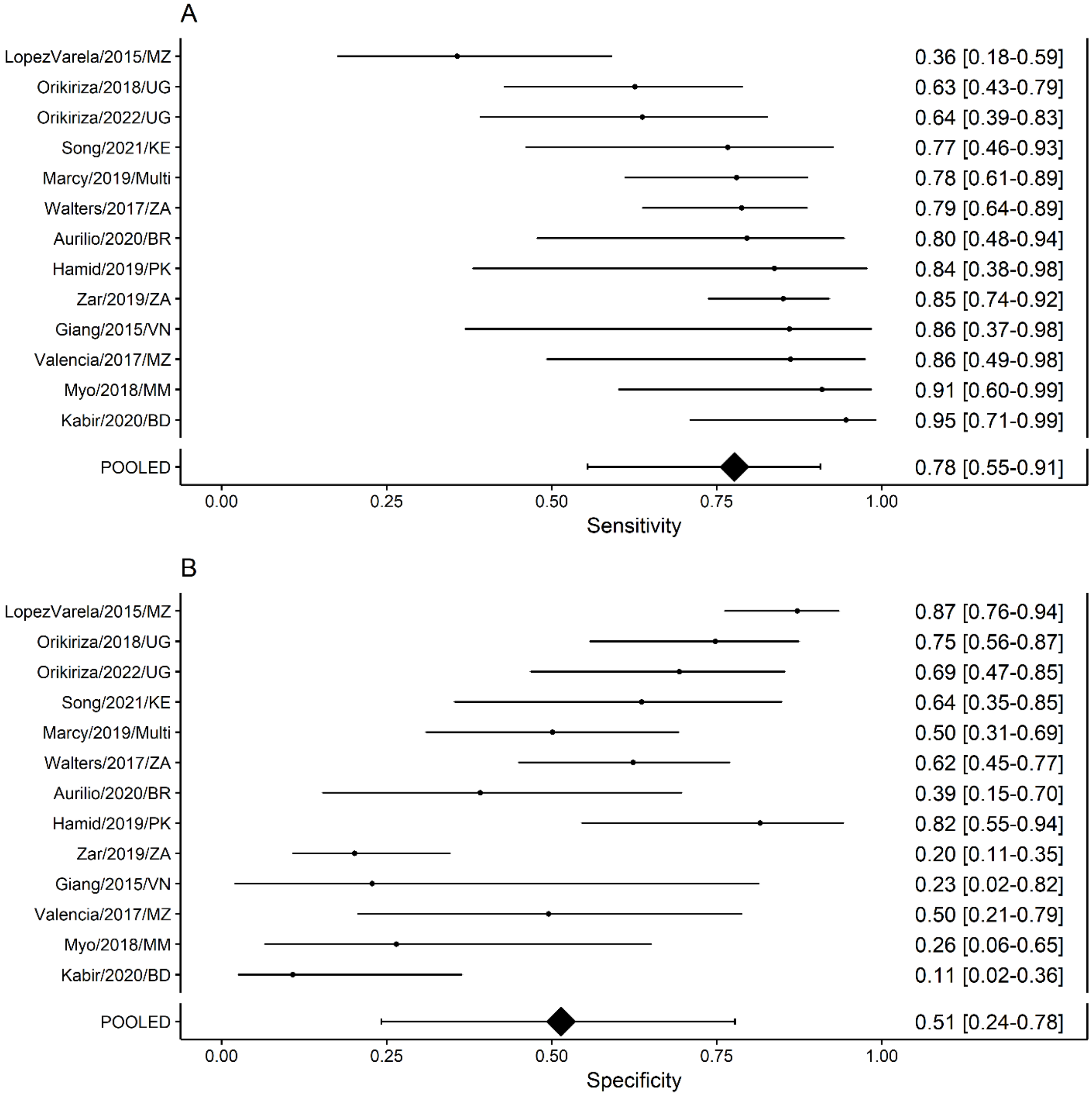

Figure S24. (A) sensitivity and (B) specificity of score developed from prediction model to classify TB with 75% sensitivity.

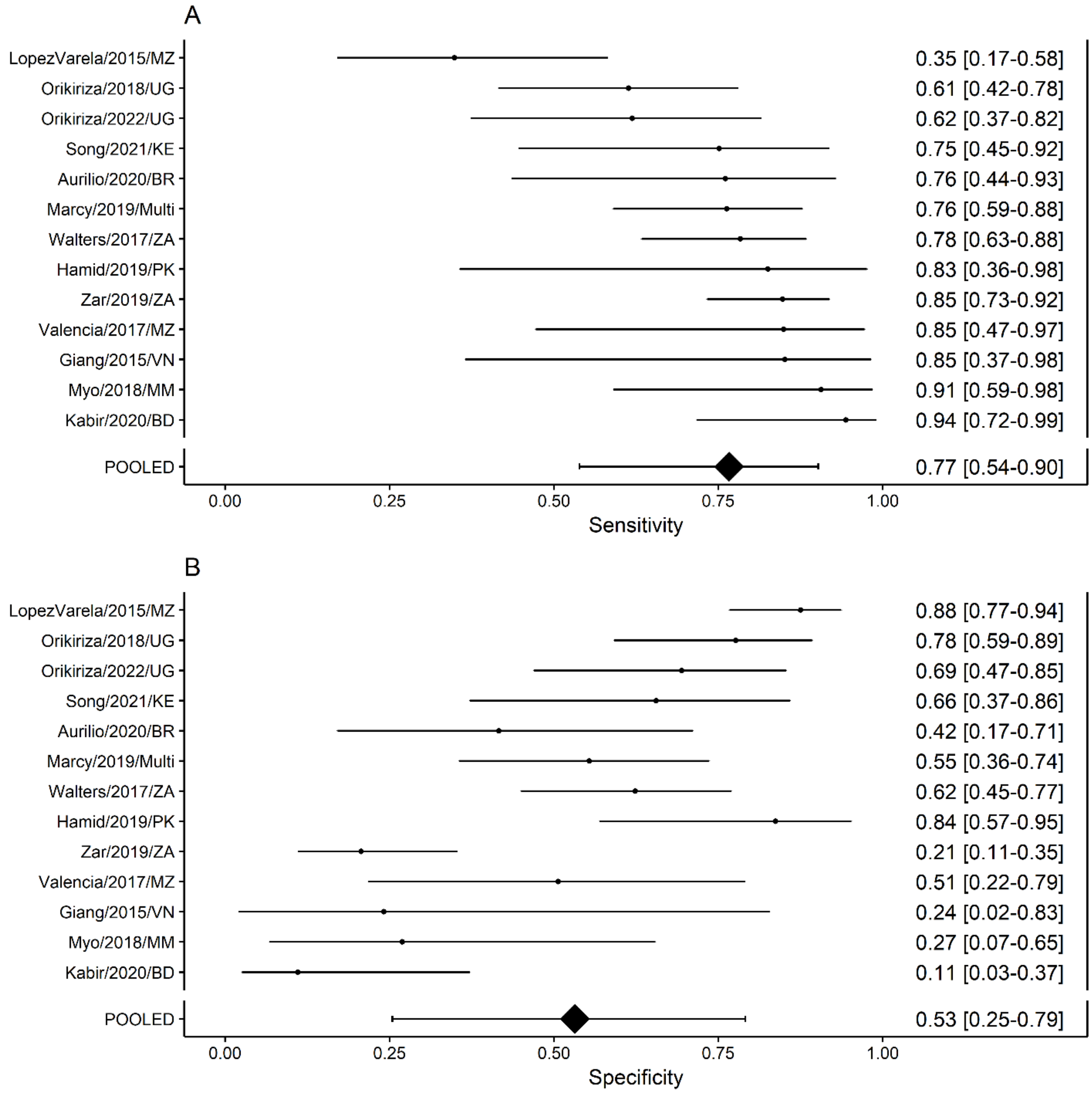

Figure S25. (A) sensitivity and (B) specificity of score developed from prediction model to classify TB with 70% sensitivity.

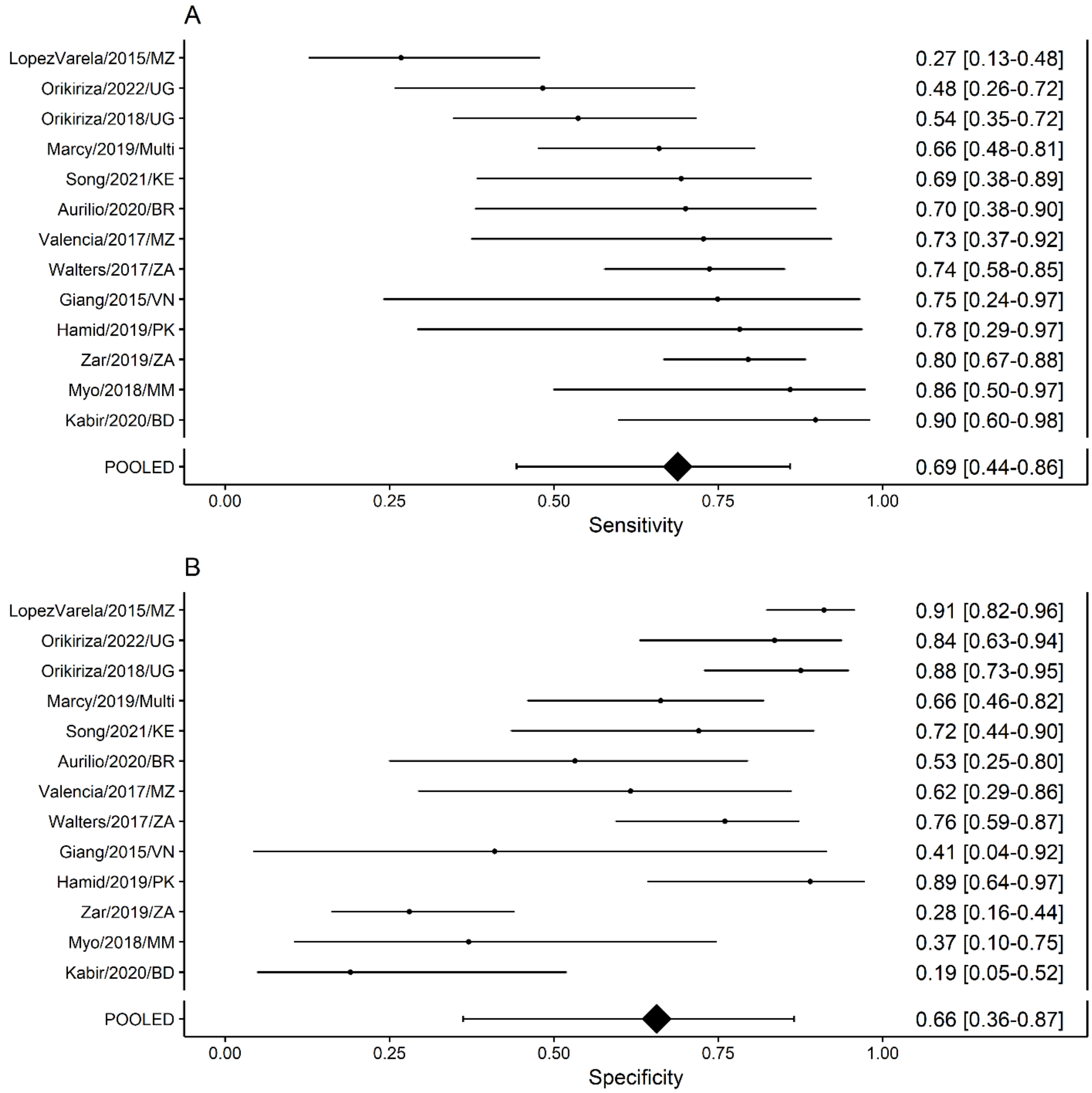

Appendix U: Performance of scores to classify TB with 85% sensitivity using confirmed TB reference standard

Figure S26. (A) sensitivity and (B) specificity of score developed from prediction model to classify bacteriologically-confirmed TB with 85% sensitivity. Analysis excludes data from children with unconfirmed TB.

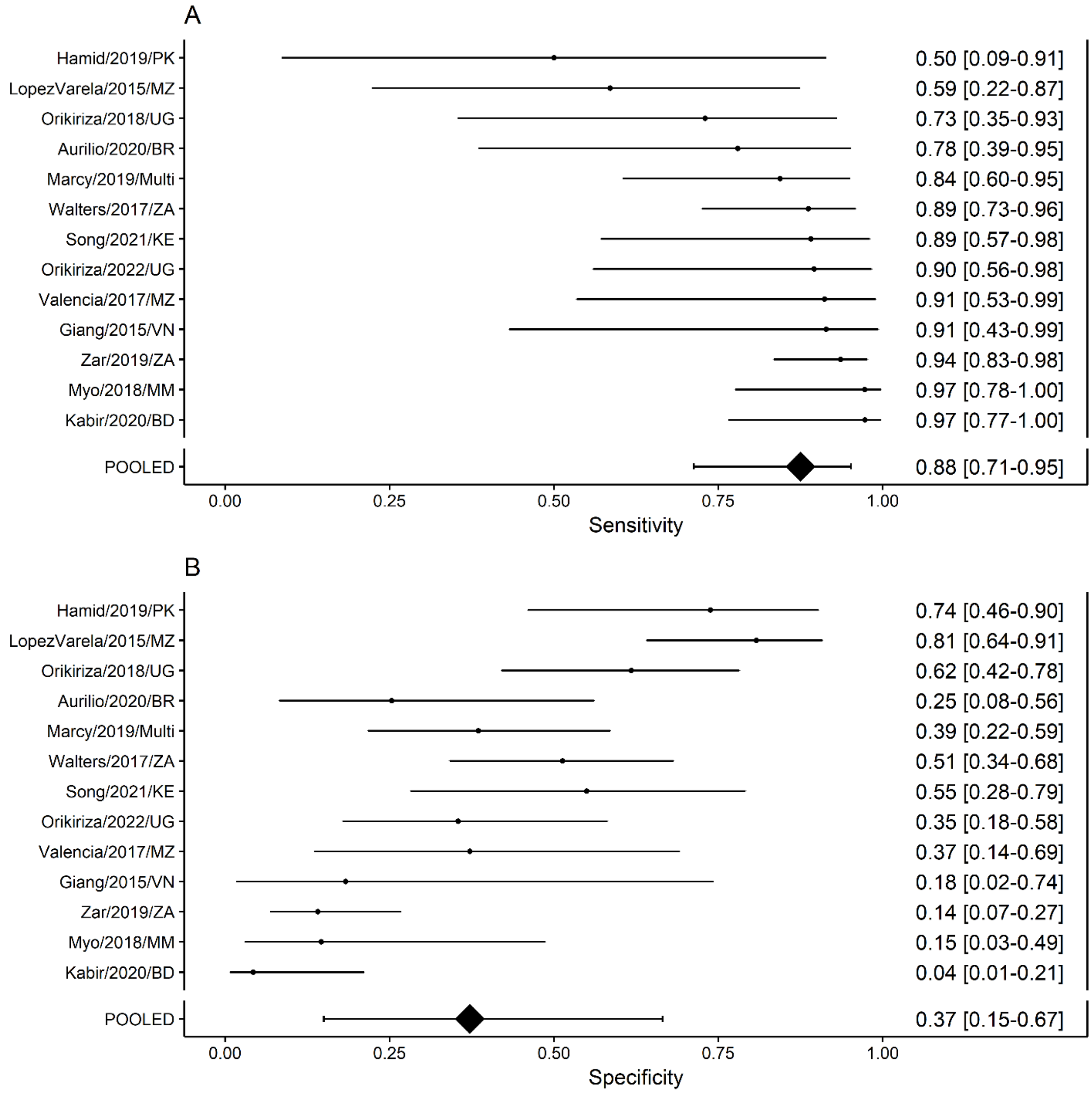

Appendix V: Performance of scores to classify TB with 85% sensitivity (without chest X-ray features)

Figure S27. (A) sensitivity and (B) specificity of score developed from prediction model without chest x-ray to classify TB using composite reference standard with 85% sensitivity.

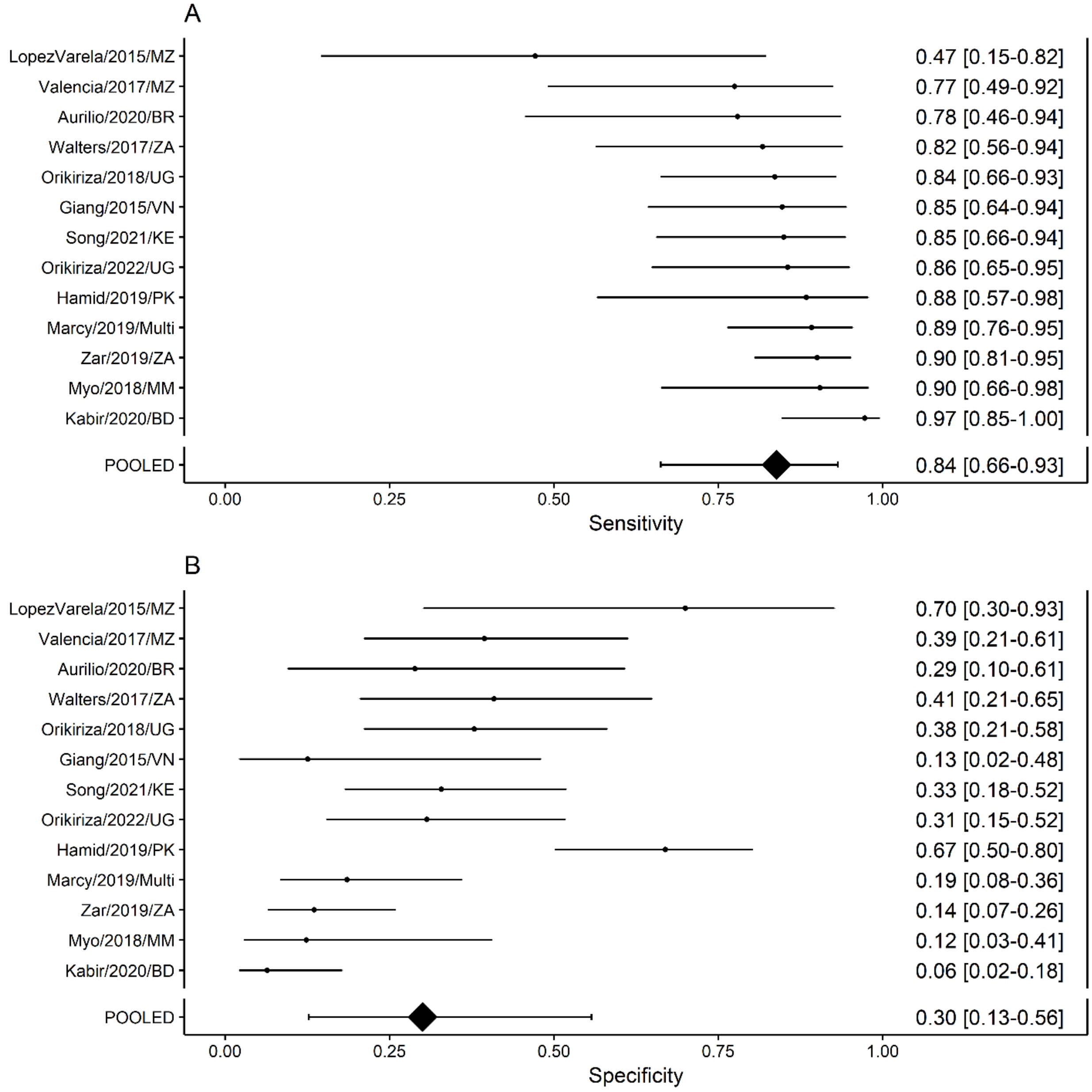

Figure S28. (A) sensitivity and (B) specificity of score developed from prediction model without chest x-ray to classify bacteriologically-confirmed TB with 85% sensitivity. Analysis excludes data from children with unconfirmed TB.

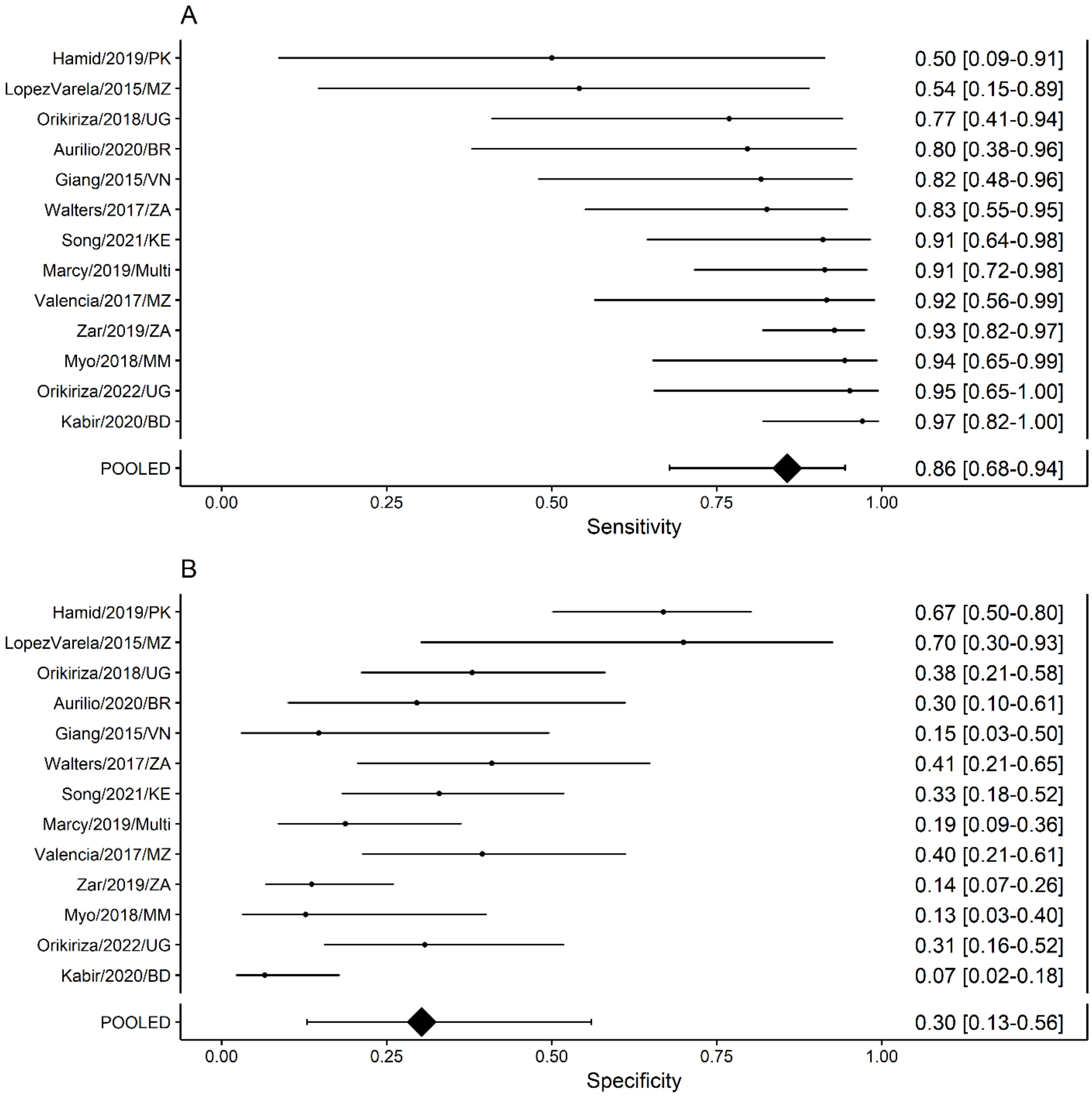

Appendix W: Inspire algorithm without CXR features

Figure S29. Treatment-decision algorithm derived from prediction model without CXR features. Scores associated with features from clinical history and physical exam and chest X-ray translate to risk of TB and are scaled from the prediction model developed from the IPD. Guidance on the practical use of this algorithm is outlined in the WHO operational handbook.[29] WHO – World Health Organization, TB – tuberculosis, IPD – individual participant data, HIV – human immunodeficiency virus, mWRD – molecular WHO-recommended rapid diagnostic test, CLHIV – children living with HIV, LF-LAM – lateral flow urine lipoarabinomannan assay, CXR – chest X-ray.

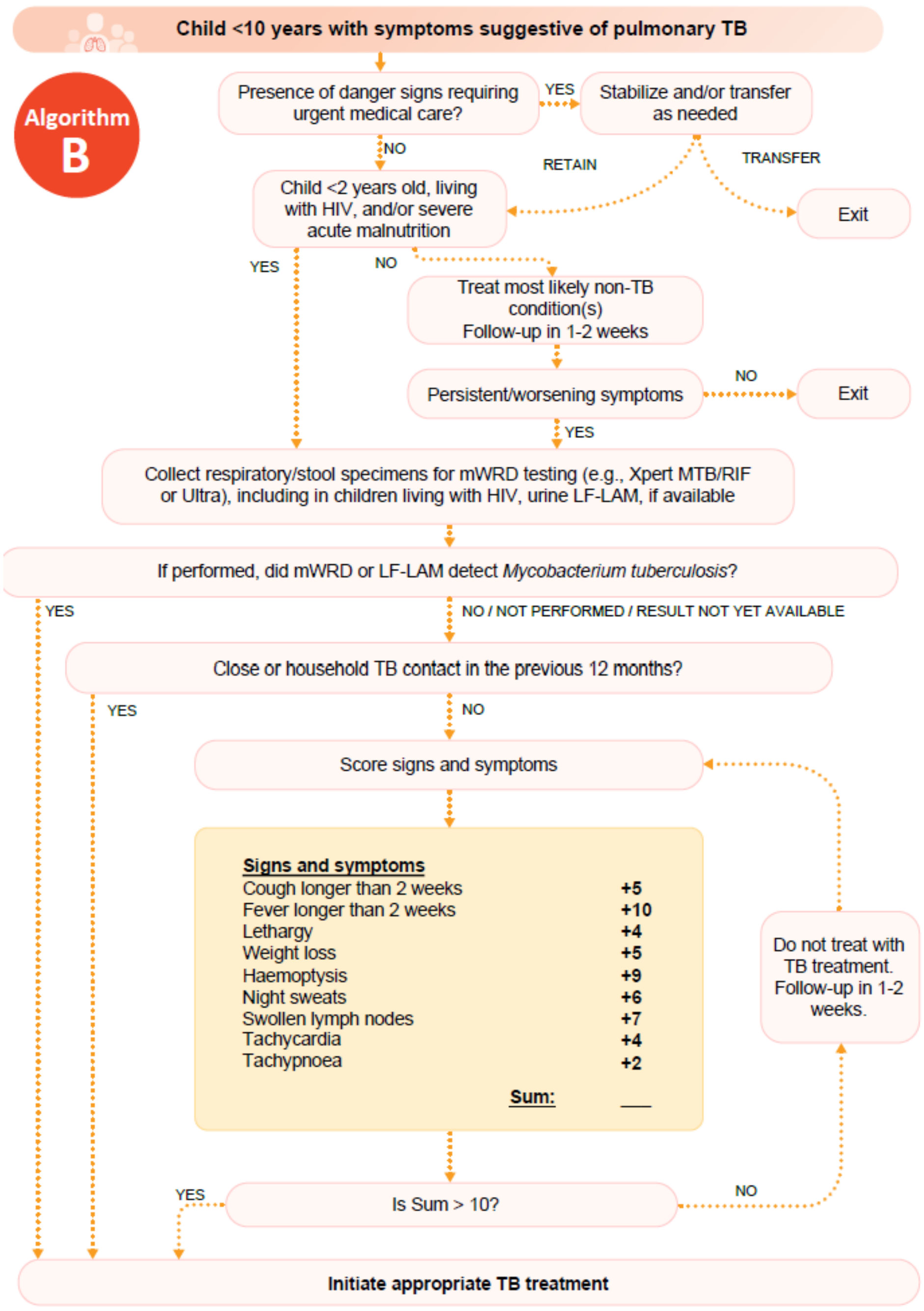

Appendix X: TRIPOD Checklist

Table S54. TRIPOD Checklist[30] Page numbers preceded with an "S" refer to the pages within the supplement.

Section/Topic			**Checklist Item**	**Page**
Title and abstract				
Title	**1**	**D;V**	**Identify the study as developing and/or validating a multivariable prediction model, the target population, and the outcome to be predicted.**	1
Abstract	**2**	**D;V**	**Provide a summary of objectives, study design, setting, participants, sample size, predictors, outcome, statistical analysis, results, and conclusions.**	1
Introduction				
Background and objectives	**3a**	**D;V**	**Explain the medical context (including whether diagnostic or prognostic) and rationale for developing or validating the multivariable prediction model, including references to existing models.**	2-3
	**3b**	**D;V**	**Specify the objectives, including whether the study describes the development or validation of the model or both.**	3
Methods				
Source of data	**4a**	**D;V**	**Describe the study design or source of data (e.g., randomized trial, cohort, or registry data), separately for the development and validation data sets, if applicable.**	3, S12-S65
	**4b**	**D;V**	**Specify the key study dates, including start of accrual; end of accrual; and, if applicable, end of follow-up.**	S12-S24
Participants	**5a**	**D;V**	**Specify key elements of the study setting (e.g., primary care, secondary care, general population) including number and location of centres.**	3, S12-S24
	**5b**	**D;V**	**Describe eligibility criteria for participants.**	3, S12-S24
	**5c**	**D;V**	**Give details of treatments received, if relevant.**	NA
Outcome	**6a**	**D;V**	**Clearly define the outcome that is predicted by the prediction model, including how and when assessed.**	3-4, S12-S24
	**6b**	**D;V**	**Report any actions to blind assessment of the outcome to be predicted.**	S12-S24
Predictors	**7a**	**D;V**	**Clearly define all predictors used in developing or validating the multivariable prediction model, including how and when they were measured.**	S3-S6, S12-S37, S65
	**7b**	**D;V**	**Report any actions to blind assessment of predictors for the outcome and other predictors.**	NA
Sample size	**8**	**D;V**	**Explain how the study size was arrived at.**	NA
Missing data	**9**	**D;V**	**Describe how missing data were handled (e.g., complete-case analysis, single imputation, multiple imputation) with details of any imputation method.**	3, S7-S8
Statistical analysis methods	**10a**	**D**	**Describe how predictors were handled in the analyses.**	3-4
	**10b**	**D**	**Specify type of model, all model-building procedures (including any predictor selection), and method for internal validation.**	3-4
	**10c**	**V**	**For validation, describe how the predictions were calculated.**	NA
	**10d**	**D;V**	**Specify all measures used to assess model performance and, if relevant, to compare multiple models.**	4
	**10e**	**V**	**Describe any model updating (e.g., recalibration) arising from the validation, if done.**	NA
Risk groups	**11**	**D;V**	**Provide details on how risk groups were created, if done.**	NA
Development vs. validation	**12**	**V**	**For validation, identify any differences from the development data in setting, eligibility criteria, outcome, and predictors.**	NA
Results				
Participants	**13a**	**D;V**	**Describe the flow of participants through the study, including the number of participants with and without the outcome and, if applicable, a summary of the follow-up time. A diagram may be helpful.**	5, S12-S24
	**13b**	**D;V**	**Describe the characteristics of the participants (basic demographics, clinical features, available predictors), including the number of participants with missing data for predictors and outcome.**	5, S38-S63
	**13c**	**V**	**For validation, show a comparison with the development data of the distribution of important variables (demographics, predictors and outcome).**	NA
Model development	**14a**	**D**	**Specify the number of participants and outcome events in each analysis.**	4,5
	**14b**	**D**	**If done, report the unadjusted association between each candidate predictor and outcome.**	NA
Model specification	**15a**	**D**	**Present the full prediction model to allow predictions for individuals (i.e., all regression coefficients, and model intercept or baseline survival at a given time point).**	6
	**15b**	**D**	**Explain how to the use the prediction model.**	6
Model performance	**16**	**D;V**	**Report performance measures (with CIs) for the prediction model.**	5
Model-updating	**17**	**V**	**If done, report the results from any model updating (i.e., model specification, model performance).**	NA
Discussion				

Limitations	**18**	**D;V**	**Discuss any limitations of the study (such as nonrepresentative sample, few events per predictor, missing data).**	8-9
Interpretation	**19a**	**V**	**For validation, discuss the results with reference to performance in the development data, and any other validation data.**	NA
	**19b**	**D;V**	**Give an overall interpretation of the results, considering objectives, limitations, results from similar studies, and other relevant evidence.**	8-9
Implications	**20**	**D;V**	**Discuss the potential clinical use of the model and implications for future research.**	9
Other information				
Supplementary information	**21**	**D;V**	**Provide information about the availability of supplementary resources, such as study protocol, Web calculator, and data sets.**	NA
Funding	**22**	**D;V**	**Give the source of funding and the role of the funders for the present study.**	5,10, S12-S24

Appendix Y: Supplementary references

1. van Buuren S, Groothuis-Oudshoorn K. mice: Multivariate imputation by chained equations in R. *J Stat Softw* 2011; **45**(3): 1-67.
2. Robitzsch A, Grund S. miceadds: some additional multiple imputation functions, especially for 'mice'. R package version 3.13-12, https://CRAN.R-project.org/package=miceadds. 2022.
3. Reitsma JB, Glas AS, Rutjes AWS, Scholten RJPM, Bossuyt PM, Zwinderman AH. Bivariate analysis of sensitivity and specificity produces informative summary measures in diagnostic reviews. *J Clin Epidemiol* 2005; **58**(10): 982-90.
4. Heymans MW, Eekhout I. Applied missing data analysis with SPSS and (R)Studio; 2019.
5. Debray T, de Jong V. metamisc: meta-analysis of diagnosis and prognosis research studies. R package version 0.2.4/r584. https://R-Forge.R-project.org/projects/metamisc/. 2021.
6. Kabir S, Rahman SMM, Ahmed S, et al. Xpert Ultra assay on stool to diagnose pulmonary tuberculosis in children. *Clin Infect Dis* 2020.
7. Graham SM, Cuevas LE, Jean-Philippe P, et al. Clinical case definitions for classification of intrathoracic tuberculosis in children: an update. *Clin Infect Dis* 2015; **61**: s179-87.
8. Aurilio RB, Luiz RR, Land MGP, Cardoso CAA, Kritski AL, Sant Anna CC. The clinical and molecular diagnosis of childhood and adolescent pulmonary tuberculosis in referral centers. *Rev Soc Bras Med Trop* 2020; **53**.
9. Song R, Click ES, McCarthy KD, et al. Sensitive and feasible specimen collection and testing strategies for diagnosing tuberculosis in young children. *JAMA Pediatr* 2021: e206069-e.
10. Graham SM, Ahmed T, Amanullah F, et al. Evaluation of tuberculosis diagnostics in children: 1. Proposed clinical case definitions for classification of intrathoracic tuberculosis disease. Consensus from an expert panel. *J Infect Dis* 2012; **205 Suppl 2**(Suppl 2): S199-208.
11. Garcia-Basteiro AL, Lopez-Varela E, Augusto OJ, et al. Radiological findings in young children investigated for tuberculosis in Mozambique. *PLoS One* 2015; **10**(5): e0127323.
12. Valencia S, Respeito D, Blanco S, et al. Tuberculosis drug resistance in Southern Mozambique: results of a population-level survey in the district of Manhiça. *Int J Tuberc Lung Dis* 2017; **21**(4): 446-51.
13. Myo K, Zaw M, Swe TL, et al. Evaluation of Xpert® MTB/RIF assay as a diagnostic test for pulmonary tuberculosis in children in Myanmar. *Int J Tuberc Lung Dis* 2018; **22**(9): 1051-5.
14. Marcy O, Borand L, Ung V, et al. A treatment-decision score for HIV-infected children with suspected tuberculosis. *Pediatrics* 2019; **144**(3): e20182065.
15. Hamid M, Brooks MB, Madhani F, et al. Risk factors for unsuccessful tuberculosis treatment outcomes in children. *PLoS One* 2019; **14**(9): e0222776.
16. Zar HJ, Workman LJ, Prins M, et al. Tuberculosis diagnosis in children using Xpert Ultra on different respiratory specimens. *Am J Respir Crit Care Med* 2019.
17. Walters E, van der Zalm MM, Palmer M, et al. Xpert MTB/RIF on stool is useful for the rapid diagnosis of tuberculosis in young children with severe pulmonary disease. *Pediatr Infect Dis J* 2017; **36**(9): 837-43.
18. Orikiriza P, Nansumba M, Nyehangane D, et al. Xpert MTB/RIF diagnosis of childhood tuberculosis from sputum and stool samples in a high TB-HIV-prevalent setting. *Eur J Clin Microbiol Infect Dis* 2018; **37**(8): 1465-73.
19. Orikiriza P, Smith J, Ssekyanzi B, et al. Tuberculosis diagnostic accuracy of stool Xpert MTB/RIF and urine AlereLAM in vulnerable children. *Eur Respir J* 2022; **59**(1).
20. Giang do C, Duong TN, Ha DT, et al. Prospective evaluation of GeneXpert for the diagnosis of HIV-negative pediatric TB cases. *BMC Infect Dis* 2015; **15**: 70.
21. Wells GA, Shea B, O'Connell D, et al. The Newcastle-Ottawa Scale (NOS) for assessing the quality of nonrandomised studies in meta-analyses https://www.ohri.ca/programs/clinical_epidemiology/oxford.asp.
22. Marais BJ, Gie RP, Hesseling AC, et al. A refined symptom-based approach to diagnose pulmonary tuberculosis in children. *Pediatrics* 2006; **118**(5): e1350-9.
23. Graham S. The Union's desk guide for diagnosis and management of TB in children. 3 ed. Paris, France: International Union Against Tuberculosis and Lung Disease; 2016.
24. Montenegro SH, Gilman RH, Sheen P, et al. Improved detection of Mycobacterium tuberculosis in Peruvian children by use of a heminested IS6110 polymerase chain reaction assay. *Clin Infect Dis* 2003; **36**(1): 16-23.
25. Zawedde-Muyanja S, Nakanwagi A, Dongo JP, et al. Decentralisation of child tuberculosis services increases case finding and uptake of preventive therapy in Uganda. *Int J Tuberc Lung Dis* 2018; **22**(11): 1314-21.

26. Brasil. Ministério da Saúde. Secretaria de Vigilância em Saúde. Departamento de Vigilância das Doenças Transmissíveis. Manual de recomendações para o controle da tuberculose no Brasil. 2. ed: Brasília: Ministério da Saúde; 2019.
27. Edwards K. The diagnosis of childhood tuberculosis. *P N G Med J* 1987; **30**(2): 169-78.
28. Gunasekera KS, Walters E, van der Zalm MM, et al. Development of a treatment-decision algorithm for human immunodeficiency virus-uninfected children evaluated for pulmonary tuberculosis. *Clin Infect Dis* 2021; **73**(4): e904-e12.
29. World Health Organization. WHO operational handbook on tuberculosis. Module 5: management of tuberculosis in children and adolescents. Geneva: World Health Organization; 2022.
30. Collins GS, Reitsma JB, Altman DG, Moons KGM. Transparent reporting of a multivariable prediction model for individual prognosis or diagnosis (TRIPOD): the TRIPOD statement. *Br Med J* 2015; **350**: g7594.
